# One-Step HF-Free Synthesis of Alkali Metal Fluorides from Fluorspar

**DOI:** 10.1021/jacs.4c16608

**Published:** 2025-02-18

**Authors:** Thomas Schlatzer, Christopher A. Goult, Michael A. Hayward, Véronique Gouverneur

**Affiliations:** † Department of Chemistry, Chemistry Research Laboratory, University of Oxford, Oxford OX1 3TA, United Kingdom; ‡ Department of Chemistry, Inorganic Chemistry Laboratory, University of Oxford, Oxford OX1 3QR, United Kingdom

## Abstract

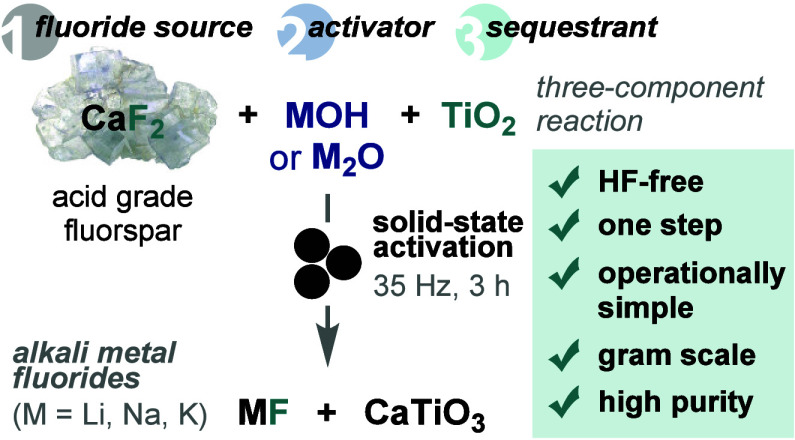

Alkali metal fluorides (MF) are commodity chemicals currently synthesized from naturally occurring fluorite (fluorspar, CaF_2_) in two steps: conversion of acid grade fluorspar (AGF) into highly hazardous hydrogen fluoride (HF) followed by neutralization with alkali metal hydroxides/carbonates. Herein, we report a one-step mechanochemical reaction that converts AGF into alkali metal fluorides under basic conditions, bypassing HF. The method consists of reacting AGF with alkali metal (hydr)oxides and titanium dioxide (TiO_2_) under mechanical energy for MF formation and *in situ* sequestration of calcium (hydr)oxide byproducts as calcium titanate (CaTiO_3_). Ca^2+^ sequestration prevents reversible CaF_2_ formation upon aqueous extraction, thus enabling the isolation of alkali metal fluorides. We also demonstrate that alkali metal titanates (M_2_TiO_3_) are suitable reagents for both CaF_2_ activation and Ca^2+^ sequestration, with K_2_TiO_3_ being optimal for KF synthesis.

Fluorochemicals have found applications in various sectors including materials, agrochemicals, and pharmaceuticals.^1,2^ The fluorine atoms incorporated in fluorochemicals are derived from fluorite (fluorspar, CaF_2_), a naturally occurring mineral mined predominantly in China, Mexico, and South Africa.^3^ Synthetic protocols toward fluorochemicals involve multiple stages all, starting with the conversion of acid grade fluorspar (AGF) into hydrogen fluoride (HF) upon treatment with concentrated sulfuric acid under harsh conditions (>300 °C; Figure 1A).^4,5^ Large-scale operation of this process is centralized due to the expertise required to produce and handle highly hazardous HF.

**Figure 1:**
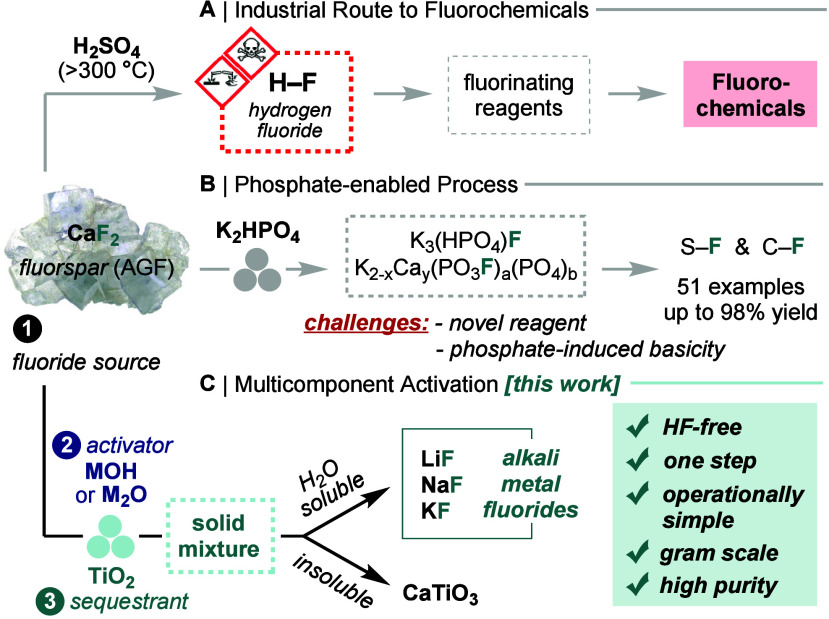
Strategies for activating acid grade fluorspar (AGF). (A) Current industrial supply chain for fluorochemicals via HF. (B) S–F and C–F bond formation using phosphate-activated AGF. (C) Mechanochemical activation of AGF with alkali metal (hydr)oxides and TiO_2_ for direct access to LiF, NaF, and KF (this work).

Recently, we demonstrated that fluorochemicals are accessible from fluorspar via an operationally simple protocol bypassing the production and high-maintenance supply chain of dangerous HF. We opted for basic instead of conventional acidic conditions and developed a phosphate-enabled mechanochemical process that converts AGF into a mixture of crystalline phases identified as K_3_(HPO_4_)F and K_2–*x*_Ca_*y*_(PO_3_F)_a_(PO_4_)_b_, a novel fluorinating reagent suitable for forging S–F and C–F bonds (Figure 1B).^6^ Various sulfonyl and alkyl fluorides were prepared in good yields with this reagent, but limitations were encountered when exploring its reactivity further, partially due to the presence of phosphate. Building on this mechanochemical process, we surmised that the direct conversion of AGF into commonly used fluorochemicals would represent a significant advance by offering a direct link with the fluorochemical industry (Figure 1C). The preparation of alkali metal fluorides was given priority due to increasing demand in a range of applications including lithium-ion batteries (LiF), water treatment and dental care (NaF), and fluorination chemistry (KF).^5,7,8^

For exploration, we selected NaF (*U*_POT_^BHFC^ = 930 kJ·mol^–1^, BHFC = Born–Haber–Fajans cycle), a salt of lattice energy intermediate between LiF (1049 kJ·mol^–1^) and KF (829 kJ·mol^–1^).^9^ We envisioned that the solid-state reaction of AGF with NaOH applying mechanical energy would represent the most direct route to NaF upon separation of the Ca(OH)_2_ byproduct or derivatives thereof. The conversion of CaF_2_ (2651 kJ·mol^–1^) into Ca(OH)_2_ (2637 kJ·mol^–1^) incurs some energetic cost, which is compensated by the higher lattice energy of NaF (930 kJ·mol^–1^) compared to NaOH (892 kJ·mol^–1^). The overall reaction is thus thermodynamically favorable with a net increase in lattice energies (Δ*U*_POT_^BHFC^ = +62 kJ·mol^–1^). Using Na_2_O as the activator, the energetic gain is more pronounced (Δ*U*_POT_^BHFC^ = +132 kJ·mol^–1^), given the higher lattice energy of CaO (3401 kJ·mol^–1^) versus that of Ca(OH)_2_.

Encouraged by the arguments above, we examined the reactivity of AGF with NaOH (2.0 equiv) under ball milling (35 Hz, 3 h). Powder X-ray diffraction (PXRD) analysis (Cu Kα_1,2_ radiation) of the reaction mixture showed diagnostic reflections of NaF and Ca(OH)_2_. Reaction with Na_2_O (1.0 equiv) led to the formation of NaF alongside CaO, in this case with no detectable reflections for unreacted CaF_2_. Solid-state ^19^F qNMR indicated that reaction of AGF with either NaOH or Na_2_O afforded NaF (δ_F_ = −224 ppm) in 78% and 82% yield, respectively. Although realization of this strategy seems straightforward and represents an attractive and direct route to NaF, implementation requires the separation of NaF from Ca(OH)_2_ or CaO. This process is challenging because extraction of the crude solid mixture with water returned CaF_2_ due to its lower solubility (16 mg·kg^–1^ at 25 *°*C) versus Ca(OH)_2_ (1.5 g·kg^–1^ at 25 *°*C).^9−11^ Indeed, analysis by ^19^F qNMR (10% D_2_O in H_2_O) of the water-soluble fraction after ball milling revealed low levels of soluble fluoride (δ_F_ = −120 ppm) quantified as 8% and 9% for NaOH and Na_2_O, respectively. As anticipated, the solid precipitating in water was unambiguously identified by PXRD as CaF_2_ (Figure S1).

These findings prompted the development of a refined protocol whereby the reaction of AGF with NaOH/Na_2_O is carried out in the presence of an oxide acceptor (Lux-Flood acid).^12,13^ This third component captures Ca(OH)_2_/CaO *in situ*, in order to prevent reversible CaF_2_ formation (Figure 2A). An early report on the mechanochemical reaction of calcium (hydr)oxide with metal oxides (M_a_O_b_) provided guidance.^14^ Mixtures of AGF (1.0 equiv), NaOH (2.0 equiv), and M_a_O_b_ (1.0 equiv) were subjected to ball milling (35 Hz, 3 h) followed by PXRD analysis (Table S2). Titanium dioxide (TiO_2_, polymorph mixture), a natural mineral of low toxicity,^5^ stood out as the best candidate by sequestering Ca(OH)_2_ efficiently as the perovskite calcium titanate (CaTiO_3_). Under these conditions, the amount of fluoride extractable in water increased from 8% to 81%. The alternative metal oxides Al_2_O_3_, SiO_2_, V_2_O_5_, ZrO_2_, HfO_2_, and WO_3_ showed no or minor improvement compared with the two-component reaction. Al_2_O_3_, V_2_O_5_, and WO_3_ led to the formation of inert sodium metalates (NaAlO_2_, NaVO_3_, and Na_2_WO_4_) resulting from reaction with NaOH instead of Ca(OH)_2_. With ZrO_2_ and HfO_2_, the fluoride release did not exceed 17%. SiO_2_ was partially effective (32% extractable fluoride) but inferior to TiO_2_ because CaSiO_3_ reacts with NaF (Figure S2). In contrast, neither TiO_2_, nor CaTiO_3_, nor CaF_2_ react with NaF under standard mechanochemical conditions (Figure 2B, Table S6). Further optimization with TiO_2_ revealed that multiple ball bearings are important for high fluoride release (1 × 7 g ball, 49% vs 2 × 7 g balls, 81%), while the jar loading did not significantly impact reaction efficiency (Tables S7, S8). Readily available NaOH (2.0 equiv, 81% F^–^ release) was as effective as Na_2_O (1.0 equiv, 79% F^–^ release) in this three-component process. *Ex situ* time-course analysis by PXRD and ^19^F qNMR (10% D_2_O in H_2_O) indicated that NaF formation was stalling after 3 h at 30 Hz or 2 h at 35 Hz (Figure 2C). Noteworthy, comparable reactivity (85% F^–^ release) was observed in a planetary ball mill using zirconia jars and bearings (Table S11). Further improvement featured NaOH as the limiting component (1.8 equiv), a modification enabling its full consumption for facile NaF purification (84% yield, 94% purity, Table S12). Next, we developed a protocol to isolate multigram quantities of NaF from AGF. Separation of NaF was straightforward because CaTiO_3_ is insoluble in water.^15^ Three-component mixtures (1.0 equiv CaF_2_, 1.8 equiv NaOH, 1.0 equiv TiO_2_) were ball milled in parallel in six stainless steel jars (1.00 g loading each), followed by aqueous extraction and filtration. Under these conditions, NaF is the only soluble species, with the water-insoluble matrix consisting of CaTiO_3_ and unreacted CaF_2_. Evaporation of the filtrate enabled the isolation of 1.65 g of NaF (84% yield, >98% purity) (Figure 2D). Higher jar loadings (2.00 g per jar, 22 jars) afforded 10.73 g of NaF (74% yield, >99% purity). Purities were determined by ^19^F qNMR and elemental analysis (Tables S18, S19).

**Figure 2:**
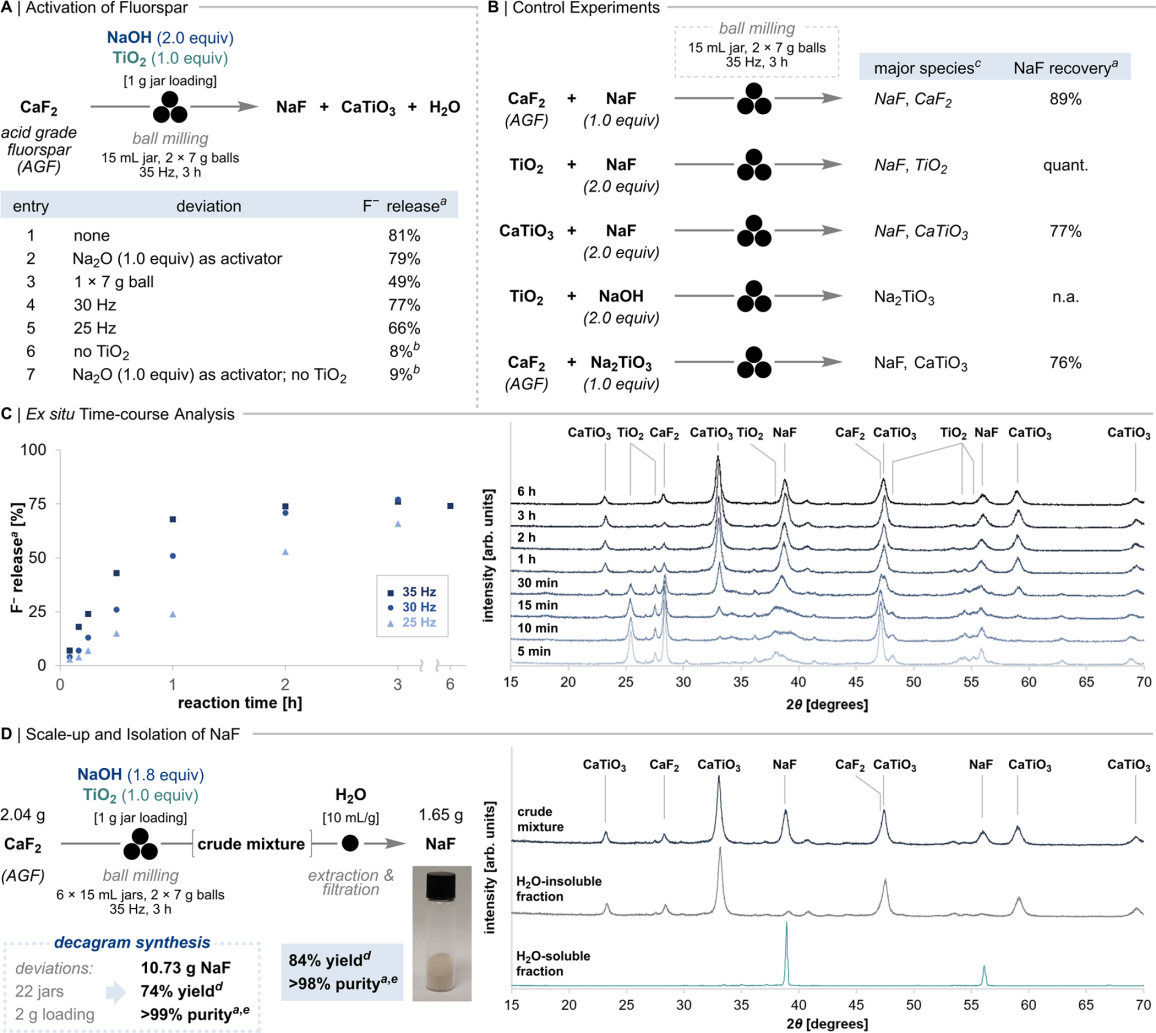
Multicomponent activation of AGF using NaOH and TiO_2_ polymorphs. (A) Reaction optimization with respect to extractable fluoride. (B) Two-component control reactions. (C) Time-course analysis of the three-component reaction (1.0 equiv CaF_2_, 2.0 equiv NaOH, 1.0 equiv TiO_2_) by ^19^F qNMR for different milling frequencies (left) and by PXRD with major crystalline species annotated for milling at 35 Hz (right). (D) One-step synthesis and isolation of NaF from AGF. ^*a*^Determined by ^19^F qNMR (10% D_2_O in H_2_O); n.a. = not applicable. ^*b*^Low amount of aqueous fluoride due to reversible CaF_2_ formation. ^*c*^Determined by PXRD. ^*d*^Calculated based on NaOH as the limiting component. ^*e*^Determined by elemental analysis.

Control experiments were performed to shed light on this three-component activation of AGF. NaOH was found to react with TiO_2_ or AGF at frequencies as low as 20–25 Hz, affording Na_2_TiO_3_ and NaF alongside Ca(OH)_2_, respectively. Consumption of Ca(OH)_2_ by TiO_2_ into CaTiO_3_ also proceeded at 20–25 Hz. A smaller particle size of TiO_2_ (<100 nm) was beneficial for fluoride release, and experiments carried out with pure anatase or rutile showed reactivity similar to polymorph mixtures (Table S4). Na_2_TiO_3_ reacted with AGF forming NaF and CaTiO_3_ above 20–25 Hz. In contrast, NaAlO_2_, Na_2_SiO_3_, NaVO_3_, or Na_2_WO_4_ did not react or were less effective in producing NaF (Table S3). A plausible mechanistic pathway may therefore involve the formation of NaF and Ca(OH)_2_ with the *in situ* capture of Ca(OH)_2_ by TiO_2_ as CaTiO_3_. Alternatively, Na_2_TiO_3_ may form by reaction of NaOH with TiO_2_, which can react subsequently with CaF_2_ furnishing NaF and CaTiO_3_.

Having developed a robust protocol for the direct synthesis of NaF from AGF, we investigated its use in the preparation of LiF and KF (Figure 3A). Thermodynamically, the reaction of CaF_2_ with LiOH or Li_2_O results in a net increase of lattice energies (Δ*U*_POT_^BHFC^ = +28 kJ·mol^–1^ and +34 kJ·mol^–1^, respectively). Treatment of AGF with 2.0 equiv of LiOH under ball milling conditions (35 Hz, 3 h) resulted in a solid with PXRD reflections characteristic of LiF, Ca(OH)_2_, and trace amounts of CaF_2_. Solid-state ^19^F qNMR confirmed LiF formation (δ_F_ = −204 ppm) in 98% yield (97% yield using 1.0 equiv Li_2_O). These results compare to 44% LiF when applying the three-component protocol (1.0 equiv CaF_2_, 2.0 equiv LiOH, 1.0 equiv TiO_2_). A higher yield of 93% LiF was observed with 1.0 equiv of Li_2_O instead of LiOH. Performing this reaction in parallel with 6 jars (1.00 g loading each) using Li_2_O as activator (1.0 equiv) and TiO_2_ as sequestrant (1.0 equiv) afforded LiF isolated in 71% yield (1.17 g) with 99% purity as determined by elemental analysis (Figure 3B, Table S20).

**Figure 3:**
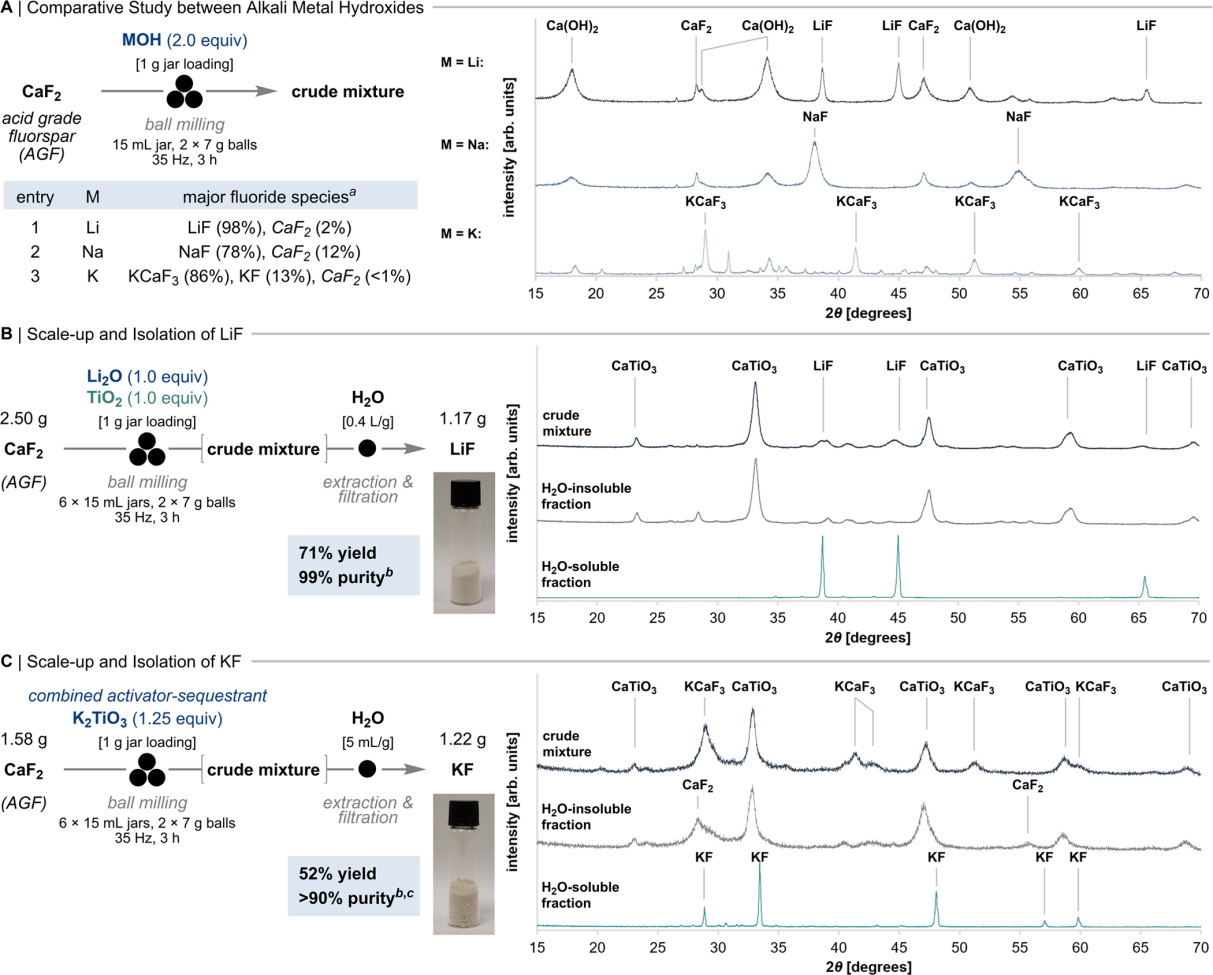
Synthesis of LiF and KF from AGF. (A) Reactivity of MOH with AGF. (B) One-step synthesis and isolation of LiF from AGF. (C) Synthesis of KF from AGF using K_2_TiO_3_. ^*a*^Determined by solid-state ^19^F qNMR. ^*b*^Determined by elemental analysis. ^*c*^Determined by ^19^F qNMR (10% D_2_O in H_2_O).

For KF synthesis, experiments were conducted with KOH only because K_2_O is not commercially available. KOH (2.0 equiv) reacted with AGF under mechanochemical conditions (35 Hz, 3 h) in line with thermodynamic predictions (Δ*U*_POT_^BHFC^ = +52 kJ·mol^–1^). CaF_2_ was consumed, but PXRD of the crude mixture revealed KF as minor product, while the fluoroperovskite KCaF_3_ was predominantly formed. Solid-state ^19^F qNMR quantified the distribution as 13% KF (δ_F_ = −133 ppm) and 86% KCaF_3_ (δ_F_ = −123 ppm). The formation of KCaF_3_ is consistent with the reactivity of KF and CaF_2_ under mechanical energy,^16^ in contrast to LiF and NaF, which are both inert toward CaF_2_ under standard mechanochemical conditions (Table S13). The three-component reaction (1.0 equiv CaF_2_, 2.0 equiv KOH, 1.0 equiv TiO_2_) afforded 95% KCaF_3_ as the sole fluorine-containing species according to solid-state ^19^F qNMR. Notably, ^19^F qNMR in solution (10% D_2_O in H_2_O) indicated the presence of 23% fluoride, suggesting partial reversibility in water. This was corroborated by the absence of KCaF_3_ reflections in the PXRD data from soluble and insoluble matter. A more detailed investigation of the reactivity of potassium salts ensued and revealed that potassium titanate (K_2_TiO_3_) is optimal, acting as both an activator for AGF and Ca^2+^ sequestrant. This salt, which is less hygroscopic than KOH, enabled the isolation of 1.22 g of KF (52% yield) in >90% purity as determined by ^19^F qNMR and elemental analysis (Figure 3C, Table S21). For comparison, Li_2_TiO_3_ and Na_2_TiO_3_ were found to be competent reagents for LiF and NaF synthesis, but priority was given to the economically more attractive three-component protocols (CaF_2_, LiOH/NaOH, TiO_2_).

In summary, we have developed a highly efficient protocol for the one-step synthesis of LiF, NaF, and KF from AGF. This chemistry represents a significant advance over conventional two-stage protocols that require the synthesis of hazardous HF, followed by neutralization with alkali metal hydroxides/carbonates. We demonstrated that the treatment of AGF with alkali metal (hydr)oxide and TiO_2_ under mechanical energy directly affords LiF or NaF in high yields. In this process, the alkali metal (hydr)oxide reacts with AGF, while TiO_2_ captures the calcium byproduct as CaTiO_3_, an essential step to overcome the reversible formation of CaF_2_ upon aqueous extraction, a process necessary to isolate MF products. For the synthesis of KF, the single reagent K_2_TiO_3_ is advantageous over the use of KOH and TiO_2_. In light of intensifying efforts to improve the chemical industry with global challenges in mind, the methodology disclosed herein enables the decentralized manufacturing of an important class of fluorochemicals by avoiding reliance on HF production.

## Supporting Information Available

Materials and methods, optimization studies, control and mechanistic experiments, detailed experimental procedures, and characterization data (PXRD traces, NMR spectra, TGA, elemental analyses) (PDF)
