# Supplementary material for: One-Step HF-Free Synthesis of Alkali Metal Fluorides from Fluorspar
Source: J Am Chem Soc. 2025 Feb 18;147(8):6338–42. doi: 10.1021/jacs.4c16608 (PMC11869288; doi:10.1021/jacs.4c16608)
Supplement: Supplementary file 1 — ja4c16608_si_001.pdf [file ja4c16608_si_001.pdf]

Supporting Information

One-Step HF-Free Synthesis of Alkali Metal Fluorides from Fluorspar

Thomas Schlatzer, Christopher A. Goult, Michael A. Hayward, Véronique Gouverneur*

*Correspondence to: veronique.gouverneur@chem.ox.ac.uk

Table of Contents

1. Materials and Methods 4

2. Optimization Studies on the Solid-State Activation of AGF 6

2.1. Screening of Sodium Salts as Activators 6

2.2. Reversibility of Hydroxide-mediated Activation of AGF 7

2.3. Screening of Sequestrants 8

2.4. Effect of TiO_2 Polymorphs and Particle Size 9

2.5. Effect of Water 10

2.6. Control Experiments (Selectivity/Compatibility of Components) 10

2.7. Optimization of Mechanochemical Parameters 12

2.7.1. Ball Bearings 12

2.7.2. Jar Loading 12

2.7.3. Milling Frequency 13

2.7.4. Milling Time and Milling Frequency 14

2.7.5. Optimization of Planetary Ball Milling 16

2.8. Optimization of Activator Stoichiometry 16

3. Preparation of Sodium Metallates 18

3.1. Sodium Aluminate 18

3.2. Sodium Metatitanate 18

3.3. Sodium Metavanadate 19

3.4. Sodium Tungstate 20

4. Mechanistic Investigations 21

4.1. Plausible Mechanistic Pathways and Putative Elementary Steps 21

4.2. Pathway A 21

4.3. Pathway B 23

5. Comparative Study between Alkali Metal Hydroxides 25

5.1. Intrinsic Stabilities of Alkali Metal Fluorides in Presence of AGF 25

5.2. Binary Systems for the Activation of AGF 25

5.3. Ternary Systems for the Activation of AGF 26

5.4. Alkali Metal Titanates as Activators for AGF 26

6. Solid-state NMR Spectroscopy 28

6.1. Binary Systems 28

6.2. Ternary Systems 33

7. Simulated PXRD Reference Patterns 36

8. Isolation and Characterization of Alkali Metal Fluorides 44
8.1. Sodium Fluoride 44
8.2. Sodium Fluoride (Decagram Scale) 46
8.3. Lithium Fluoride 48
8.4. Potassium Fluoride 50
9. References 52

1. Materials and Methods

Unless otherwise stated, all reagents were purchased from commercial suppliers (abcr, Biosynth, Apollo Scientific, Honeywell, Fluorochem, Merck, Santa Cruz Biotechnology, Strem Chemicals, TCI, Thermo Fisher Scientific, and VWR), used without further purification and stored under ambient conditions.

Acid grade Fluorspar (AGF) was purchased from Mistral Industrial Chemicals (UK), sourced from Minersa group (Asturias region, Spain), contained CaF_2 (>97%), total carbonates (<1.50%), SiO_2 (<1.00%), $BaSO_4$ (<0.50 %), Pb (<0.10%), Fe_2O_3 (<0.10%), S (<0.15%), H_2O (<1.0%), was used without drying and stored under ambient conditions. Lithium hydroxide (LiOH, 98%, anhydrous, Thermo Scientific, CAS 1310-65-2), sodium hydroxide (NaOH, ≥98%, anhydrous pellets, Honeywell, CAS 1310-73-2), potassium hydroxide (KOH, ≥85%, pellets, Sigma Aldrich, CAS 1310-58-3), lithium oxide (Li_2O, ≥98%, Fluorochem, CAS 12057-24-8), titanium dioxide (TiO_2, ≥99.5%, Sigma Aldrich, CAS 13463-67-7), and potassium titanium oxide (K_2TiO_3, abcr, CAS 12030-97-6) were used without drying and stored under ambient conditions. Sodium oxide (Na_2O, 80%, Sigma Aldrich, CAS 1313-59-3) was used without drying and stored in a desiccator.

Solvents (including deuterated solvents for NMR spectroscopy) were purchased from commercial suppliers and used as provided without further purification.

Ball milling was carried out using either a Retsch MM 400 mixer mill (experiments up to 30 Hz), Retsch MM 500 Vario mixer mill (experiments up to 35 Hz) or an Insolido IST636 mixer mill (experiments up to 35 Hz). Unless otherwise stated, mechanochemical reactions were carried out in 15 mL FormTech Scientific (FTS) stainless steel jars with hardened chrome grade 100 steel ball bearings (4 g (10 mm), 7 g (12 mm), 16 g (16 mm)). O-ring inserts (15 mL stainless-steel jars) supplied by RS Components are made of nitrile rubber (NBR). No precaution was taken to exclude air and moisture. Reactions were performed for the time specified without pause.

Planetary ball milling experiments were carried out using a Fritsch Planetary MicroMill 7 “Pulverisette” using 12 mL zirconium oxide grinding bowls containing zirconium oxide grinding balls (0.4 g (5 mm), 1.2 g (7.5 mm), 3.0 g (10 mm), 5.8 g (12.5 mm)). Reactions were performed for a multiple of 3 h cycles (as stated) with pauses in between.

Solution based reactions were stirred using an IKA heating plate, aluminum block and borosilicate glass vials or microwave glass vials.

^{1}H NMR and ^{19}F NMR spectra were recorded on a Bruker Avance III HD nanobay NMR equipped with a 9.4 T magnet (^{1}H 400.3 MHz, ^{19}F 376.6 MHz), a Bruker Neo nanobay NMR equipped with a 9.4 T magnet (^{1}H 400.1 MHz, ^{19}F 376.5 MHz), a Bruker Avance III HD NMR equipped with a 11.75 T magnet (^{1}H 500.3 MHz, ^{19}F 470.8 MHz), or a Bruker Avance III HD NMR equipped with a 11.75 T magnet (^{1}H 500.0 MHz, ^{19}F 470.5 MHz). ^{1}H NMR and ^{19}F NMR spectral data are reported as chemical shifts (δ) in parts per million (ppm) relative to the solvent peak using the Bruker internal referencing procedure (edlock). Coupling constants, *J*, are reported in Hz to the nearest 0.1 Hz. Data are reported as follows: chemical shift, multiplicity (s = singlet, d = doublet, t = triplet, q = quartet, pent = pentet, hept = heptet, br = broad, m = multiplet), coupling constants (Hz) and integration. NMR (nuclear magnetic resonance) spectra were

processed with MestReNova 14.1.2 or Topspin 3.5 or 4.0. Quantitative NMR analysis was determined using sodium triflate as an internal standard. The standard was added to the crude reaction mixture, extracted with D_2O (10% in H_2O), centrifugated and the supernatant was analyzed by NMR spectroscopy.

Solid-state NMR spectra were recorded on a Bruker AVIII 400 spectrometer (^{19}F 376.2 MHz) equipped with a HFXY 3.2 mm probe. Spectra were collected at 298 K and were acquired spinning at the magic angle θ = 54.7356°. Values of magic angle spinning speeds, v_{rot}, of 17 kHz, and 20 kHz to resolve isotropic resonances from spinning side bands. Relevant pulse sequence details are given below each spectrum. Spectra are reported in chemical shift, δ, in parts per million, ppm, relative to $CFCl_3$, δ = 0.0, and were referenced against PTFE, δ = −122.7.[1] Samples were packed into 3.2 mm ZrO_2 rotors fitted with Kel-F drive caps. SS NMR spectra were processed with Topspin 3.6.2. SS NMR peaks were deconvoluted using OriginPro 2023 version 10.0.0.154, using the Peak Deconvolution app available on OriginLab. Peaks were modelled as hybrid Lorentzian/Gaussian functions.

Elemental analyses were carried out by Mikroanalytisches Laboratorium Kolbe. For the determination of Li, Na, K, Ti, and Fe, samples were digested using a MARS 6 Microwave Digestion System (CEM) and analyzed using a SPECTRO ARCOS ICP-OES analyzer (SPECTRO). For the determination of F, samples were thermally digested and combusted using an AQF-2100H Pyrohydrolytic Combustion System (Mitsubishi) and analyzed by ion-chromatography using a 930 Compact IC Flex Oven/SeS/PP/Deg (Metrohm).

Reaction progress and product distributions were monitored using powder X-ray diffraction (PXRD) data collected using a Bruker D8 Advance X-ray diffractometer (Bragg-Brentano geometry; Cu $K\alpha_{1,2}$ radiation). The Bruker D8 Advance X-ray diffractometer was operated with an accelerating voltage of 40 kV and a current of 25 mA. Diffractograms were collected between 5–70° 2θ with 5678 total steps (measurement step size = 0.0114°) and a total effective acquisition time of 596.6 s (0.1051 seconds per step) or 2983 s (0.5254 seconds per step). The phases present in powder samples were identified by comparison against simulated powder diffraction patterns (PDF numbers from ICSD were processed with Mercury 2023.1.0). Patters were simulated between 2θ = 15° and 70°, using wavelength = 1.54056 Å, and peak FWHM = 0.1°. Structure references: TiO_2 (rutile)[2]; TiO_2 (anatase)[2]; CaF_2[3]; $CaTiO_3$[4]; NaF[5]; LiF[6]; NaOH[7]; $Ca(OH)_2$[8]; $KCaF_3$[9]; KF[10]; CaO[11]; $NaVO_3$[12]; Na_2WO_4[13]; $Na_2WO_4 \cdot 2(H_2O)$[14]; Na_2TiO_3[15]; $NaAlO_2$[16].

Thermogravimetric Analysis (TGA) was performed on a TA Discovery TGA 5500 instrument. Powder samples were loaded on a platinum high temperature pan. The samples were heated from 40 to 200 °C (or 750 °C) at a rate of 20 $°C \cdot min^{-1}$ under N_2 flow (25 $mL \cdot min^{-1}$).

2. Optimization Studies on the Solid-State Activation of AGF

2.1. Screening of Sodium Salts as Activators

To a 15 mL stainless steel milling jar was added two hardened chrome steel bearings (2 × 7 g), AGF (1 equiv.) and Na-activator (1.0 equiv. Na^+ per F^-) to give approximately 1 g of material for milling. The jar was then closed and securely fitted to the mill which was set for 3 h at the frequency of 35 Hz. Upon completion, the jar was opened, the powder was collected, and analyzed by PXRD as well as quantitative solid-state NMR spectroscopy. An aliquot of the obtained mixture (approximately 50 mg) and sodium triflate (approximately 10 mg) as internal standard was extracted with D_2O (10 atom% D), centrifugated, and the supernatant was analyzed by quantitative ^{19}F NMR spectroscopy.

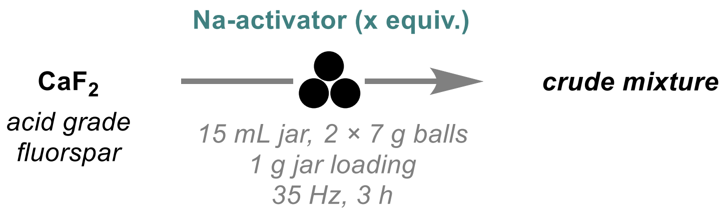

Table S1. Screening of sodium salts as activators for acid grade fluorspar.

Entry	Na-activator (x equiv.)	ΔU_{POT}^{BHFC} [kJ·mol^{-1}][a]	F^- release[b]	CaF_2 conv.[c]	PXRD (major species)
1	Na_2O (1.0 equiv.)	+132	9%	quant.	NaF, CaO
2	Na_2S (1.0 equiv.)	+99[d]	10%	96%	NaF, CaS, *CaF_2 (trace)*
3	Na_3PO_4 (0.67 equiv.)	+97[e]	33%	75%	NaF, $Na_2Ca(PO_4)F$, *CaF_2*
4	NaOH (2.0 equiv.)	+62	8%	88%	NaF, $Ca(OH)_2$, *CaF_2 (trace)*
5	Na_2CO_3 (1.0 equiv.)	+4	7%	13%	NaF, *CaF_2*
6	NaCl (2.0 equiv.)	−100	<1%	<1%	*NaCl*, *CaF_2*
7	Na_2SO_4 (1.0 equiv.)	−249	1%	<1%	$CaSO_4$ (trace), *CaF_2*, *Na_2SO_4*

[a] Changes in lattice energies for reactions (CaF_2 + (2/a) $Na_aX \rightarrow CaX_{(2/a)}$ + 2 NaF) were calculated based on values from reference.[17]
[b] Yields were determined by quantitative ^{19}F NMR spectroscopy in D_2O (10 atom% D) using sodium triflate as an internal standard.
[c] CaF_2 consumption was determined by quantitative solid-state ^{19}F NMR spectroscopy. [d] U_{POT}^{BHFC}(CaS) was taken from reference.[18]
[e] U_{POT}^{BHFC}(Na_3PO_4) was taken from reference.[19]

2.2. Reversibility of Hydroxide-mediated Activation of AGF

To a 15 mL stainless steel milling jar was added two hardened chrome steel bearings (2 × 7 g), AGF (1 equiv.) and NaOH (2.0 equiv.) to give approximately 1 g of material for milling. The jar was then closed and securely fitted to the mill which was set for 3 h at the frequency of 35 Hz. Upon completion, the jar was opened, and the powder was collected. An aliquot of the obtained mixture (approximately 800 mg) was stirred with H_2O (10 mL) at r.t. overnight, centrifugated, and the supernatant was discarded. The insoluble fraction was dried under a flow of N_2 and analyzed by PXRD (Figure S1).

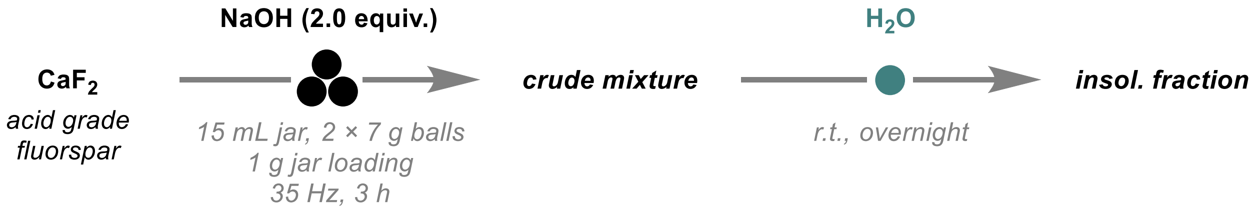

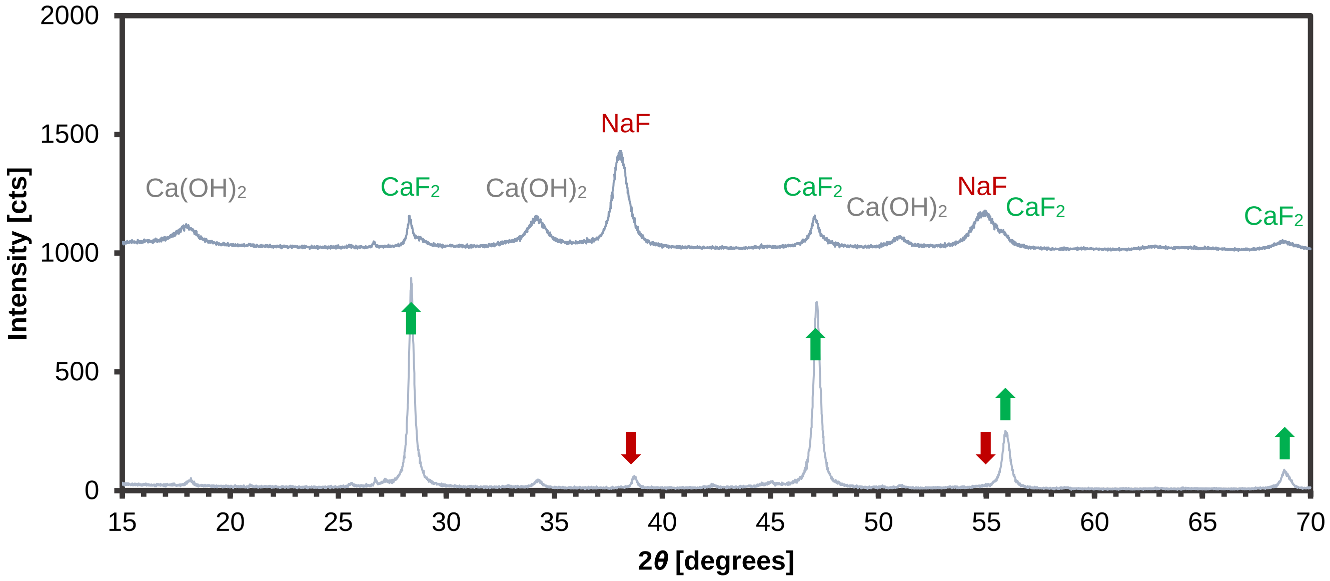

Figure S1. PXRD traces of crude mixture before extraction (*top*), and the insoluble fraction recovered after aqueous wash (*bottom*). PDF# $Ca(OH)_2$: 01-076-0571; CaF_2: 01-077-2093; NaF: 01-075-0448.

2.3. Screening of Sequestrants

To a 15 mL stainless steel milling jar was added two hardened chrome steel bearings (2 × 7 g), AGF (1 equiv.), NaOH (2.0 equiv.), and sequestrant (1.0 equiv.) to give approximately 1 g of material for milling. The jar was then closed and securely fitted to the mill which was set for 3 h at the frequency of 35 Hz. Upon completion, the jar was opened, the powder was collected, and analyzed by PXRD. An aliquot of the obtained mixture (approximately 50 mg) and sodium triflate (approximately 10 mg) as internal standard was extracted with D_2O (10 atom% D), centrifugated, and the supernatant was analyzed by quantitative ^{19}F NMR spectroscopy.

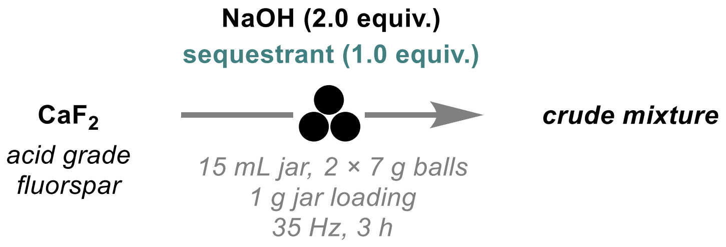

Table S2. Screening of sequestrants in the context of ternary mixtures (CaF_2, NaOH, M_aO_b).

Entry	Sequestrant	F^- release[a]	PXRD (major species)
1	Al_2O_3	8%	NaF, $Ca(OH)_2$, $NaAlO_2$, *CaF_2*
2	SiO_2 (silica gel)	29%	NaF, *CaF_2*
3	SiO_2 (calcined)	32%	NaF, *CaF_2, SiO_2*
4	TiO_2	81%	NaF, $CaTiO_3$, *CaF_2 (trace)*, *TiO_2 (trace)*
5[b]	TiO_2	79%	NaF, $CaTiO_3$
6	V_2O_5	<1%	$NaVO_3$, *CaF_2*
7	ZrO_2	14%	NaF, *ZrO_2*
8	HfO_2	17%	NaF, *HfO_2*
9	WO_3	9%	Na_2WO_4, $Na_2WO_4 \cdot 2H_2O$, *CaF_2*

[a] Yields were determined by quantitative ^{19}F NMR spectroscopy in D_2O (10 atom% D) using sodium triflate as an internal standard.
[b] 1.0 equiv. Na_2O instead of 2.0 equiv. NaOH.

To a 15 mL stainless steel milling jar was added two hardened chrome steel bearings (2 × 7 g), AGF (1 equiv.), and sodium metallate (1.0 equiv. Na^+ per F^-) to give approximately 1 g of material for milling. The jar was then closed and securely fitted to the mill which was set for 3 h at the frequency of 35 Hz. Upon completion, the jar was opened, the powder was collected, and analyzed by PXRD. An aliquot of the obtained mixture (approximately 50 mg) and sodium triflate (approximately 10 mg) as internal standard was extracted with D_2O (10 atom% D), centrifugated, and the supernatant was analyzed by quantitative ^{19}F NMR spectroscopy.

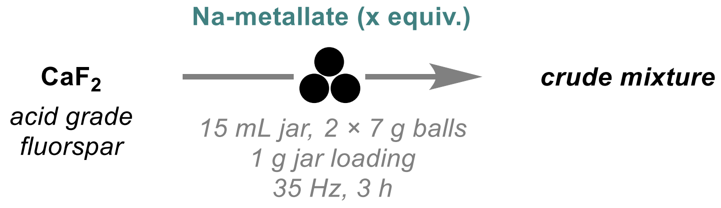

Table S3. Suitability of sodium metallates as activators for AGF.

Entry	Na-metallate (x equiv.)[a]	F^- release[b]	PXRD (major species)
1	$NaAlO_2$ (2.0 equiv.)	5%	NaF (trace), *CaF_2*, *$NaAlO_2$*
2	Na_2SiO_3 (1.0 equiv.)	21%	NaF
3	Na_2TiO_3 (1.0 equiv.)	76%	NaF, $CaTiO_3$
4	$NaVO_3$ (2.0 equiv.)	<1%	*CaF_2*, *$NaVO_3$*
5	Na_2WO_4 (1.0 equiv.)	22%	$Na_3F(WO_4)$, $CaWO_4$, *CaF_2*, *Na_2WO_4*

[a] Sodium metalates, except for commercially available Na_2SiO_3, were prepared by ball milling equimolar amounts of Na_2O and the corresponding metal oxide (Al_2O_3, V_2O_5, WO_3) at 35 Hz for 3 h (cf. section 3). [b] Yields were determined by quantitative ^{19}F NMR spectroscopy in D_2O (10 atom% D) using sodium triflate as an internal standard.

2.4. Effect of TiO_2 Polymorphs and Particle Size

To a 15 mL stainless steel milling jar was added two hardened chrome steel bearings (2 × 7 g), AGF (1 equiv.), NaOH (2.0 equiv.), and TiO_2 (1.0 equiv., polymorph as stated below) to give approximately 1 g of material for milling. The jar was then closed and securely fitted to the mill which was set for 3 h at the frequency of 35 Hz. Upon completion, the jar was opened, the powder was collected, and analyzed by PXRD. An aliquot of the obtained mixture (approximately 50 mg) and sodium triflate (approximately 10 mg) as internal standard was extracted with D_2O (10 atom% D), centrifugated, and the supernatant was analyzed by quantitative ^{19}F NMR spectroscopy.

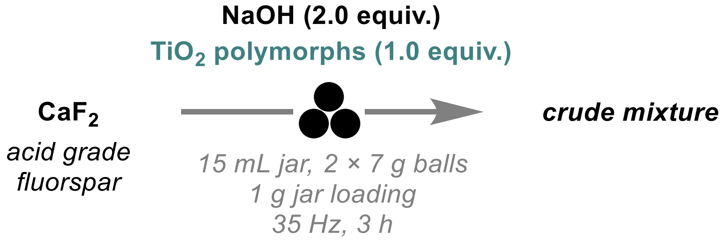

Table S4. Effect of different TiO_2 polymorphs on the activation of AGF.

Entry	Polymorph	Particle Size[a]	F^- release[b]	PXRD (major species)
1	anatase & rutile[c]	21 nm	81%	NaF, $CaTiO_3$, *CaF_2 (trace)*, *TiO_2 (trace)*
2	anatase	32 nm	74%	NaF, $CaTiO_3$, *CaF_2 (trace)*
3	anatase	<44 μm	30%	NaF, $Ca(OH)_2$, *TiO_2*, $CaTiO_3$ (trace), *CaF_2 (trace)*
4	rutile	<100 nm	80%	NaF, $CaTiO_3$, *CaF_2 (trace)*
5	rutile	0.2-0.3 μm	34%	NaF, $CaTiO_3$, $Ca(OH)_2$, *TiO_2*

[a] Particle size as stated by the supplier. [b] Yields were determined by quantitative ^{19}F NMR spectroscopy in D_2O (10 atom% D) using sodium triflate as an internal standard. [c] Commercial sample containing both polymorphs used throughout this study.

2.5. Effect of Water

To a 15 mL stainless steel milling jar was added two hardened chrome steel bearings (2 × 7 g), AGF (1 equiv.), NaOH (2.0 equiv.), TiO_2 (1.0 equiv.), and H_2O (equiv. as stated below) to give approximately 1 g of material for milling. The jar was then closed and securely fitted to the mill which was set for 3 h at the frequency of 35 Hz. Upon completion, the jar was opened, the powder was collected. An aliquot of the obtained mixture (approximately 50 mg) and sodium triflate (approximately 10 mg) as internal standard was extracted with D_2O (10 atom% D), centrifugated, and the supernatant was analyzed by quantitative ^{19}F NMR spectroscopy.

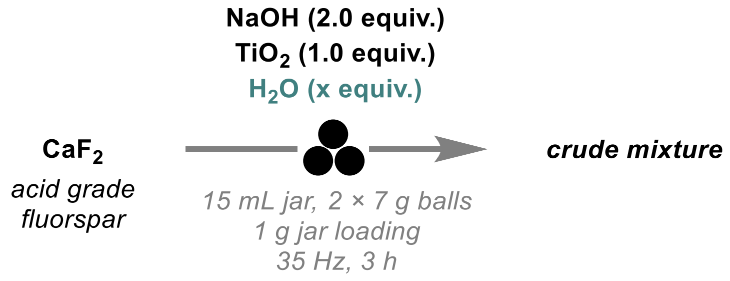

Table S5. Effect of water on the activation of AGF.

Entry	equiv. H_2O	F^- release[a]
1	-	81%
2	0.5	79%
3	1.0	70%
4	2.0	22%

[a] Yields were determined by quantitative ^{19}F NMR spectroscopy in D_2O (10 atom% D) using sodium triflate as an internal standard.

2.6. Control Experiments (Selectivity/Compatibility of Components)

To a 15 mL stainless steel milling jar was added two hardened chrome steel bearings (2 × 7 g), and approximately 1 g of binary mixtures (as specified in Table S6). The jar was then closed and securely fitted to the mill which was set for 3 h at the frequency of 35 Hz. Upon completion, the jar was opened, the powder was collected, and analyzed by PXRD. An aliquot of the obtained mixture (approximately 50 mg) and sodium triflate (approximately 10 mg) as internal standard was extracted with D_2O (10 atom% D), centrifugated, and the supernatant was analyzed by quantitative ^{19}F NMR spectroscopy.

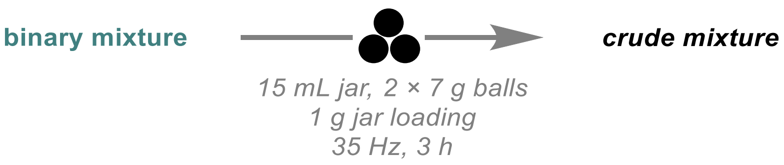

Table S6. Control experiment to demonstrate the compatibilty of the reaction components.

Entry	binary mixture	F^- recovery[a]	PXRD (major species)
1	1.0 equiv. CaF_2 (AGF) + 1.0 equiv. NaF	89%	*NaF*, *CaF_2*
2	1.0 equiv. $Ca(OH)_2$ + 1.0 equiv. TiO_2	n.a.	$CaTiO_3$, *TiO_2 (trace)*
3	1.0 equiv. $CaTiO_3$ + 2.0 equiv. NaF	77%	*NaF*, *$CaTiO_3$*
4	1.0 equiv. TiO_2 + 2.0 equiv. NaF	quant.	*NaF*, *TiO_2*
5	1.0 equiv. TiO_2 + 2.0 equiv. NaOH	n.a.	Na_2TiO_3

[a] Yields were determined by quantitative ^{19}F NMR spectroscopy in D_2O (10 atom% D) using sodium triflate as an internal standard; n.a. = not applicable.

To a 15 mL stainless steel milling jar was added two hardened chrome steel bearings (2 × 7 g), $CaTiO_3$ or $CaSiO_3$ (1 equiv.), and NaF (2.0 equiv.) to give approximately 1 g of material for milling. The jar was then closed and securely fitted to the mill which was set for 3 h at the frequency of 35 Hz. Upon completion, the jar was opened, the powder was collected, and analyzed by PXRD.

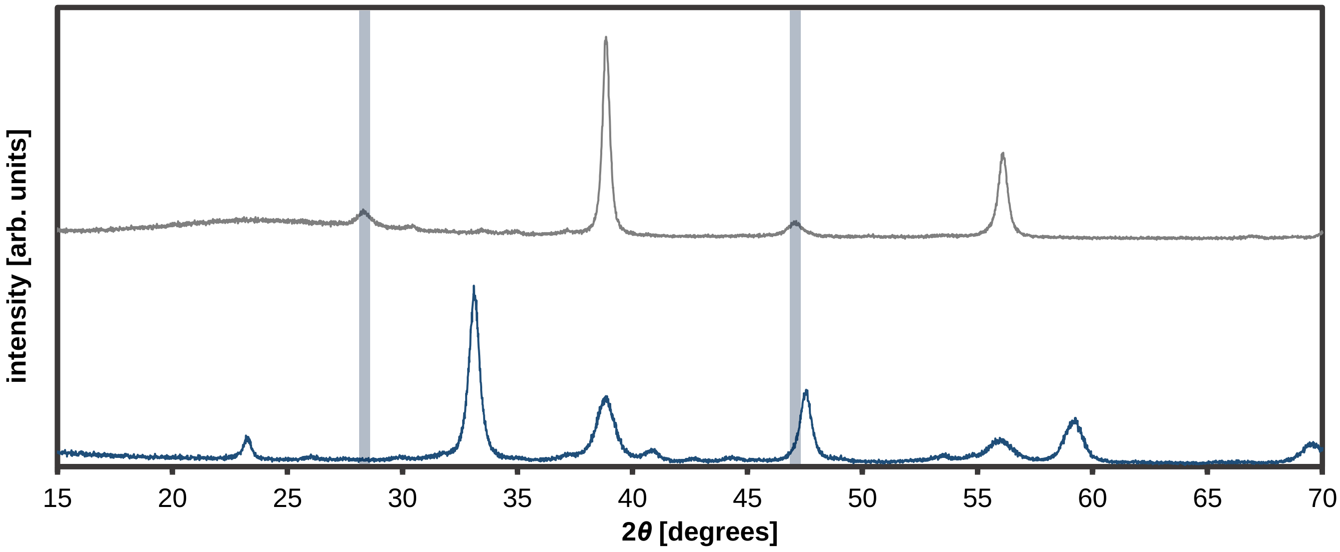

Figure S2. PXRD traces of $CaTiO_3$ milled with 2 equiv. NaF (*top*) and $CaSiO_3$ with 2 equiv. NaF (*bottom*); both at 35 Hz for 3 h. Diagnostic reflections of CaF_2 are highlighted in blue. PDF# CaF_2: 01-077-2093; NaF: 01-075-0448; $CaTiO_3$: 01-078-1013.

2.7. Optimization of Mechanochemical Parameters

2.7.1. Ball Bearings

To a 15 mL stainless steel milling jar was added hardened chrome steel bearings (as specified in Table S7), AGF (1 equiv.), NaOH (2.0 equiv.), and TiO_2 (1.0 equiv.) to give approximately 1 g of material for milling. The jar was then closed and securely fitted to the mill which was set for 3 h at the frequency of 35 Hz. Upon completion, the jar was opened, and the powder was collected. An aliquot of the obtained mixture (approximately 50 mg) and sodium triflate (approximately 10 mg) as internal standard was extracted with D_2O (10 atom% D), centrifugated, and the supernatant was analyzed by quantitative ^{19}F NMR spectroscopy.

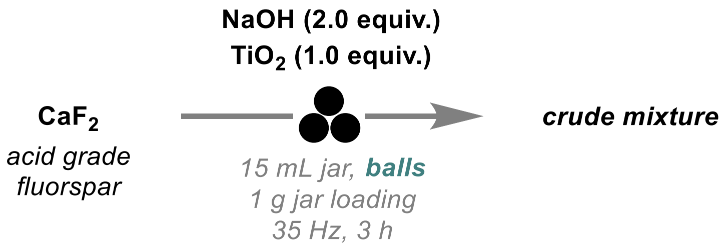

Table S7. Effect of number and mass of ball bearings on the fluoride release.

Entry	number of bearings	bearing mass [g]	total mass of bearings [g]	F^- release[a]
1	1	7.0	7.0	49%
2	2	7.0	14.0	81%
3	3	7.0	21.0	79%
4	2	4.0	8.0	76%
5	4	4.0	12.0	82%[b]
6	6	4.0	16.0	86%[b]

[a] Yields were determined by quantitative ^{19}F NMR spectroscopy in D_2O (10 atom% D) using sodium triflate as an internal standard.
[b] More difficult recovery of the powdered mixture after milling due to the higher number of ball bearings.

2.7.2. Jar Loading

To a 15 mL stainless steel milling jar was added two hardened chrome steel bearings (2 × 7 g), AGF (1 equiv.), NaOH (2.0 equiv.), and TiO_2 (1.0 equiv.) to give the amount of material for milling as specified in Table S8. The jar was then closed and securely fitted to the mill which was set for 3 h at the frequency of 35 Hz. Upon completion, the jar was opened, and the powder was collected. An aliquot of the obtained mixture (approximately 50 mg) and sodium triflate (approximately 10 mg) as internal standard was extracted with D_2O (10 atom% D), centrifugated, and the supernatant was analyzed by quantitative ^{19}F NMR spectroscopy.

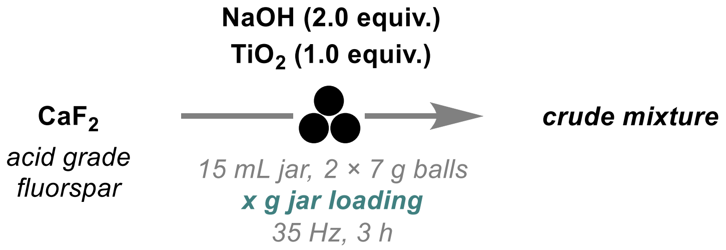

Table S8. Effect of jar loading on the fluoride release.

Entry	Jar loading [g]	F^- release[a]
1	0.50	84%
2	1.00	81%
3	1.50	80%
4	2.00	78%
5	2.50	63%

[a] Yields were determined by quantitative ^{19}F NMR spectroscopy in D_2O (10 atom% D) using sodium triflate as an internal standard.

2.7.3. Milling Frequency

To a 15 mL stainless steel milling jar was added two hardened chrome steel bearings (2 × 7 g), AGF (1 equiv.), NaOH (2.0 equiv.), and TiO_2 (1.0 equiv.) to give approximately 1 g of material for milling. The jar was then closed and securely fitted to the mill which was set for 3 h at the frequency as specified in Table S9. Upon completion, the jar was opened, the powder was collected, and analyzed by PXRD (Figure S3). An aliquot of the obtained mixture (approximately 50 mg) and sodium triflate (approximately 10 mg) as internal standard was extracted with D_2O (10 atom% D), centrifugated, and the supernatant was analyzed by quantitative ^{19}F NMR spectroscopy.

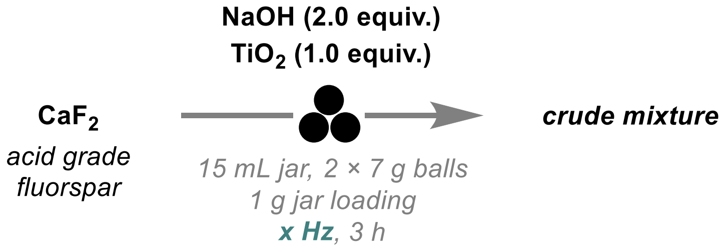

Table S9. Effect of milling frequency on the fluoride release.

Entry	Milling Frequency [Hz]	F^- release[a]
1	15	17%
2	20	45%
3	25	66%
4	30	77%
5	35	81%

[a] Yields were determined by quantitative ^{19}F NMR spectroscopy in D_2O (10 atom% D) using sodium triflate as an internal standard.

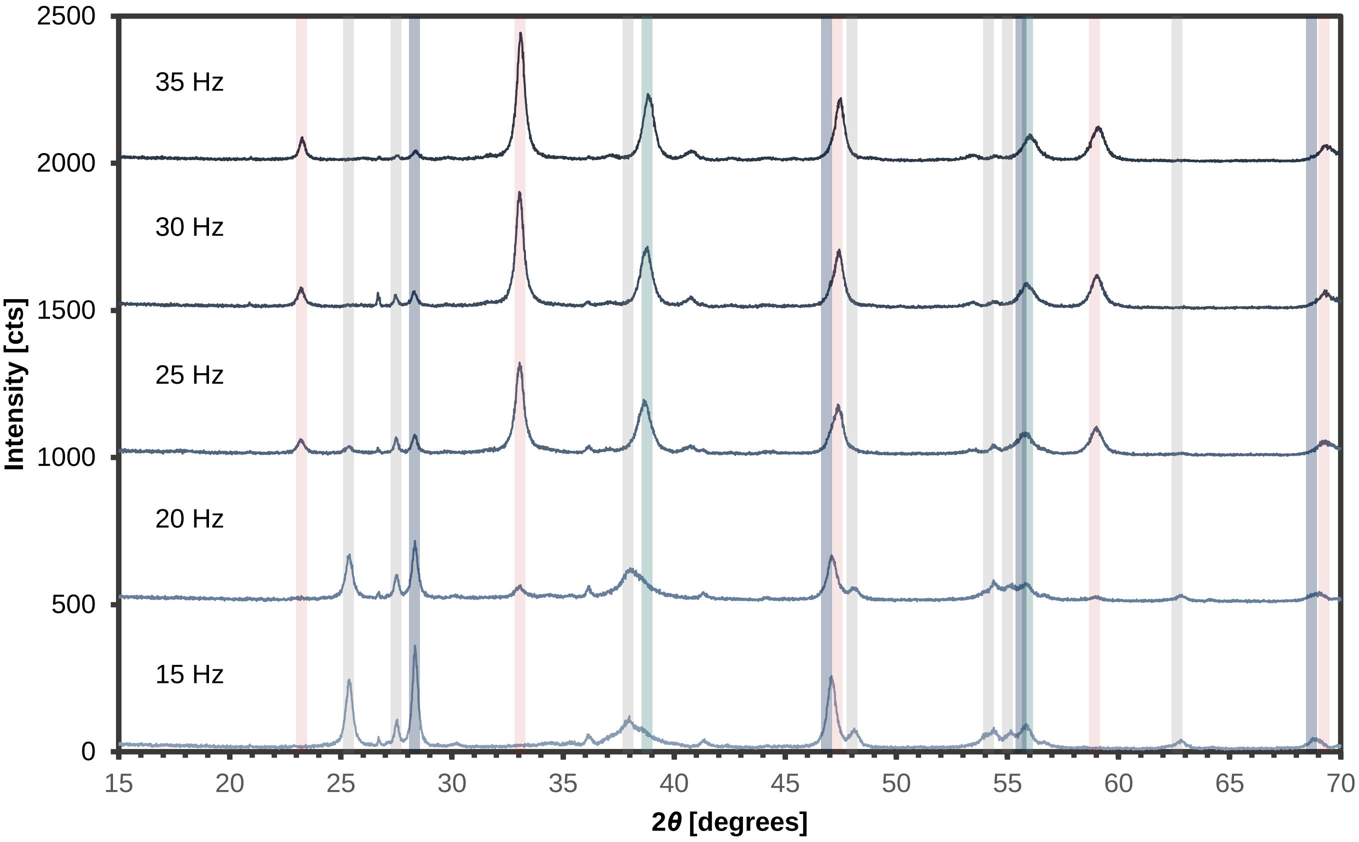

Figure S3. PXRD traces of crude mixtures obtained after milling at various frequencies, with diagnostic reflections highlighted in color (CaF_2 = blue, TiO_2 = grey, NaF = green, $CaTiO_3$ = red). PDF# CaF_2: 01-077-2093; TiO_2 (anatase): 01-089-4203; TiO_2 (rutile): 01-089-4202; NaF: 01-075-0448; $CaTiO_3$: 01-078-1013.

2.7.4. Milling Time and Milling Frequency

To a 15 mL stainless steel milling jar was added two hardened chrome steel bearings (2 × 7 g), AGF (1 equiv.), NaOH (2.0 equiv.), and TiO_2 (1.0 equiv.) to give approximately 1 g of material for milling. The jar was then closed and securely fitted to the mill which was set for the time and frequency as specified in Table S10. Upon completion, the jar was opened, the powder was collected, and analyzed by PXRD (Figure S4). An aliquot of the obtained mixture (approximately 50 mg) and sodium triflate (approximately 10 mg) as internal standard was extracted with D_2O (10 atom% D), centrifugated, and the supernatant was analyzed by quantitative ^{19}F NMR spectroscopy.

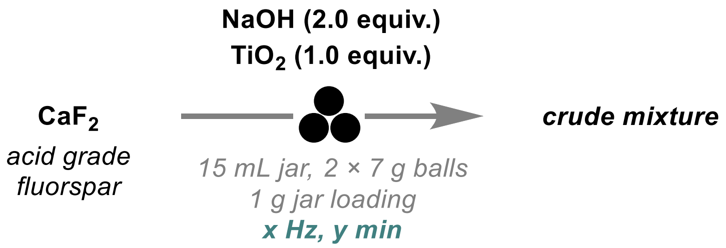

Table S10. Effect of milling time and frequency on the fluoride release.

Entry	Milling Time [min]	F^- release[a] at 25 Hz	F^- release[a] at 30 Hz	F^- release[a] at 35 Hz
1	5	3%	4%	7%
2	10	4%	7%	18%
3	15	7%	13%	24%
4	30	15%	26%	43%
5	60	24%	51%	68%
6	120	53%	71%	74%
7	180	66%	77%	76%
8	360	n.d.	n.d.	74%

[a] Yields were determined by quantitative ^{19}F NMR spectroscopy in D_2O (10 atom% D) using sodium triflate as an internal standard. n.d. = not determined.

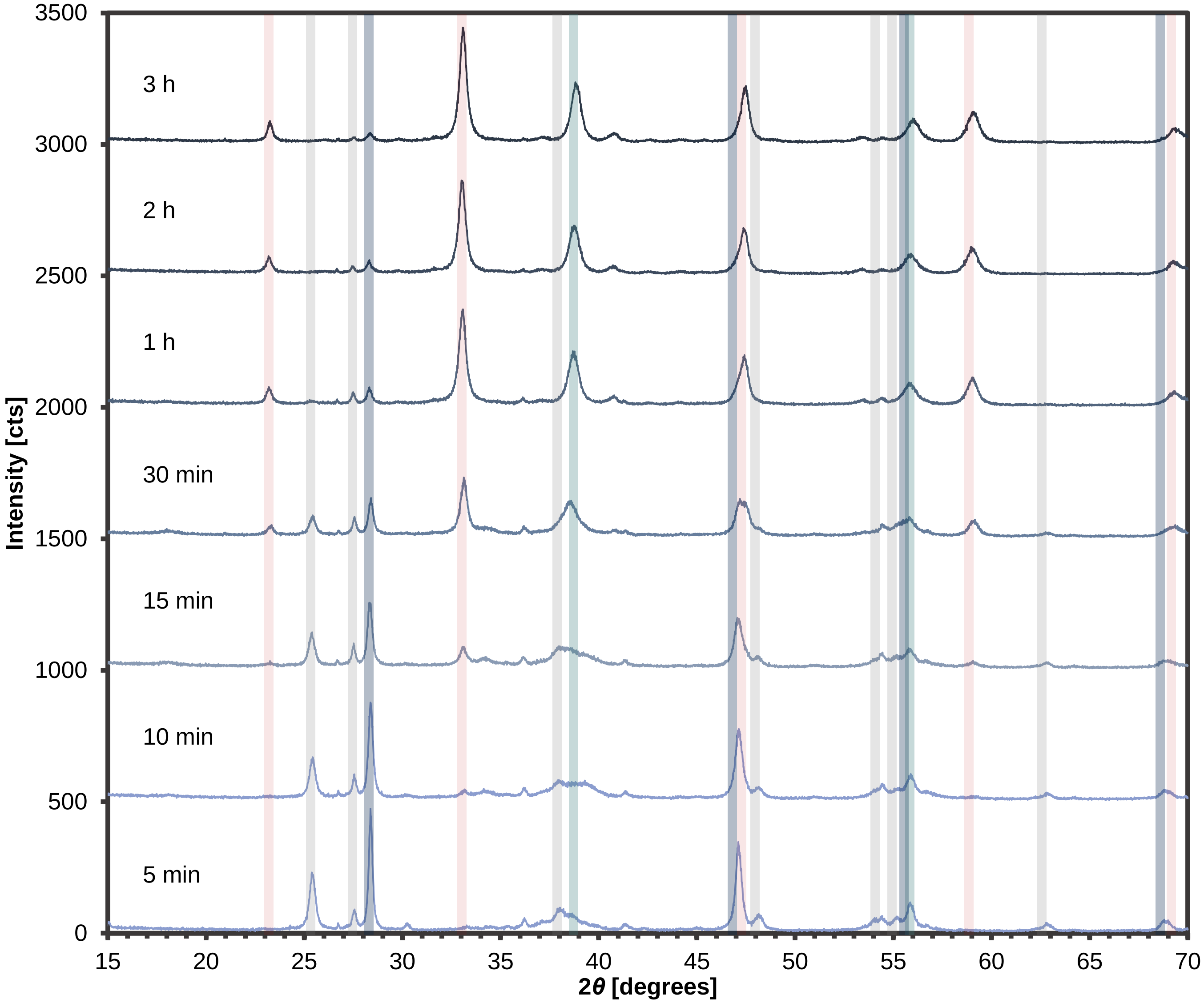

Figure S4. PXRD traces of crude mixtures obtained after milling at 35 Hz for various times, with diagnostic reflections highlighted in color (CaF_2 = blue, TiO_2 = grey, NaF = green, $CaTiO_3$ = red). PDF# CaF_2: 01-077-2093; TiO_2 (anatase): 01-089-4203; TiO_2 (rutile): 01-089-4202; NaF: 01-075-0448; $CaTiO_3$: 01-078-1013.

2.7.5. Optimization of Planetary Ball Milling

To a 12 mL zirconium oxide milling jar was added zirconium oxide bearings (as specified in Table S11), AGF (1 equiv.), NaOH (2.0 equiv.), and TiO_2 (1.0 equiv.) to give approximately 1 g of material for milling. The jar was then closed and securely fitted to the mill which was set for 3 h at the frequency of 800 rpm. Upon completion, the jar was opened, and the powder was collected. An aliquot of the obtained mixture (approximately 50 mg) and sodium triflate (approximately 10 mg) as internal standard was extracted with D_2O (10 atom% D), centrifugated, and the supernatant was analyzed by quantitative ^{19}F NMR spectroscopy.

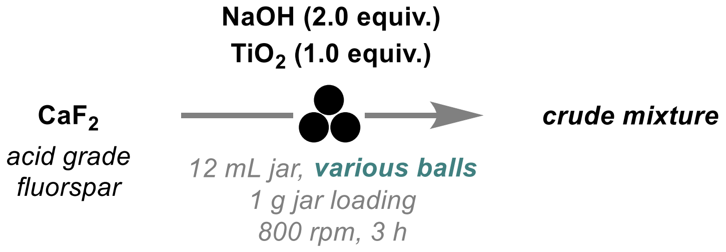

Table S11. Effect of number and mass of ball bearings on the fluoride release (planetary ball milling).

Entry	number of bearings	mass of bearing [g]	total mass of bearings [g]	F^- release[a]
1	6	1.0	6.0	18%
2	10	1.1	11.0	11%
3	2	5.8	11.6	31%
4	4	3.2	12.8	50%
5	6	3.2	19.2	62% (85%)
6	50	0.4	20.0	56% (76%)
7	8	3.2	25.6	22%

[a] Yields were determined by quantitative ^{19}F NMR spectroscopy in D_2O (10 atom% D) using sodium triflate as an internal standard. Yields given in parentheses refer to reactions conducted for 3 × 3 h instead of 3 h.

2.8. Optimization of Activator Stoichiometry

To a 15 mL stainless steel milling jar was added two hardened chrome steel bearings (2 × 7 g), AGF (1 equiv.), NaOH (as specified in Table S12), and TiO_2 (1.0 equiv.) to give approximately 1 g of material for milling. The jar was then closed and securely fitted to the mill which was set for 3 h at 35 Hz. Upon completion, the jar was opened, and the powder was collected. An aliquot of the obtained mixture (approximately 50 mg) and sodium triflate (approximately 10 mg) as internal standard was extracted with D_2O (10 atom% D), centrifugated, and the supernatant was analyzed by quantitative ^{19}F NMR spectroscopy.

Another aliquot of the obtained mixture (approximately 100 mg) was extracted with H_2O (3 × 1 mL), centrifugated, the supernatant was concentrated and flame-dried under vacuo (heat gun) to give crude NaF. All of the obtained NaF (approximately 30 mg) and sodium triflate (approximately 20 mg) as internal standard was dissolved in D_2O (10 atom% D), and analyzed by quantitative ^{19}F NMR spectroscopy.

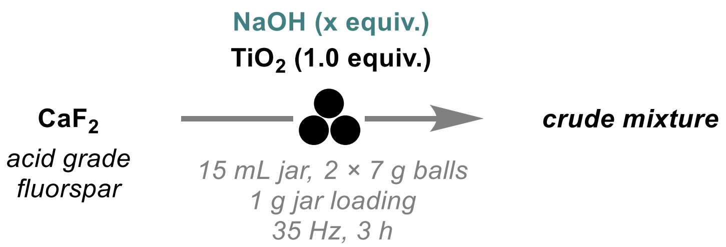

Table S12. Optimization of the amount of NaOH with regard to the yield and purity of NaF.

Entry	equiv. NaOH	F^- release[a]	CaF_2 conv.[a]	NaF purity[b]
1	1.0	62%	31%	n.d.
2	1.6	84%	67%	96%
3	1.8	84%	76%	94%
4	2.0	81%	81%	91%
5	3.0	71%	71%	n.d.

[a] Yields were determined by quantitative ^{19}F NMR spectroscopy in D_2O (10 atom% D) using sodium triflate as an internal standard.
[b] Purity was determined by quantitative ^{19}F NMR spectroscopy in D_2O (10 atom% D) using sodium triflate as an internal standard.
n.d. = not determined.

3. Preparation of Sodium Metallates

3.1. Sodium Aluminate

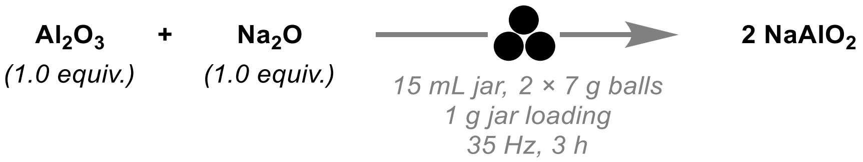

To a 15 mL stainless steel milling jar was added two hardened chrome steel bearings (2 × 7 g), Al_2O_3 (1 equiv.), and Na_2O (1.0 equiv.) to give approximately 1 g of material for milling. The jar was then closed and securely fitted to the mill which was set for 3 h at a frequency of 35 Hz. Upon completion, the jar was opened, the powder was collected, and analyzed by PXRD confirming its identity.

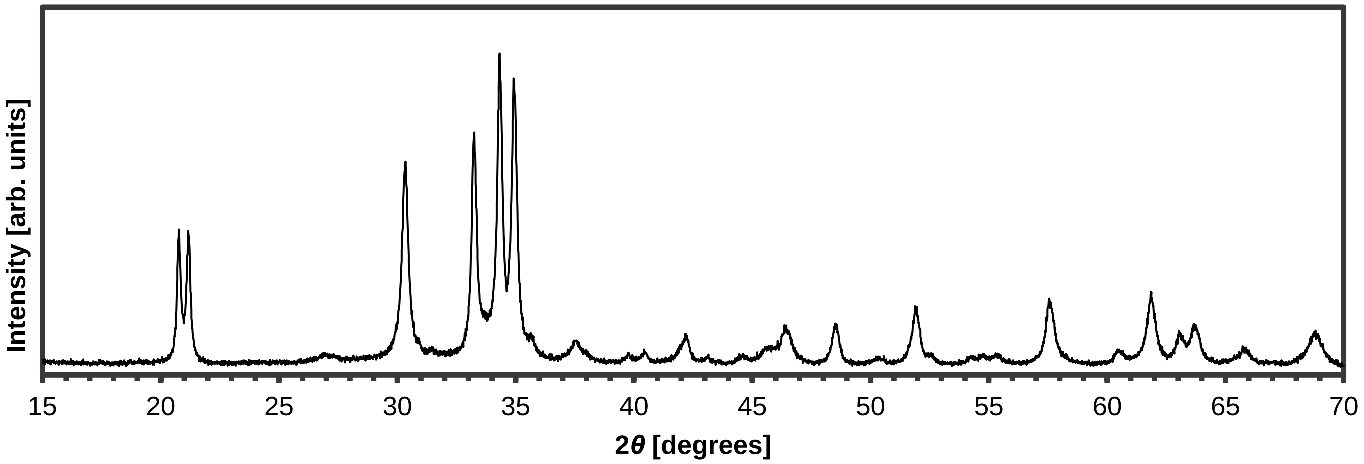

Figure S5. PXRD trace of $NaAlO_2$ as obtained after ball milling equimolar amounts of Al_2O_3 and Na_2O. PDF# $NaAlO_2$: 01-090-0702.

3.2. Sodium Metatitanate

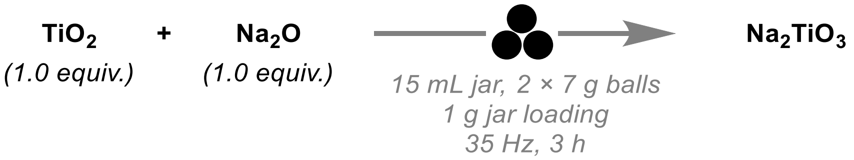

Five 15 mL stainless steel milling jars were charged with two hardened chrome steel bearings (2 × 7 g), TiO_2 (563 mg, 1 equiv.), and Na_2O (437 mg, 1.0 equiv.) each. The jars were then closed and securely fitted to the mill which was set for 3 h at a frequency of 35 Hz. Upon completion, the jar was opened, the powder was collected, and analyzed by PXRD confirming its identity.

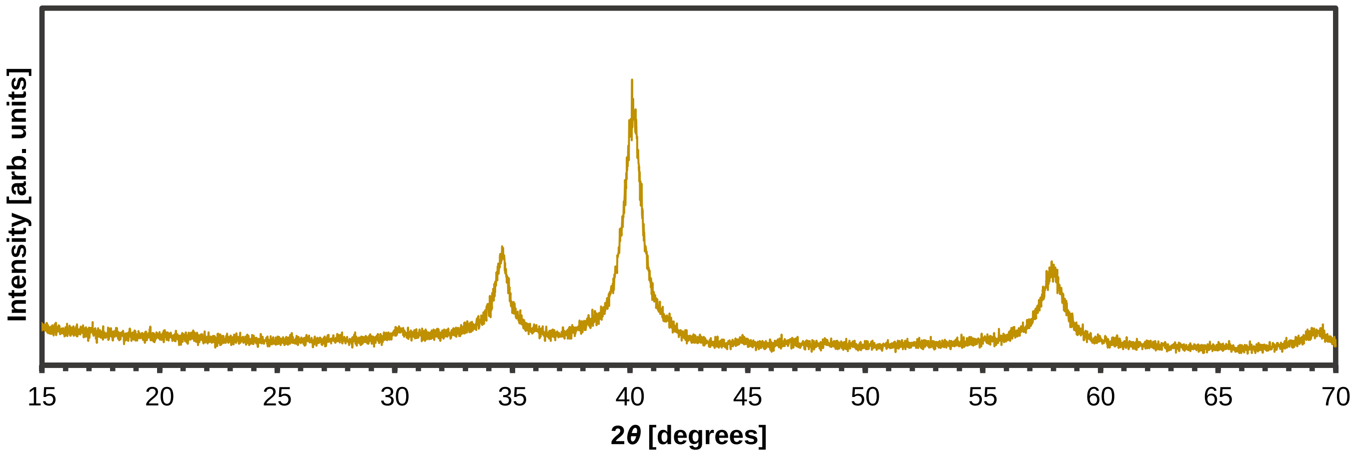

Figure S6. PXRD trace of Na_2TiO_3 as obtained after ball milling equimolar amounts of TiO_2 and Na_2O. PDF# Na_2TiO_3: 01-080-6123.

3.3. Sodium Metavanadate

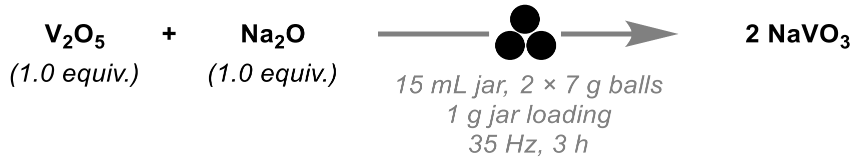

To a 15 mL stainless steel milling jar was added two hardened chrome steel bearings (2 × 7 g), V_2O_5 (1 equiv.), and Na_2O (1.0 equiv.) to give approximately 1 g of material for milling. The jar was then closed and securely fitted to the mill which was set for 3 h at a frequency of 35 Hz. Upon completion, the jar was opened, the powder was collected, and analyzed by PXRD confirming its identity.

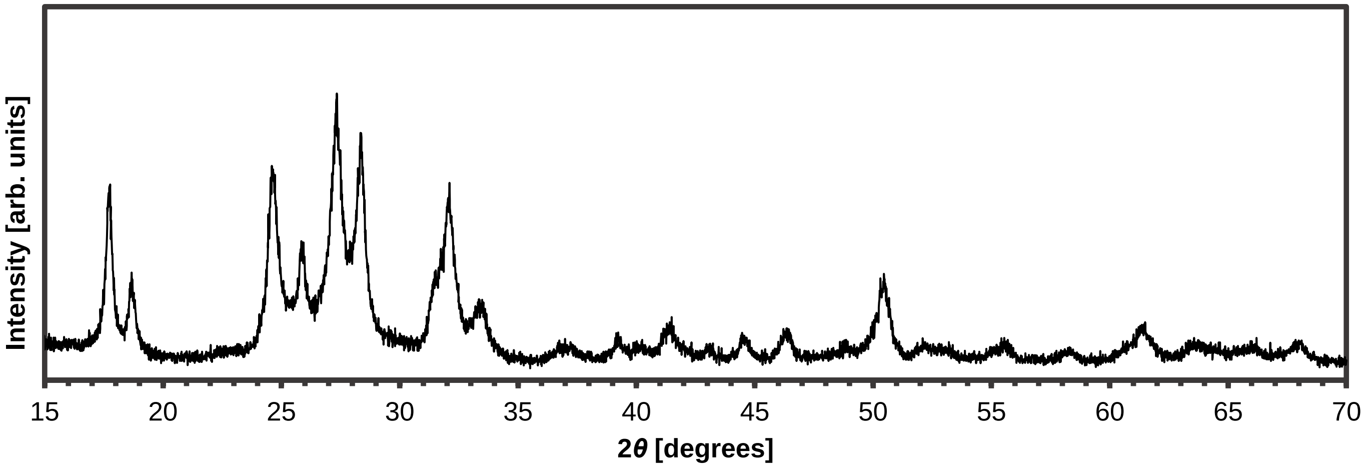

Figure S7. PXRD trace of $NaVO_3$ as obtained after ball milling equimolar amounts of V_2O_5 and Na_2O. PDF# $NaVO_3$: 01-070-1015.

3.4. Sodium Tungstate

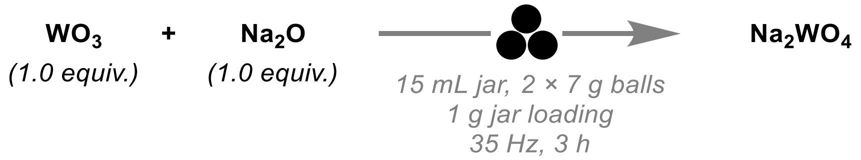

To a 15 mL stainless steel milling jar was added two hardened chrome steel bearings (2 × 7 g), WO_3 (1 equiv.), and Na_2O (1.0 equiv.) to give approximately 1 g of material for milling. The jar was then closed and securely fitted to the mill which was set for 3 h at a frequency of 35 Hz. Upon completion, the jar was opened, the powder was collected, and analyzed by PXRD confirming its identity.

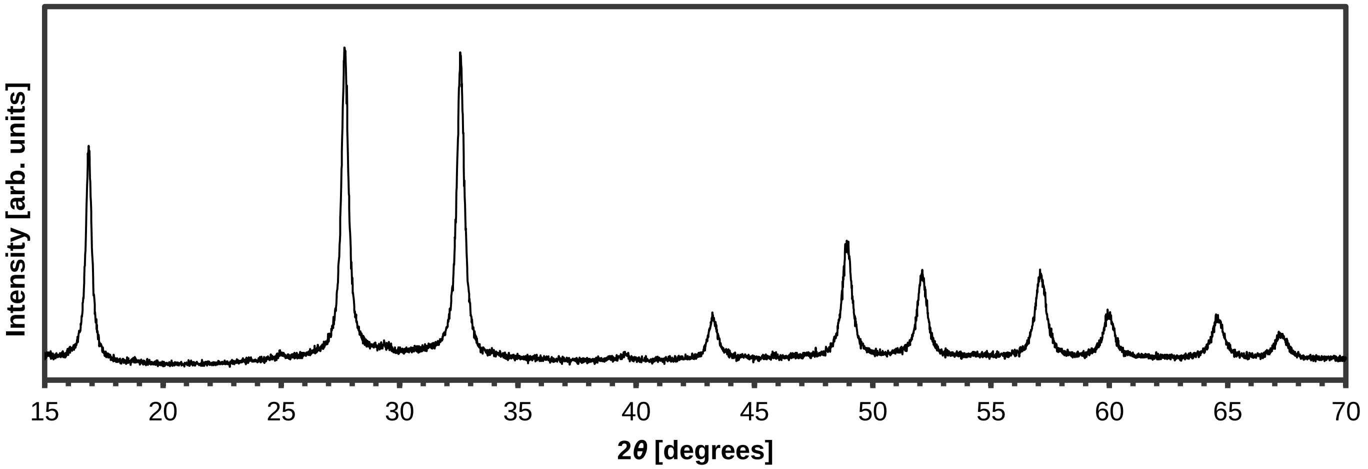

Figure S8. PXRD trace of Na_2WO_4 as obtained after ball milling equimolar amounts of WO_3 and Na_2O. PDF# Na_2WO_4: 01-070-1040.

4. Mechanistic Investigations

4.1. Plausible Mechanistic Pathways and Putative Elementary Steps

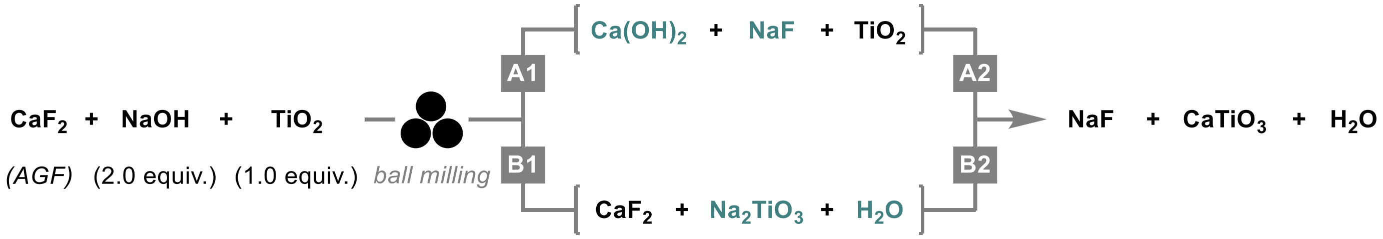

4.2. Pathway A

To a 15 mL stainless steel milling jar was added two hardened chrome steel bearings (2 × 7 g), AGF (494 mg, 1 equiv.), and NaOH (506 mg, 2.0 equiv.). The jar was then closed and securely fitted to the mill which was set for 3 h at the frequency as specified in Figure S9. Upon completion, the jar was opened, the powder was collected, and analyzed by PXRD.

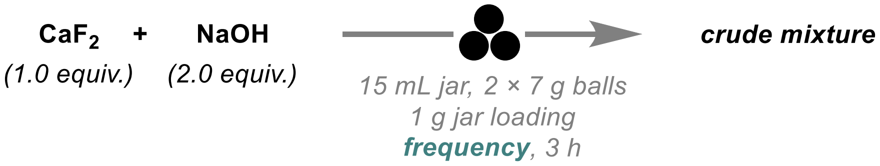

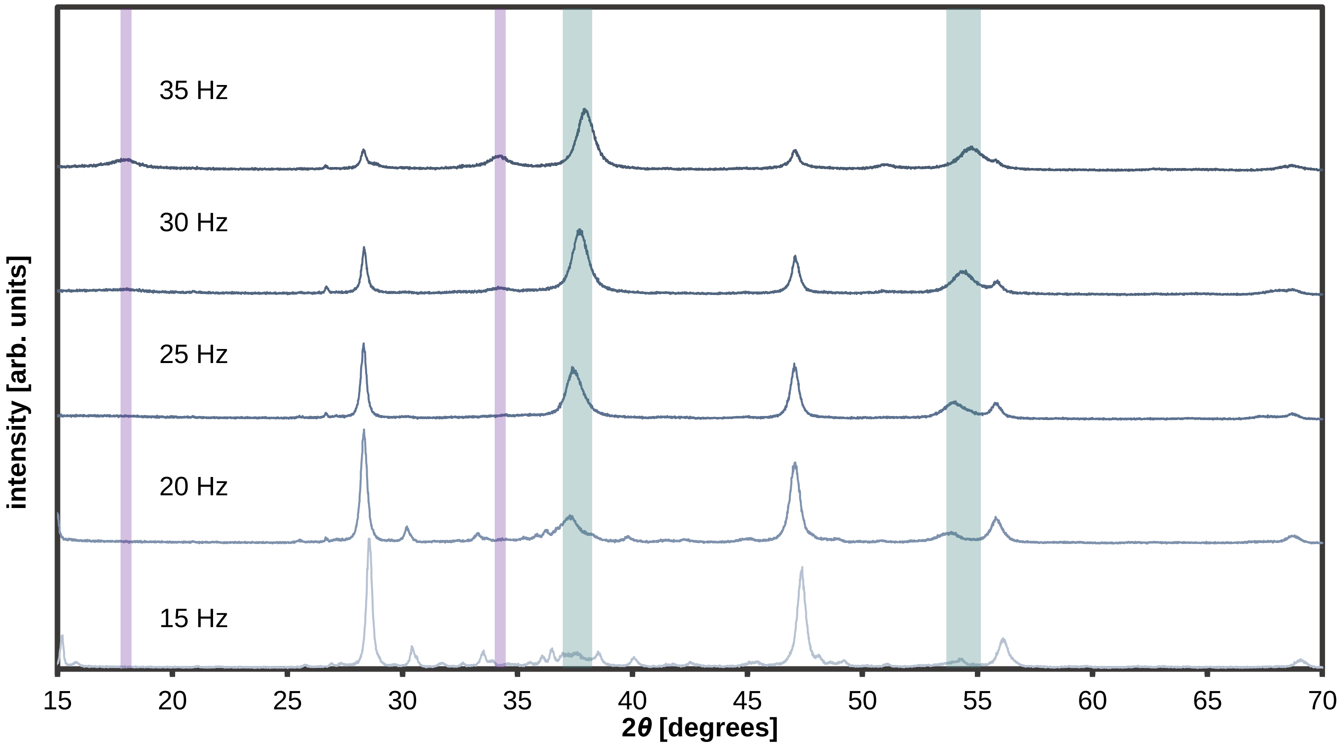

Figure S9. PXRD traces of crude mixtures obtained after milling AGF with NaOH at various frequencies, with diagnostic reflections highlighted in color (NaF = green, $Ca(OH)_2$ = purple). PDF# NaF: 01-075-0448; $Ca(OH)_2$: 01-076-0571; CaF_2: 01-077-2093.

To a 15 mL stainless steel milling jar was added two hardened chrome steel bearings (2 × 7 g), $Ca(OH)_2$ (481 mg, 1 equiv.), and TiO_2 (519 mg, 1.0 equiv.). The jar was then closed and securely fitted to the mill which was set for 3 h at the frequency as specified in Figure S10. Upon completion, the jar was opened, the powder was collected, and analyzed by PXRD.

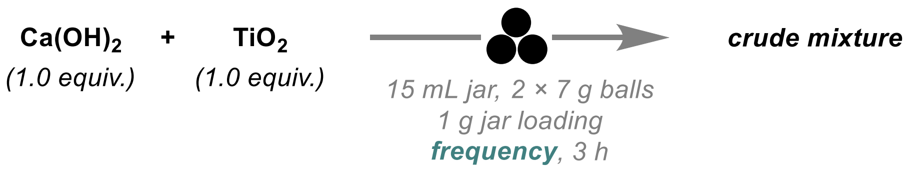

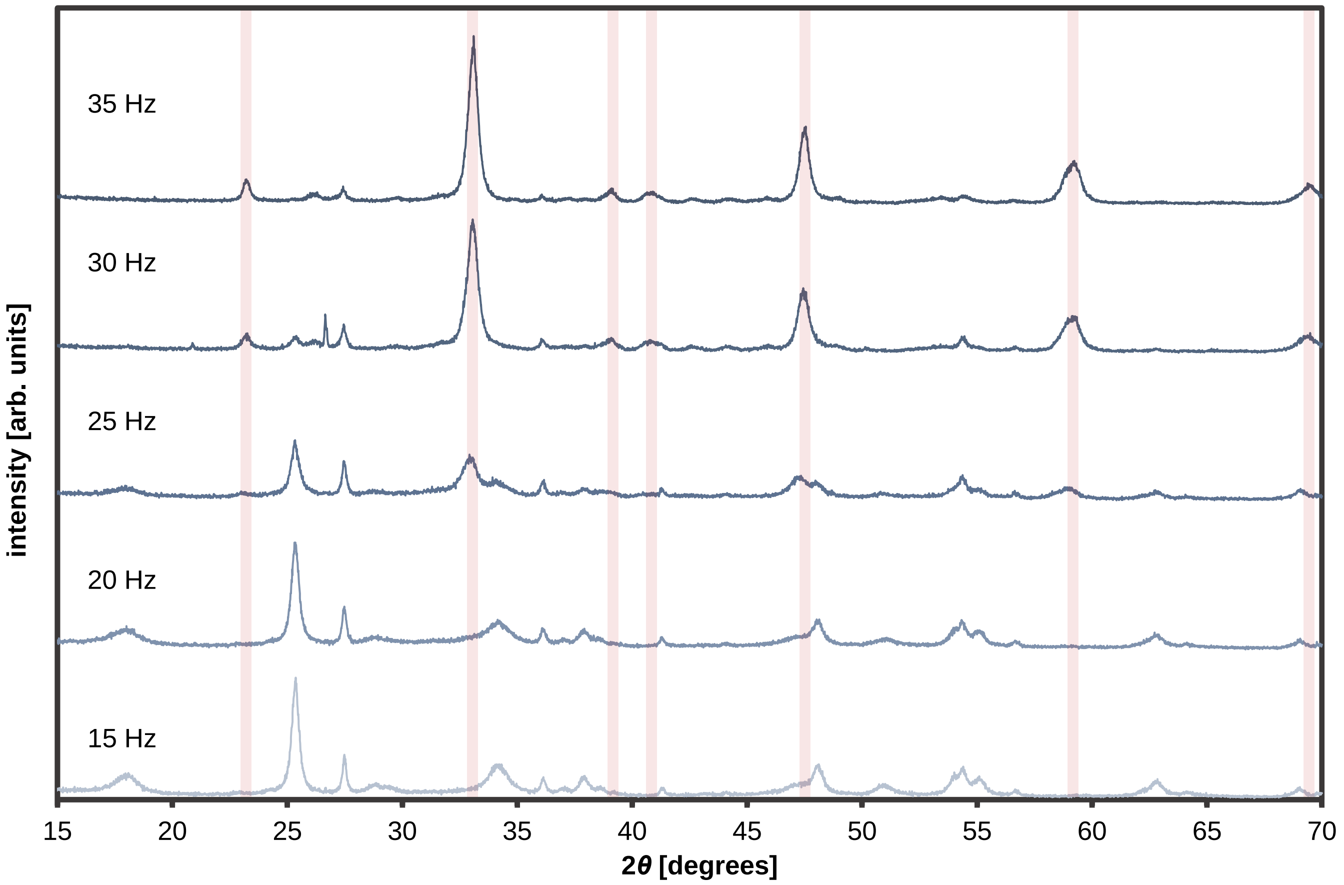

Figure S10. PXRD traces of crude mixtures obtained after milling $Ca(OH)_2$ with TiO_2 at various frequencies, with diagnostic reflections highlighted in color ($CaTiO_3$ = red). PDF# $CaTiO_3$: 01-078-1013; $Ca(OH)_2$: 01-076-0571; TiO_2 (anatase): 01-089-4203; TiO_2 (rutile): 01-089-4202.

4.3. Pathway B

To a 15 mL stainless steel milling jar was added two hardened chrome steel bearings (2 × 7 g), TiO_2 (500 mg, 1 equiv.), and NaOH (500 mg, 2.0 equiv.). The jar was then closed and securely fitted to the mill which was set for 3 h at the frequency as specified in Figure S11. Upon completion, the jar was opened, the powder was collected, and analyzed by PXRD.

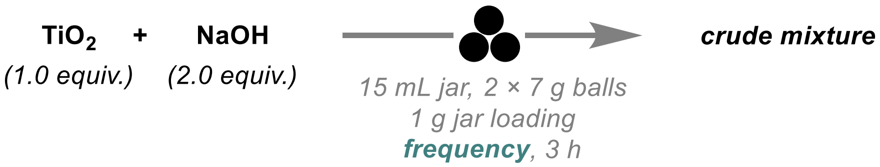

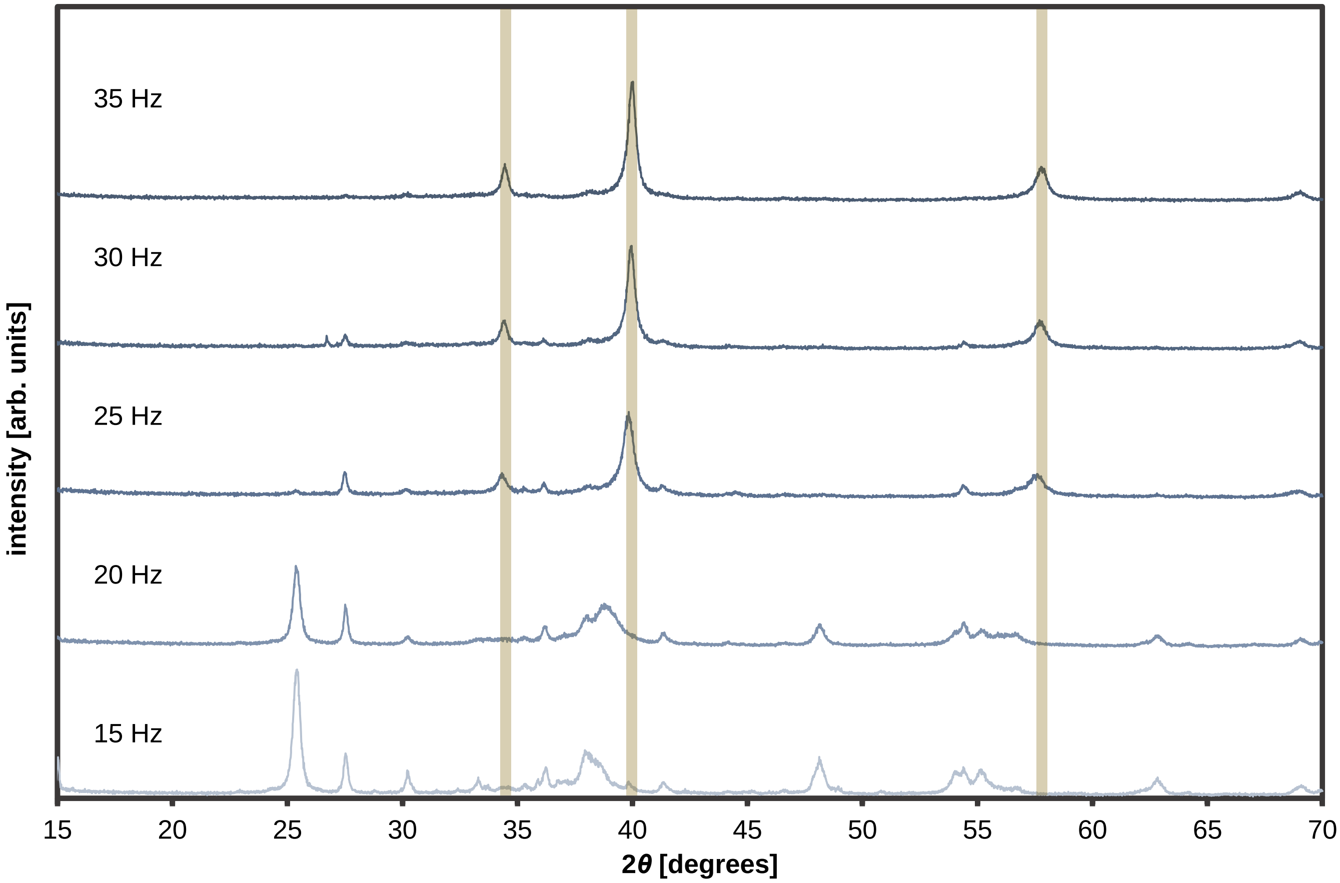

Figure S11. PXRD traces of crude mixtures obtained after milling TiO_2 with NaOH at various frequencies, with diagnostic reflections highlighted in color (Na_2TiO_3 = gold). PDF# Na_2TiO_3: 01-080-6123; TiO_2 (anatase): 01-089-4203; TiO_2 (rutile): 01-089-4202.

To a 15 mL stainless steel milling jar was added two hardened chrome steel bearings (2 × 7 g), AGF (355 mg, 1 equiv.), and Na_2TiO_3 (645 mg, 1.0 equiv.). The jar was then closed and securely fitted to the mill which was set for 3 h at the frequency as specified in Figure S12. Upon completion, the jar was opened, the powder was collected, and analyzed by PXRD.

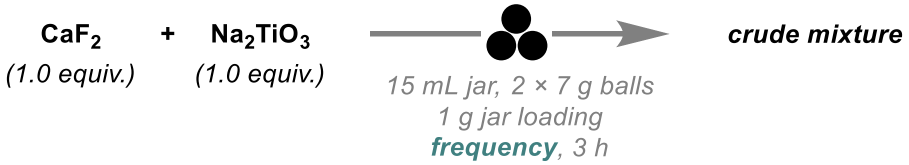

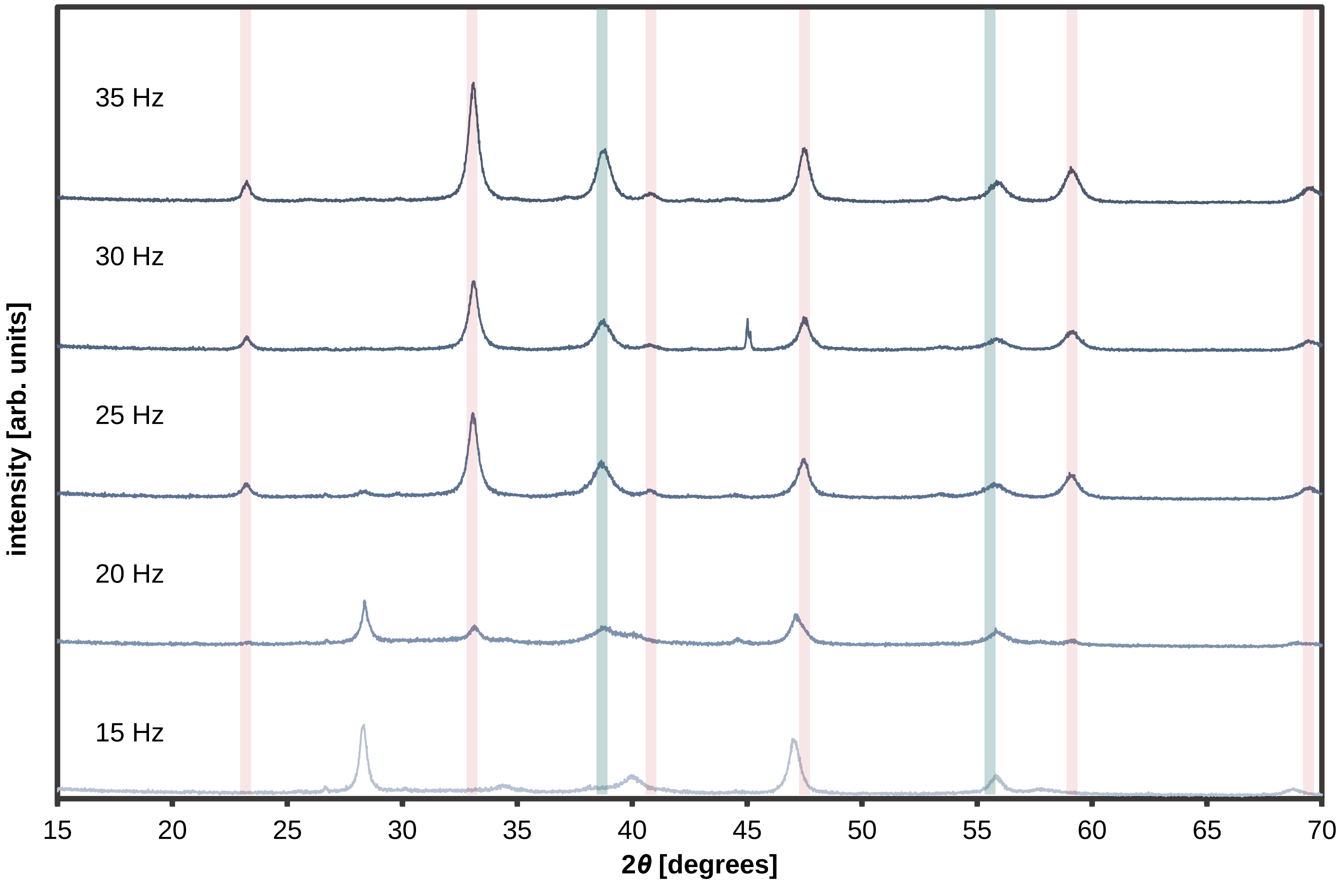

Figure S12. PXRD traces of crude mixtures obtained after milling AGF with Na_2TiO_3 at various frequencies, with diagnostic reflections highlighted in color (NaF = green, $CaTiO_3$ = red). PDF# $CaTiO_3$: 01-078-1013; NaF: 01-075-0448; CaF_2: 01-077-2093; Na_2TiO_3: 01-080-6123.

5. Comparative Study between Alkali Metal Hydroxides

5.1. Intrinsic Stabilities of Alkali Metal Fluorides in Presence of AGF

To a 15 mL stainless steel milling jar was added one hardened chrome steel bearing (7 g), AGF (1 equiv.) and alkali metal fluoride (1.0 equiv.) to give approximately 1 g of material for milling. The jar was then closed and securely fitted to the mill which was set for 3 h at the frequency of 35 Hz. Upon completion, the jar was opened, the powder was collected, and analyzed by PXRD.

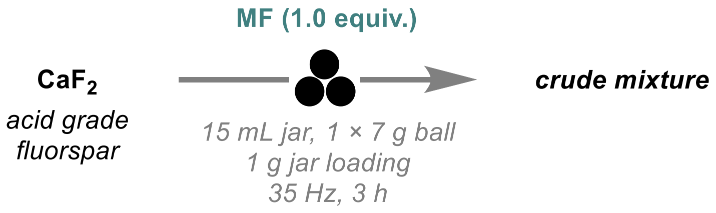

Table S13. Comparison of instrinsic reactivity between alkali metal fluorides and AGF.

Entry	MF	*t* [a]	PXRD (major species)
1	LiF	0.90	*LiF*, *CaF_2*
2	NaF	0.83	*NaF*, *CaF_2*
3	KF	0.90	$KCaF_3$, *CaF_2*

[a] Goldschmidt tolerance factors (*t*) are taken from reference.[20]

5.2. Binary Systems for the Activation of AGF

To a 15 mL stainless steel milling jar was added two hardened chrome steel bearings (2 × 7 g), AGF (1 equiv.) and alkali metal hydroxide (2.0 equiv.) to give approximately 1 g of material for milling. The jar was then closed and securely fitted to the mill which was set for 3 h at the frequency of 35 Hz. Upon completion, the jar was opened, the powder was collected, and analyzed by PXRD. An aliquot of the obtained mixture (approximately 50 mg) and sodium triflate (approximately 10 mg) as internal standard was extracted with D_2O (10 atom% D), centrifugated, and the supernatant was analyzed by quantitative ^{19}F NMR spectroscopy.

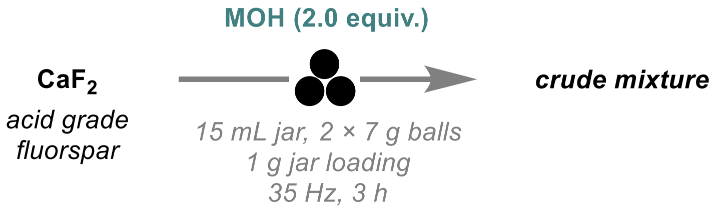

Table S14. Comparison of alkali metal hydroxides as activators for AGF.

Entry	MOH	ΔU_{POT}^{BHFC} [kJ·mol^{-1}][a]	F^- release[b]	PXRD (major species)
1	LiOH	+28	13%[c]	LiF, $Ca(OH)_2$, *CaF_2 (trace)*
2	NaOH	+62	8%	NaF, $Ca(OH)_2$, *CaF_2 (trace)*
3	KOH	+52	7%	$KCaF_3$, KF, $Ca(OH)_2$

[a] Changes in lattice energies for reactions (CaF_2 + 2 MOH → $Ca(OH)_2$ + 2 MF) were calculated based on values from reference.[17]
[b] Yields were determined by quantitative ^{19}F NMR spectroscopy in D_2O (10 atom% D) using sodium triflate as an internal standard.
[c] Fluoride release might be underestimated due to low solubility of LiF in water (0.13 g per 100 g at 25 °C, reference[17]).

5.3. Ternary Systems for the Activation of AGF

To a 15 mL stainless steel milling jar was added two hardened chrome steel bearings (2 × 7 g), AGF (1 equiv.), alkali metal hydroxide (2.0 equiv.), and TiO_2 (1.0 equiv.) to give approximately 1 g of material for milling. The jar was then closed and securely fitted to the mill which was set for 3 h at the frequency of 35 Hz. Upon completion, the jar was opened, the powder was collected, and analyzed by PXRD. An aliquot of the obtained mixture (approximately 10-50 mg) and sodium triflate (approximately 10 mg) as internal standard was extracted with D_2O (10 atom% D), centrifugated, and the supernatant was analyzed by quantitative ^{19}F NMR spectroscopy.

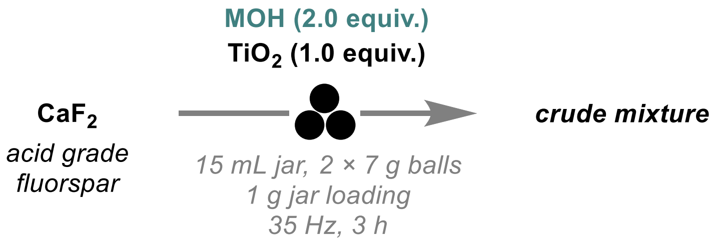

Table S15. Comparison of alkali metal hydroxides as activators for AGF in ternary systems.

Entry	MOH	F^- release[a]	PXRD (major species)
1	LiOH	20%[b]	LiF, $CaTiO_3$, *CaF_2*, *TiO_2*
2	NaOH	81%	NaF, $CaTiO_3$, *CaF_2*
3	KOH	23%	$KCaF_3$

[a] Yields were determined by quantitative ^{19}F NMR spectroscopy in D_2O (10 atom% D) using sodium triflate as an internal standard.
[b] Fluoride release might be underestimated due to low solubility of LiF in water (0.13 g per 100 g at 25 °C, reference[17]).

5.4. Alkali Metal Titanates as Activators for AGF

To a 15 mL stainless steel milling jar was added two hardened chrome steel bearings (2 × 7 g), AGF (1 equiv.), and alkali metal titanate (1.0 equiv.), to give approximately 1 g of material for milling. The jar was then closed and securely fitted to the mill which was set for 3 h at the frequency of 35 Hz. Upon completion, the jar was opened, the powder was collected, and analyzed by PXRD. An aliquot of the obtained mixture (approximately 10-50 mg) and sodium triflate (approximately 10 mg) as internal standard was extracted with D_2O (10 atom% D), centrifugated, and the supernatant was analyzed by quantitative ^{19}F NMR spectroscopy.

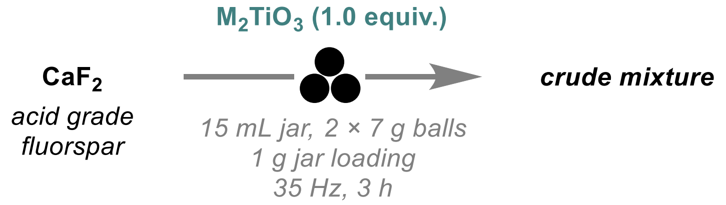

Table S16. Comparison of alkali metal titanates as activators for AGF.

Entry	M_2TiO_3	F^- release[a]	PXRD (major species)
1	Li_2TiO_3	37%[b]	$CaTiO_3$, *CaF_2*
2	Na_2TiO_3	76%	NaF, $CaTiO_3$
3	K_2TiO_3	36%	$KCaF_3$, $CaTiO_3$

[a] Yields were determined by quantitative ^{19}F NMR spectroscopy in D_2O (10 atom% D) using sodium triflate as an internal standard.
[b] Fluoride release might be underestimated due to low solubility of LiF in water (0.13 g per 100 g at 25 °C, reference[17]).

To a 15 mL stainless steel milling jar was added two hardened chrome steel bearings (2 × 7 g), AGF (1 equiv.), and potassium titanate (as specified in Table S17), to give approximately 1 g of material for milling. The jar was then closed and securely fitted to the mill which was set for 3 h at the frequency of 35 Hz. Upon completion, the jar was opened, the powder was collected, and analyzed by PXRD. An aliquot of the obtained mixture (approximately 50 mg) and sodium triflate (approximately 10 mg) as internal standard was extracted with D_2O (10 atom% D), centrifugated, and the supernatant was analyzed by quantitative ^{19}F NMR spectroscopy.

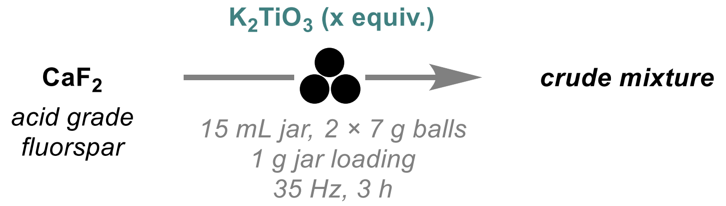

Table S17. Optimization using K_2TiO_3 as activator for AGF.

Entry	K_2TiO_3 (x equiv.)	F^- release[a]	PXRD (major species)
1	0.33	11%	$CaTiO_3$, *CaF_2*
2	0.50	17%	$CaTiO_3$, *CaF_2*
3	0.75	26%	$KCaF_3$, $CaTiO_3$, *CaF_2*
4	1.00	36%	$KCaF_3$, $CaTiO_3$
5	1.25	46%	$KCaF_3$, $CaTiO_3$
6	1.50	49%	$KCaF_3$, $CaTiO_3$

[a] Yields were determined by quantitative ^{19}F NMR spectroscopy in D_2O (10 atom% D) using sodium triflate as an internal standard.

6. Solid-state NMR Spectroscopy

6.1. Binary Systems

^{19}F SS NMR spectra were recorded on a sample of AGF that had been milled for 3 h at 35 Hz with 2.0 equiv. LiOH (Figure S13). ^{19}F DPMAS SS NMR spectroscopy of starting AGF reveals a single, intense resonance at δ = −107.1 ppm, in line with literature data.[21] Following mechanochemical activation, two resonances were observed, a small amount of inactivated, residual CaF_2 was observed at δ = −107.1 ppm accounting for 1.8% of the signal, along with the growing in of an intense new resonance at δ = −204.0 ppm accounting for the remaining 98.2%. This upfield resonance can be assigned as LiF, in line with the literature value of −204 ppm.[22,23]

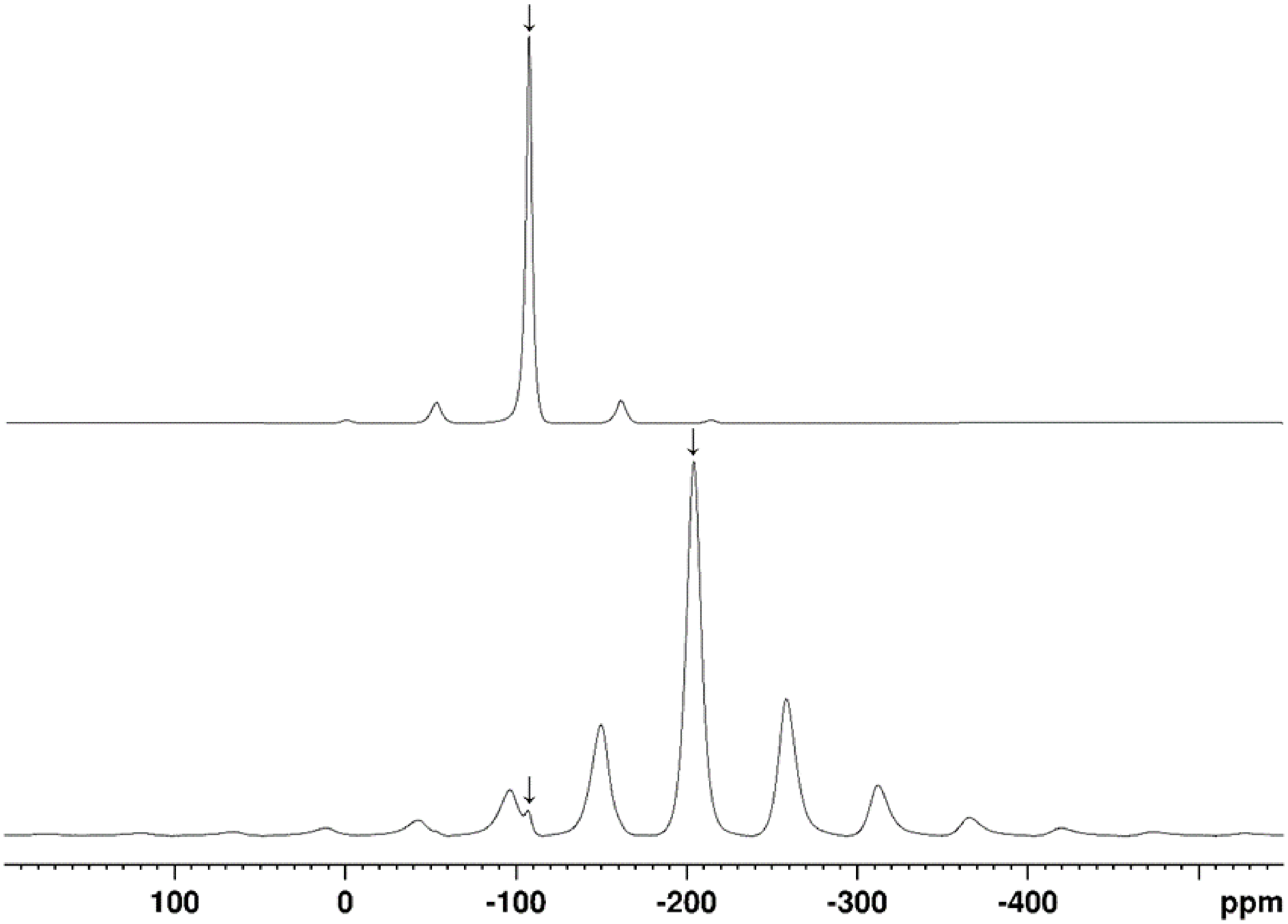

Figure S13. ^{19}F DPMAS SS NMR spectra of AGF before (*top*) and after (*bottom*) activation with 2.0 equiv. LiOH. Isotropic resonances denoted by an arrow (↓), ν_{rot} = 20 kHz.

^{19}F SS NMR spectra were recorded on a sample of AGF that had been milled for 3 h at 35 Hz with 1.0 equiv. Li_2O (Figure S14). Following mechanochemical activation, a small amount of inactivated, residual CaF_2 was observed at δ = −107.1 ppm accounting for 2.8% of the signal, along with an intense resonance associated with LiF at δ = −204.5 ppm accounting for 97.2% of the fluorine content of the sample.

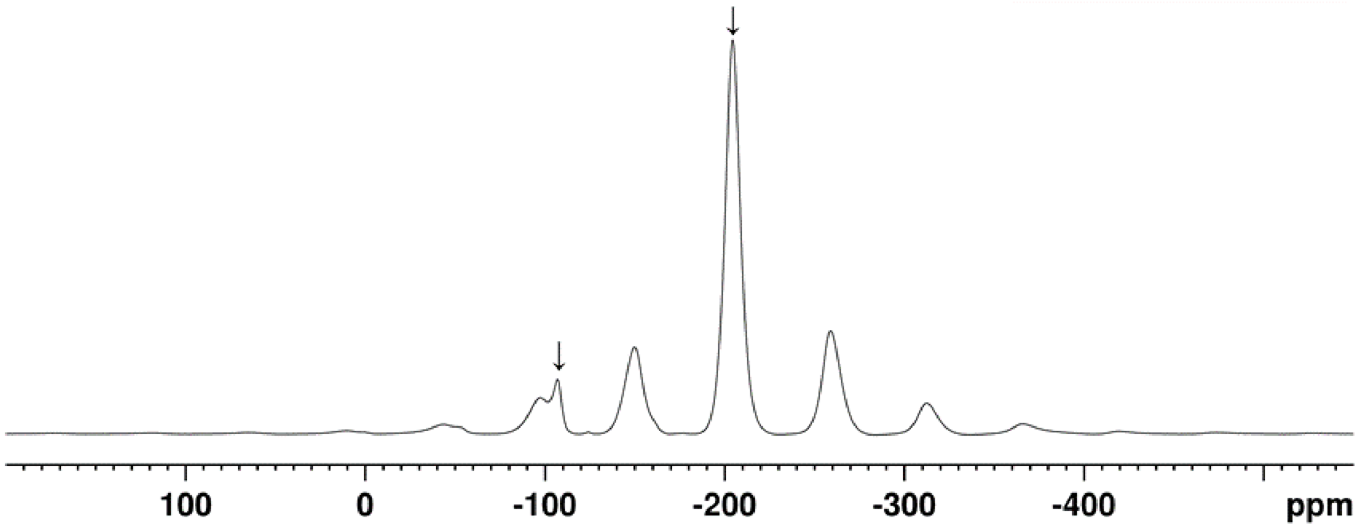

Figure S14. ^{19}F DPMAS SS NMR spectra of CaF_2 after activation with 1.0 equiv. of Li_2O. Isotropic resonances denoted by an arrow (↓), ν_{rot} = 20 kHz.

^{19}F SS NMR spectra were recorded on a sample of AGF that had been milled for 3 h at 35 Hz with 2.0 equiv. NaOH (Figure S15). Following mechanochemical activation, a small amount of inactivated, residual CaF_2 was observed at δ = −107.2 ppm accounting for 11.8% of the signal, along with the growing in of an intense new resonance at δ = −224.2 ppm accounting for 77.9%. This upfield resonance can be assigned as NaF, in line with the literature value of −224 ppm.[24] A third isotropic resonance was observed at δ = −184.8 ppm, which contributes the remaining 10.3% of the fluorine signal, has not been assigned.

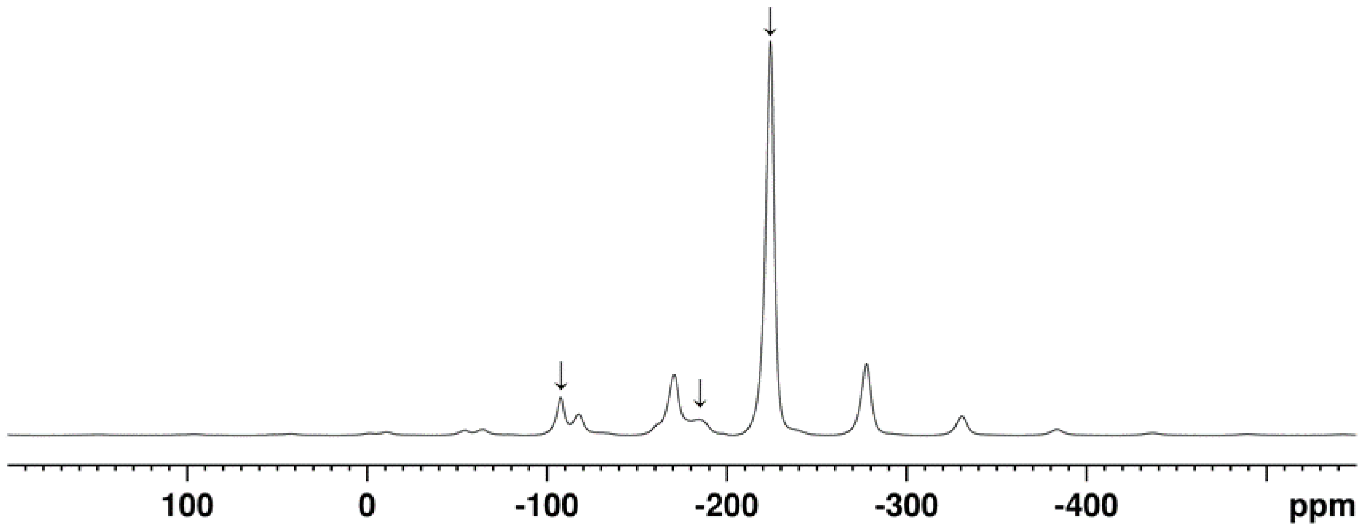

Figure S15. ^{19}F DPMAS SS NMR spectra of AGF after activation with 2.0 equiv. NaOH. Isotropic resonances denoted by an arrow (↓), ν_{rot} = 20 kHz.

^{19}F SS NMR spectra were recorded on a sample of AGF that had been milled for 3 h at 35 Hz with 1.0 equiv. Na_2O (Figure S16). Following mechanochemical activation, trace amounts of inactivated, residual CaF_2 were observed at δ = −107.2 ppm accounting for <1% of the signal, along with an intense new resonance associated with NaF at δ = −224.2 ppm accounting for 82.2% of the fluorine content of the sample. The remaining 17.7% of the fluorine signal has not been assigned.

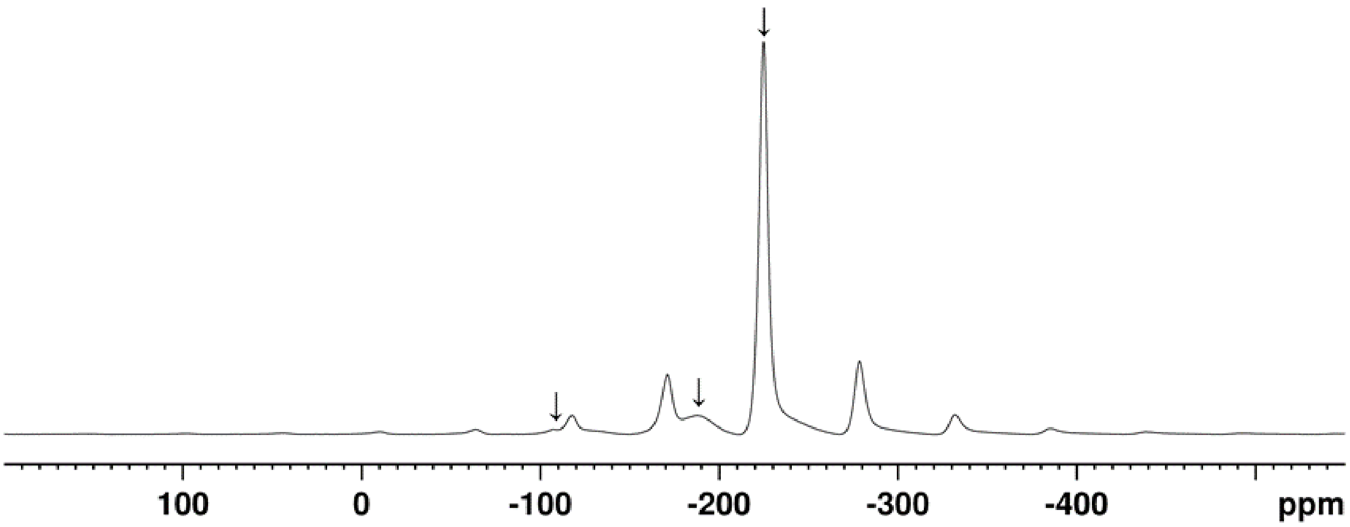

Figure S16. ^{19}F DPMAS SS NMR spectra of AGF after activation with 1.0 equiv. Na_2O. Isotropic resonances denoted by an arrow (↓), ν_{rot} = 20 kHz.

^{19}F SS NMR spectra were recorded on a sample of AGF that had been milled for 3 h at 35 Hz with 2.0 equiv. KOH (Figure S17). Following mechanochemical activation, a small shoulder of residual CaF_2 was observed at δ = −107 ppm accounting for 0.5% of the signal. A sharp resonance which can be assigned as anhydrous KF, contributing 13.3% of the signal of the spectra was observed at −133.1 ppm, in line with the literature value of −130.2 ppm.[22,25] The remaining 86.2% of the signal is accounted for by the broad resonance at δ = −122.6 ppm, which was assigned as the fluoroperovskite $KCaF_3$, in line with the literature value of −120.9 ppm.[26]

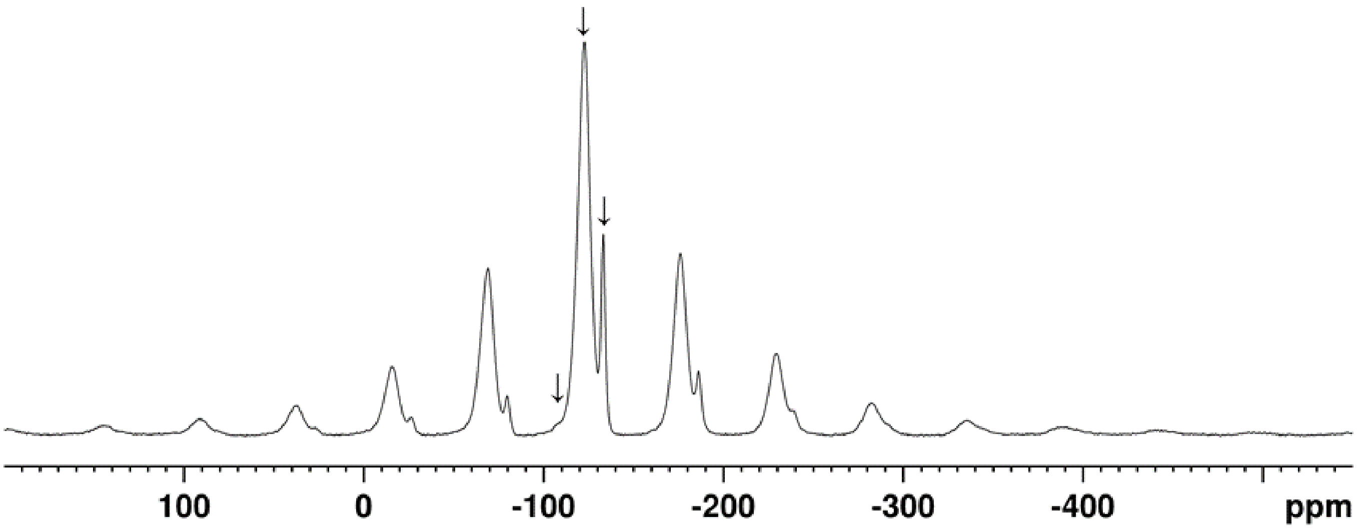

Figure S17. ^{19}F DPMAS SS NMR spectra of AGF after activation with 2.0 equiv. KOH. Isotropic resonances denoted by an arrow (↓), ν_{rot} = 20 kHz.

^{19}F SS NMR spectra were recorded on a sample of AGF that had been milled for 3 h at 35 Hz with 2.0 equiv. NaCl (Figure S18). Following mechanochemical activation, only residual CaF_2 was observed at δ = −108.8 ppm accounting for 100% of the signal.

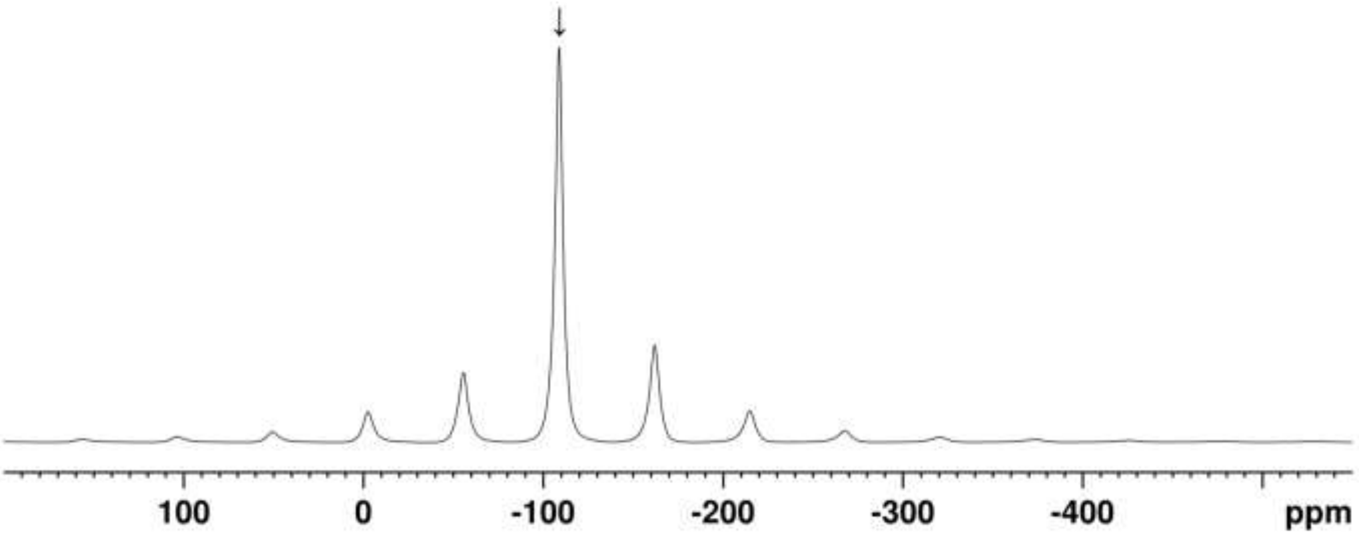

Figure S18. ^{19}F DPMAS SS NMR spectra of AGF after activation with 2.0 equiv. NaCl. Isotropic resonances denoted by an arrow (↓), ν_{rot} = 20 kHz.

^{19}F SS NMR spectra were recorded on a sample of AGF that had been milled for 3 h at 35 Hz with 1.0 equiv. Na_2CO_3 (Figure S19). Following mechanochemical activation, NaF was observed at δ = −224.8 ppm accounting for 12.5% of the signal, alongside residual CaF_2 at δ = −108.5 ppm accounting for the remaining 87.5% of the signal.

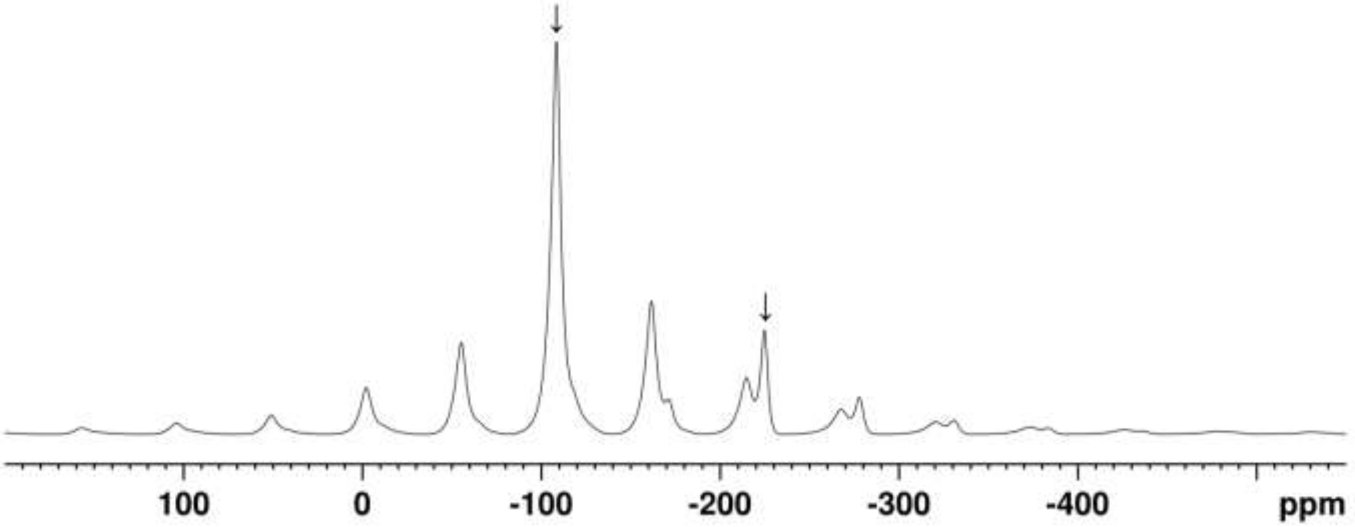

Figure S19. ^{19}F DPMAS SS NMR spectra of AGF after activation with 1.0 equiv. Na_2CO_3. Isotropic resonances denoted by an arrow (↓), ν_{rot} = 20 kHz.

^{19}F SS NMR spectra were recorded on a sample of AGF that had been milled for 3 h at 35 Hz with 0.67 equiv. Na_3PO_4 (Figure S20). Following mechanochemical activation, 3 resonances were observed. Residual unactivated CaF_2 was observed at δ = −108.7 ppm accounting for 25.2% of the signal, alongside NaF at δ = −225.1 ppm accounting for 40.4% of the signal. A third, broad resonance was also observed at −135.6 ppm accounting for 34.4% of the signal. This resonance can be attributed to the formation of an intermediate, mixed, Na/Ca phosphate phase, nacaphite (Na_2CaPO_4F), observed by PXRD, Table S1.

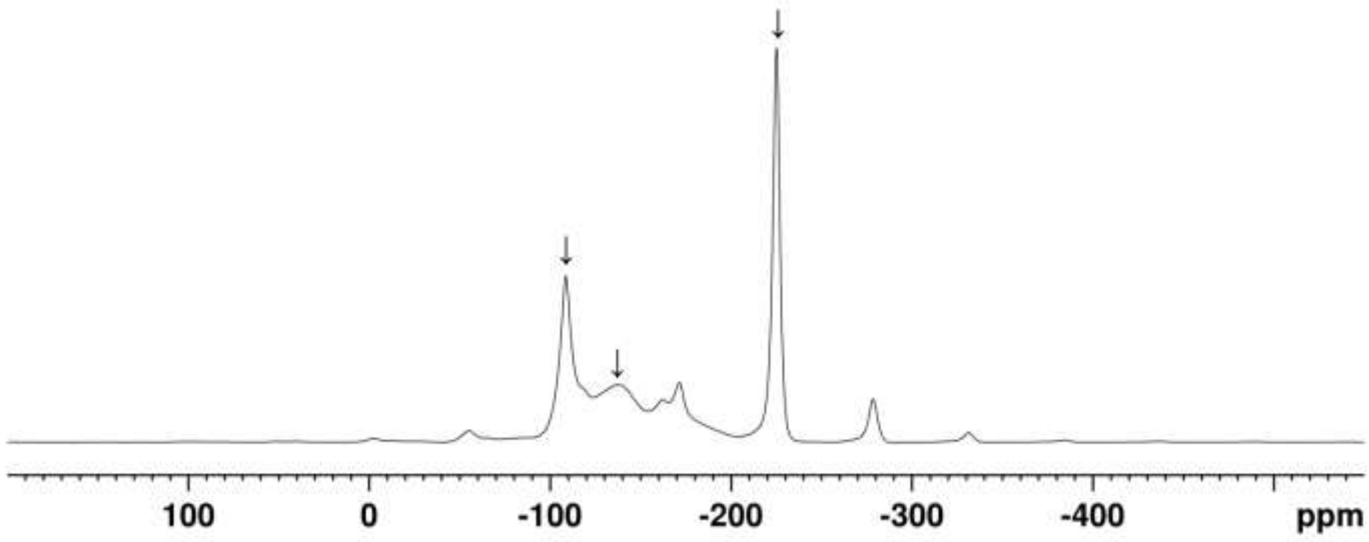

Figure S20. ^{19}F DPMAS SS NMR spectra of AGF after activation with 0.67 equiv. Na_3PO_4. Isotropic resonances denoted by an arrow (↓), ν_{rot} = 20 kHz.

^{19}F SS NMR spectra were recorded on a sample of AGF that had been milled for 3 h at 35 Hz with 1.0 equiv. Na_2S (Figure S21). Following mechanochemical activation, NaF was observed at δ = −225.2 ppm accounting for 95.6% of the signal, alongside residual CaF_2 at δ = −108.8 ppm accounting for 4.4% of the signal.

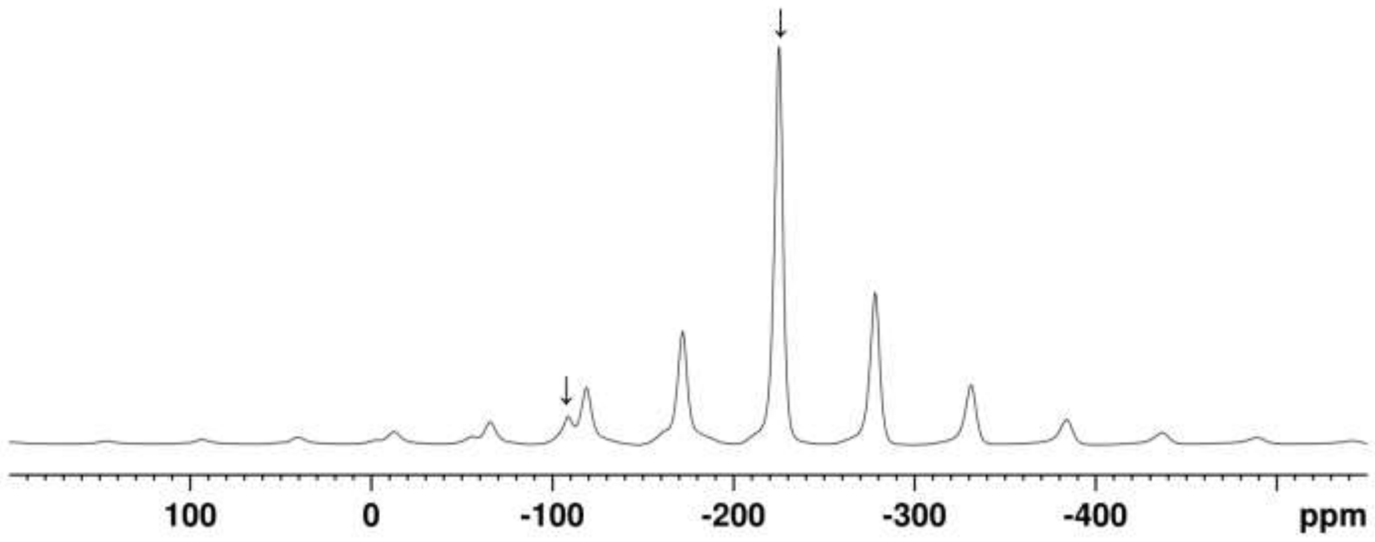

Figure S21. ^{19}F DPMAS SS NMR spectra of AGF after activation with 1.0 equiv. Na_2S. Isotropic resonances denoted by an arrow (↓), ν_{rot} = 20 kHz.

^{19}F SS NMR spectra were recorded on a sample of AGF that had been milled for 3 h at 35 Hz with 1.0 equiv. Na_2SO_4 (Figure S22). Following mechanochemical activation, residual CaF_2 was observed at δ = −108.7 ppm accounting for 100% of the signal.

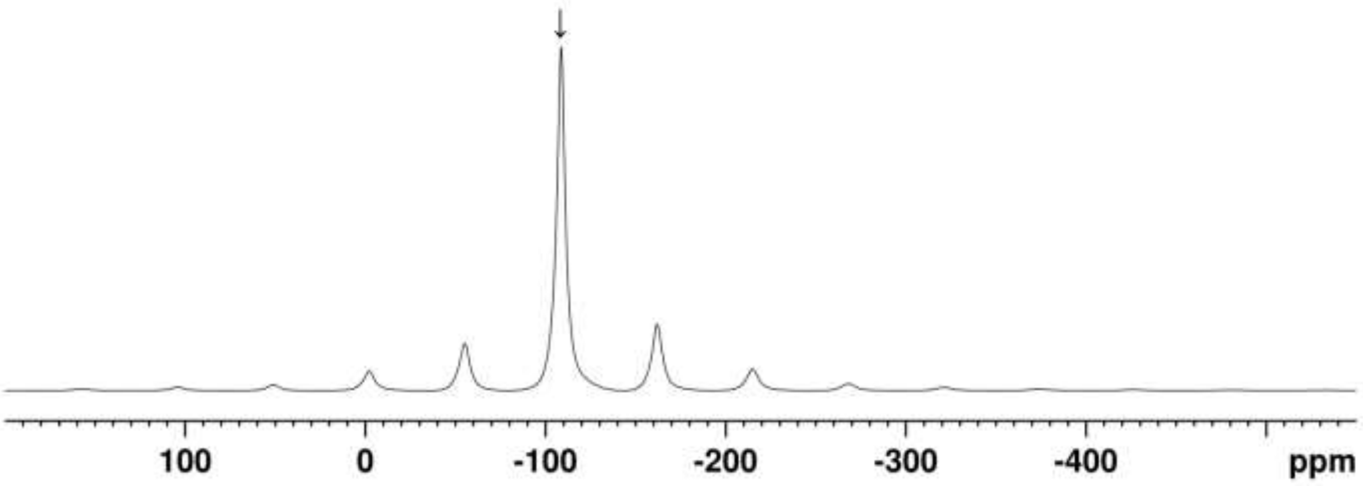

Figure S22. ^{19}F DPMAS SS NMR spectra of AGF after activation with 1.0 equiv. Na_2SO_4. Isotropic resonances denoted by an arrow (↓), ν_{rot} = 20 kHz.

6.2. Ternary Systems

^{19}F SS NMR spectra were recorded on a sample of AGF that had been milled for 3 h at 35 Hz with 2.0 equiv. LiOH and 1.0 equiv. TiO_2 (Figure S23). Following mechanochemical activation, a significant amount of inactivated, residual CaF_2 was observed at δ = −108.4 ppm accounting for 56.4% of the signal, along with a resonance associated with LiF at δ = −202.7 ppm accounting for 43.6% of the fluorine content of the sample.

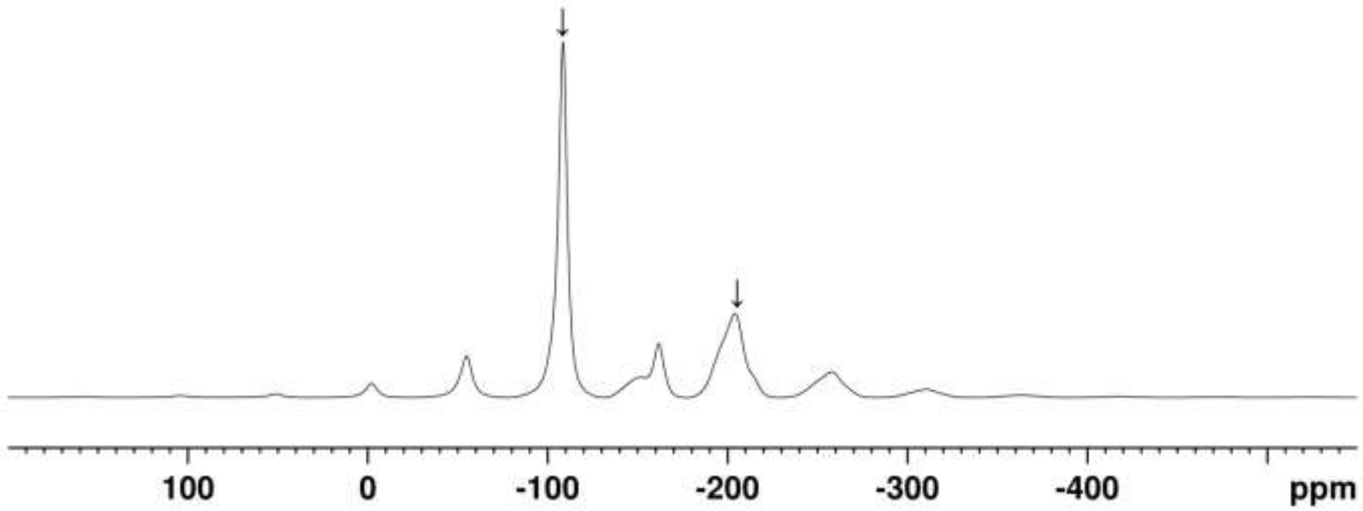

Figure S23. ^{19}F DPMAS SS NMR spectra of AGF after activation-sequestration with 2.0 equiv. LiOH and 1.0 equiv. TiO_2. Isotropic resonances denoted by an arrow (↓), ν_{rot} = 20 kHz.

^{19}F SS NMR spectra were recorded on a sample of AGF that had been milled for 3 h at 35 Hz with 1.0 equiv. Li_2O and 1.0 equiv. TiO_2 (Figure S24). Following mechanochemical activation, a small amount of inactivated, residual CaF_2 was observed at δ = −107.7 ppm accounting for 7.4% of the signal, along with an intense resonance associated with LiF at δ = −204.8 ppm accounting for 92.6% of the fluorine content of the sample.

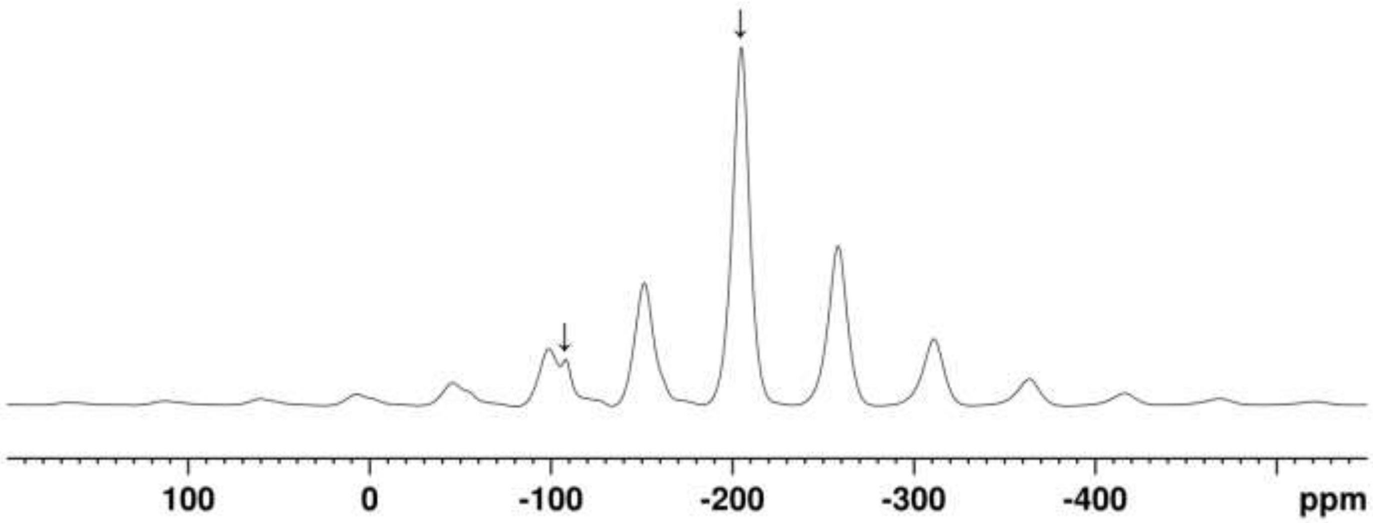

Figure S24. ^{19}F DPMAS SS NMR spectra of AGF after activation-sequestration with 1.0 equiv. Li_2O and 1.0 equiv. TiO_2. Isotropic resonances denoted by an arrow (↓), ν_{rot} = 20 kHz.

^{19}F SS NMR spectra were recorded on a sample of AGF that had been milled for 3 h at 35 Hz with 2.0 equiv. NaOH and 1.0 equiv. TiO_2 (Figure S25). Following mechanochemical activation, trace amounts of inactivated, residual CaF_2 were observed at δ = −108.9 ppm accounting for 6.1% of the signal, along with an intense new resonance associated with NaF at δ = −225.0 ppm accounting for 88.1% of the fluorine content of the sample. The remaining 5.8% of the fluorine signal has not been assigned.

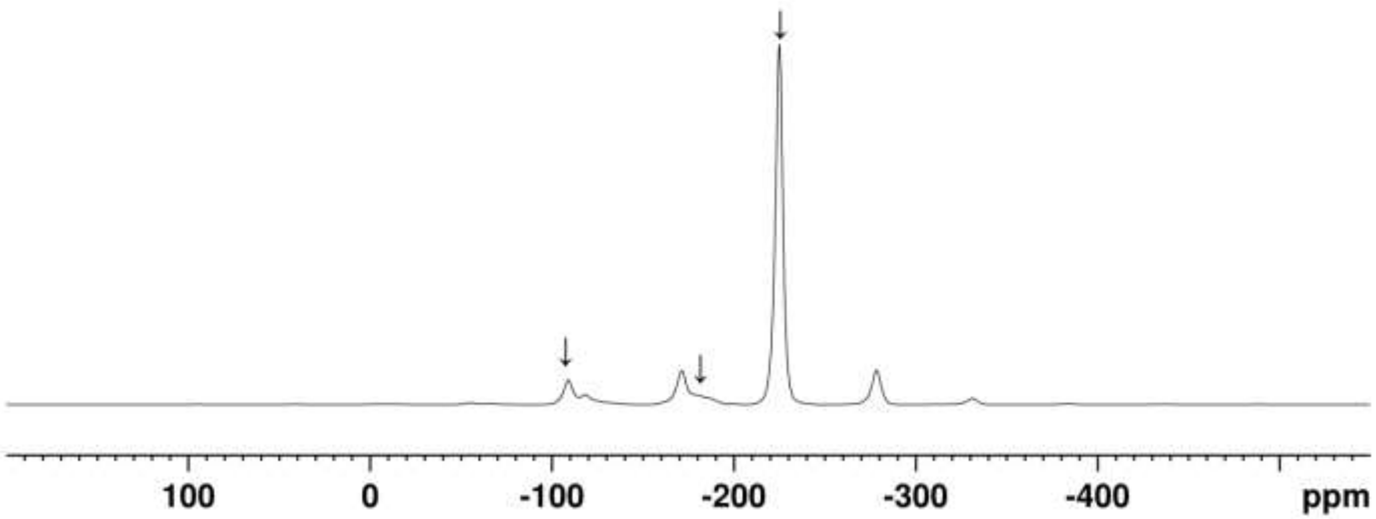

Figure S25. ^{19}F DPMAS SS NMR spectra of AGF after activation-sequestration with 2.0 equiv. NaOH and 1.0 equiv. TiO_2. Isotropic resonances denoted by an arrow (↓), ν_{rot} = 20 kHz.

^{19}F SS NMR spectra were recorded on a sample of AGF that had been milled for 3 h at 35 Hz with 2.0 equiv. KOH and 1.0 equiv. TiO_2 (Figure S26). Following mechanochemical activation, a small shoulder of residual CaF_2 was observed at δ = −109.5 ppm accounting for 4.9% of the signal. An intense resonance which can be assigned as the fluoroperovskite $KCaF_3$, contributing 95.1% of the signal of the spectra was observed at −123.6 ppm.

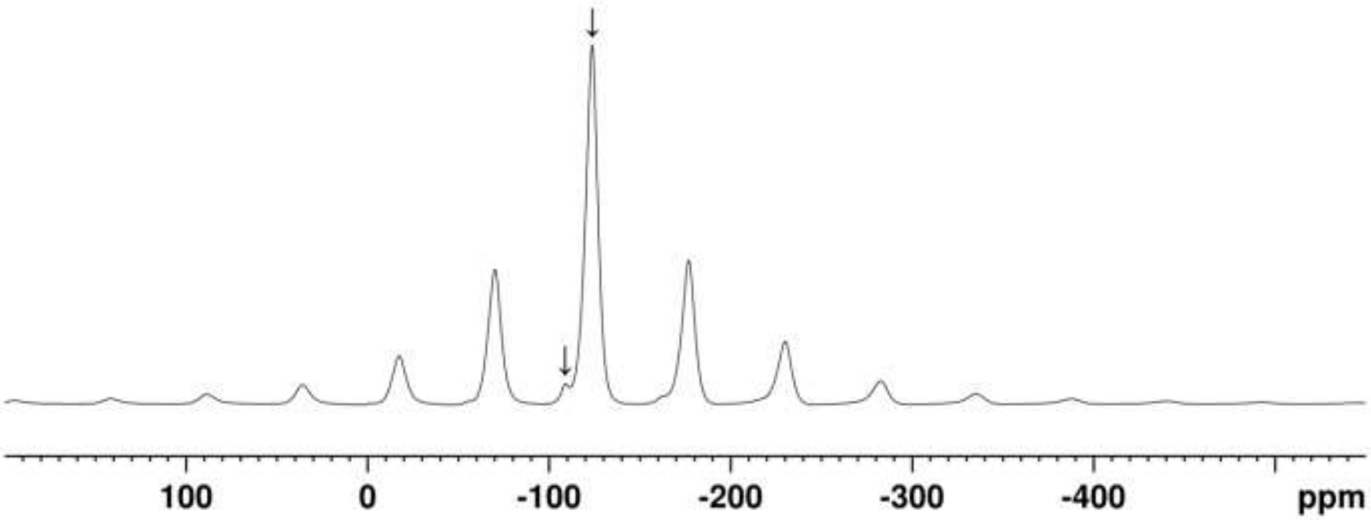

Figure S26. ^{19}F DPMAS SS NMR spectra of AGF after activation-sequestration with 2.0 equiv. KOH and 1.0 equiv. TiO_2. Isotropic resonances denoted by an arrow (↓), ν_{rot} = 20 kHz.

7. Simulated PXRD Reference Patterns

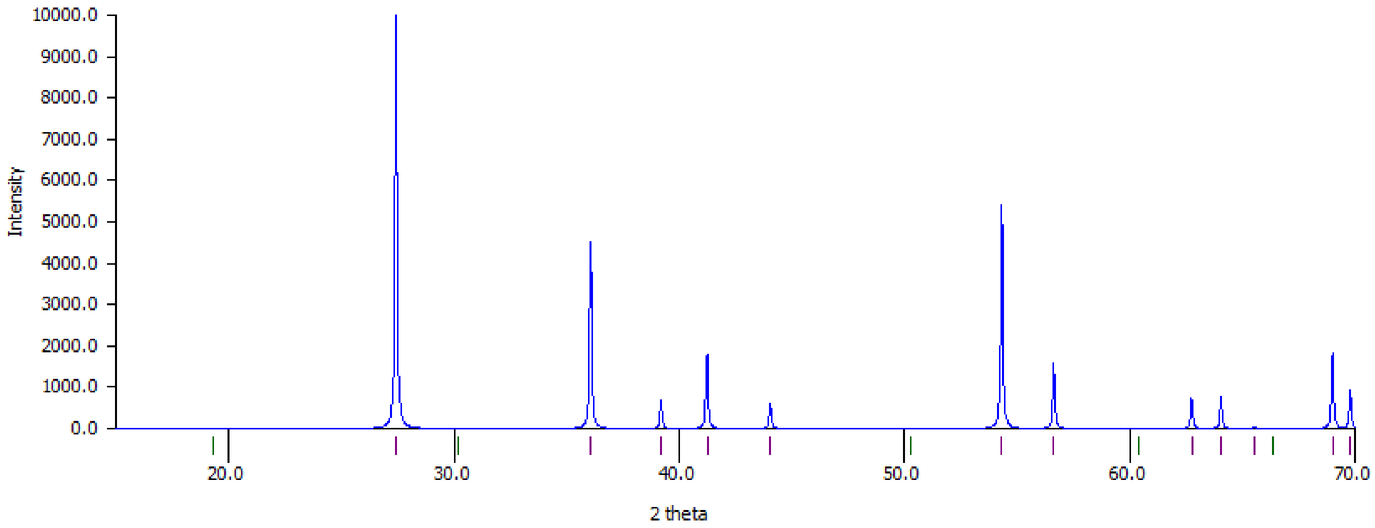

Figure S27. Simulated PXRD pattern of TiO_2 (rutile) (PDF# 01-089-4202).

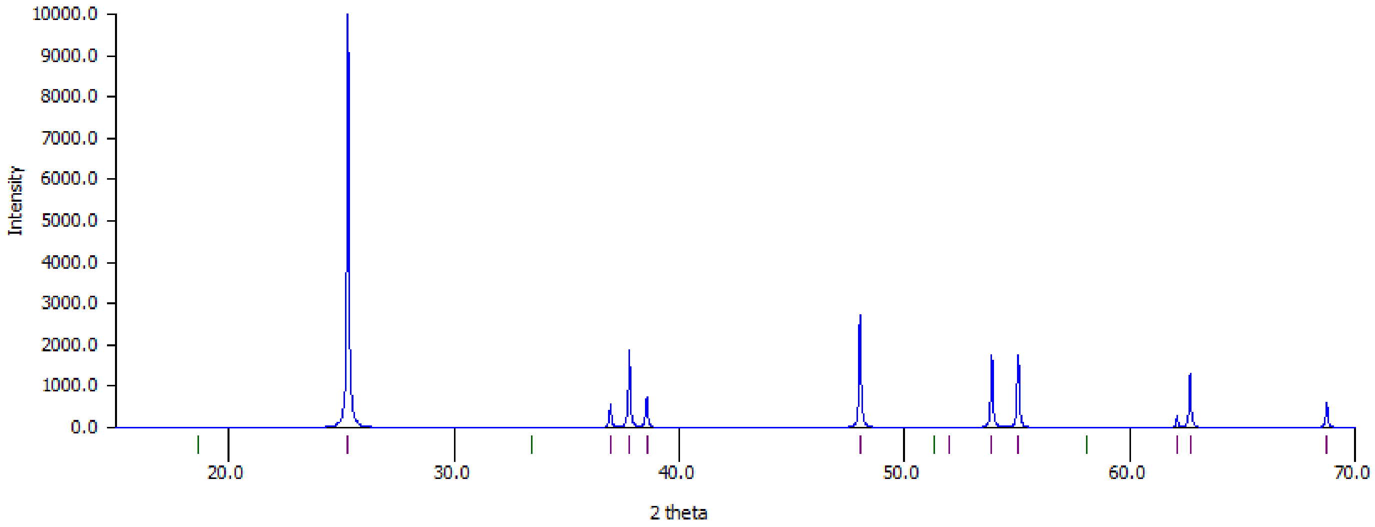

Figure S28. Simulated PXRD pattern of TiO_2 (anatase) (PDF# 01-089-4203).

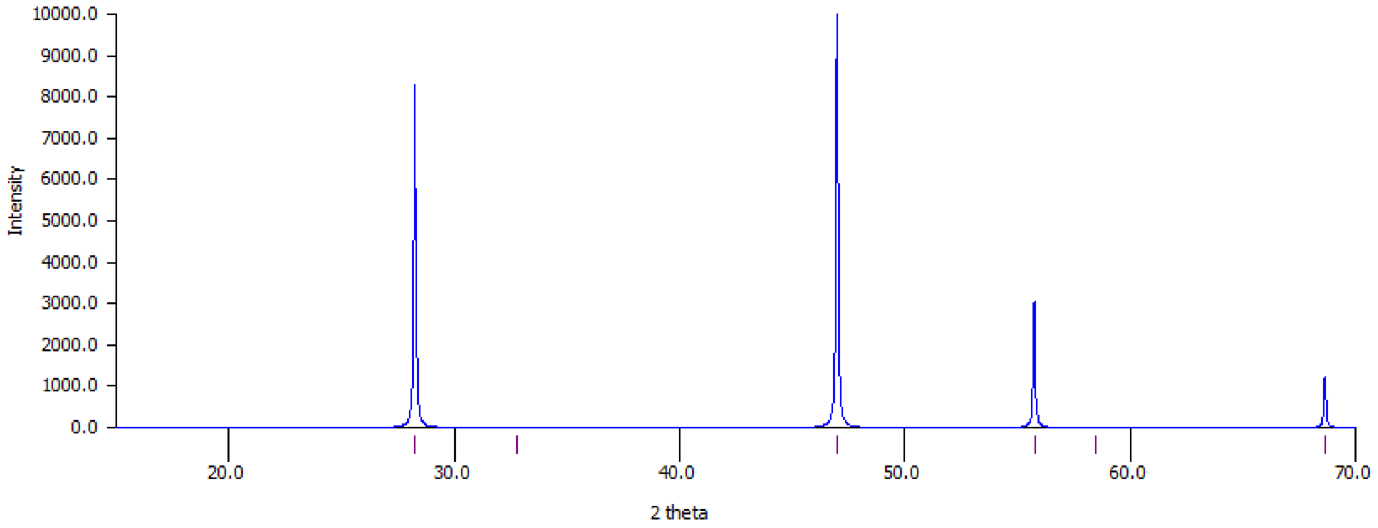

Figure S29. Simulated PXRD pattern of CaF_2 (PDF# 01-077-2093).

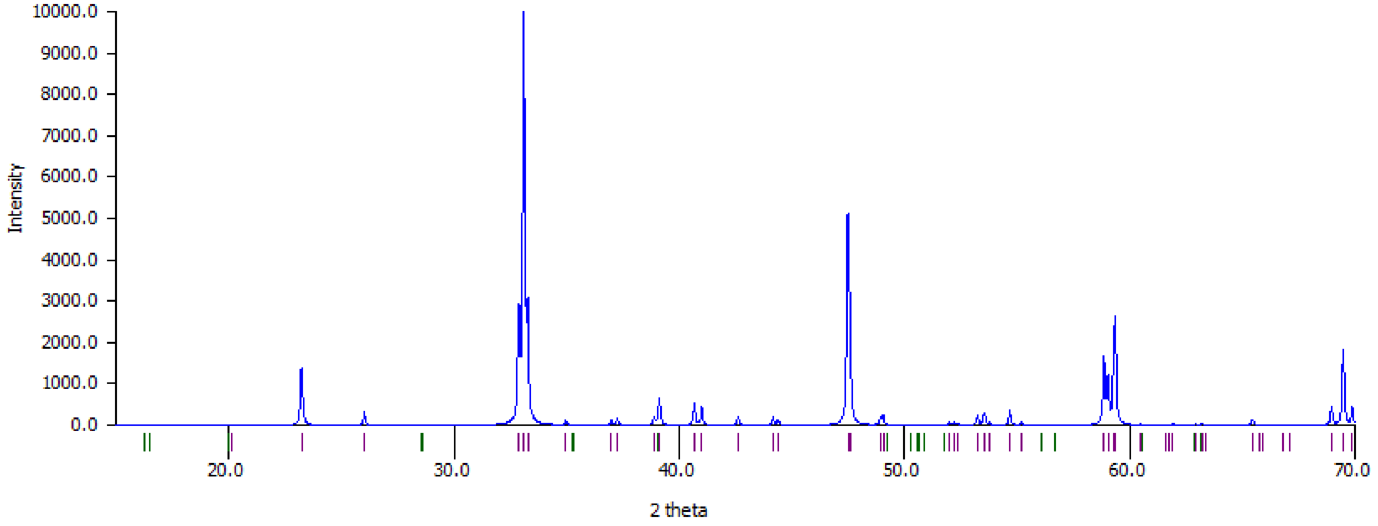

Figure S 30. Simulated PXRD pattern of $CaTiO_3$ (PDF# 01-078-1013).

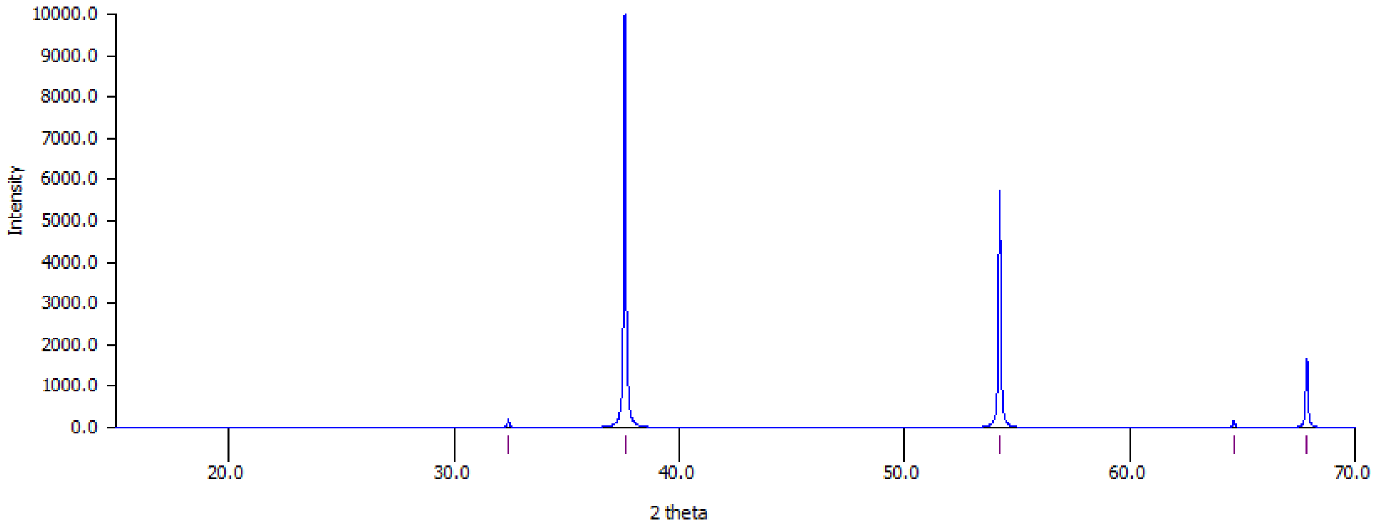

Figure S31. Simulated PXRD pattern of NaF (PDF# 01-075-0448).

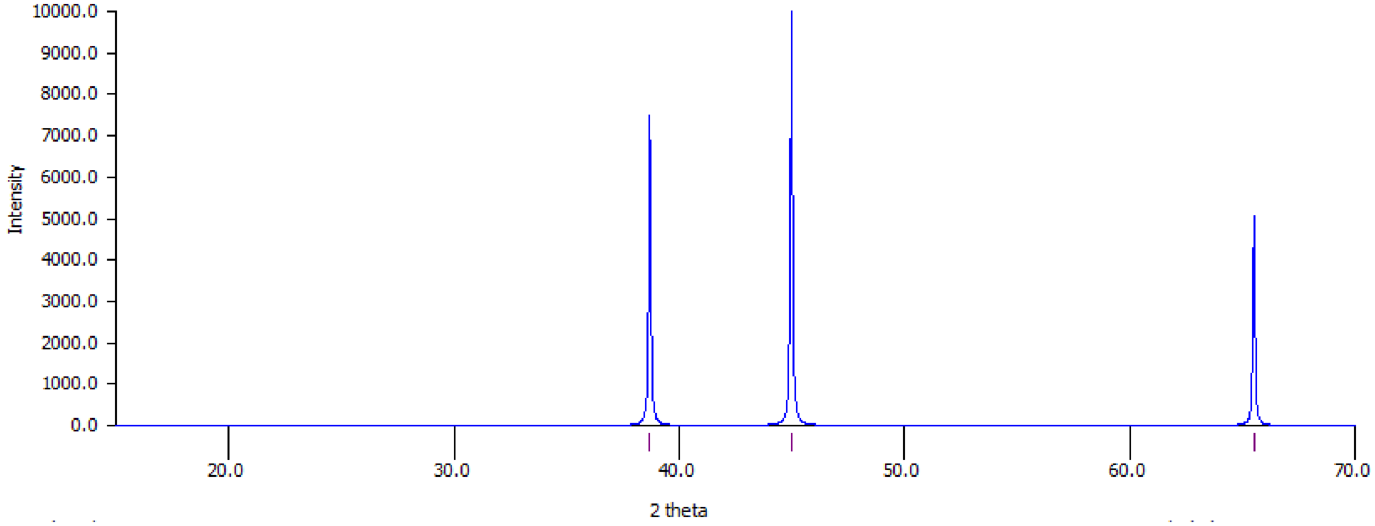

Figure S32. Simulated PXRD pattern of LiF (PDF# 01-085-8944).

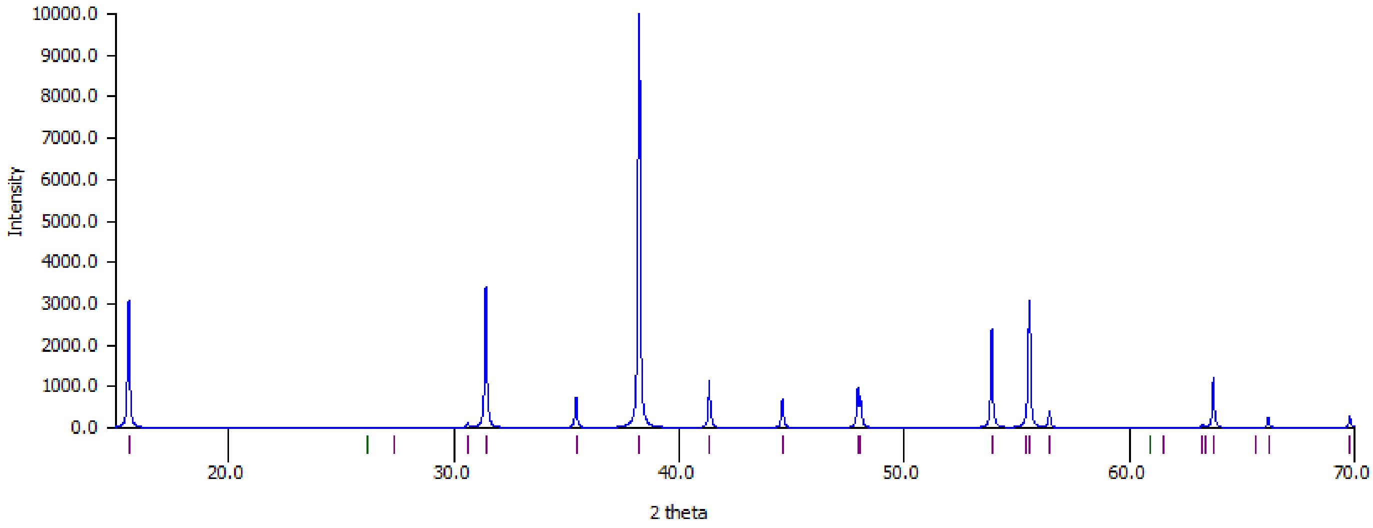

Figure S 33. Simulated PXRD pattern of NaOH (PDF# 01-085-0732).

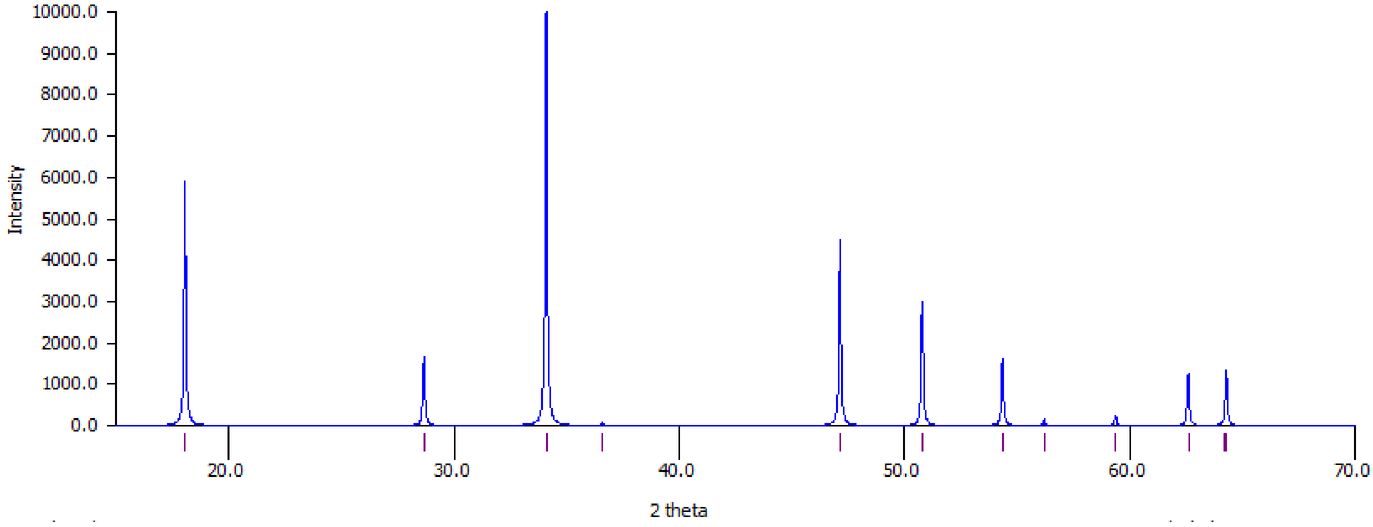

Figure S 34. Simulated PXRD pattern of $Ca(OH)_2$ (PDF# 01-076-0571).

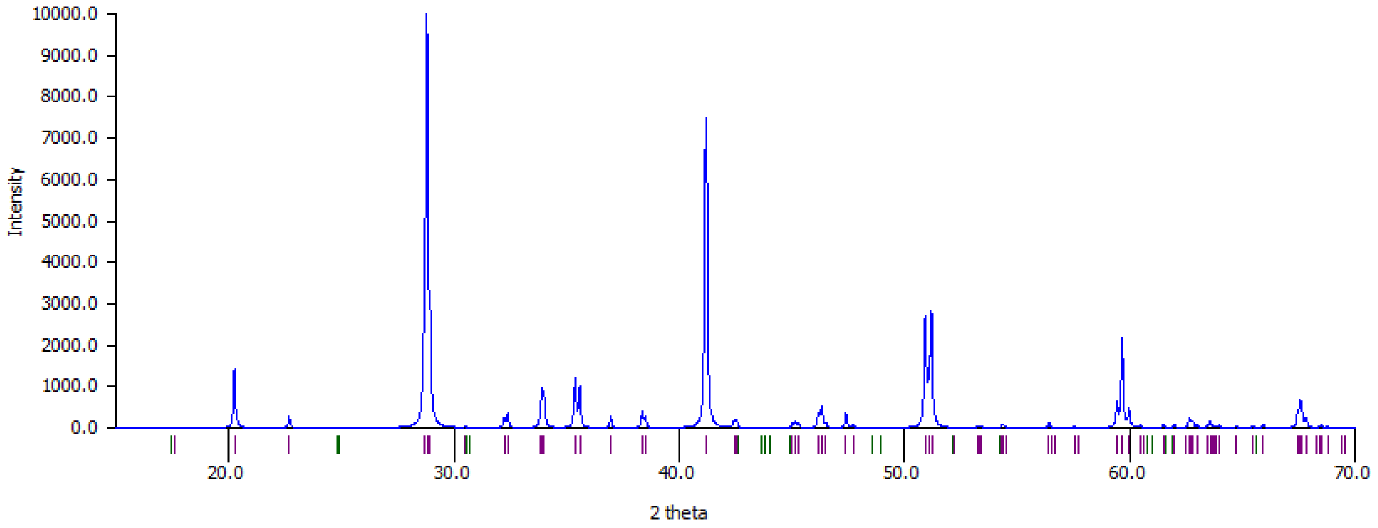

Figure S35. Simulated PXRD pattern of $KCaF_3$ (PDF# 01-074-9131).

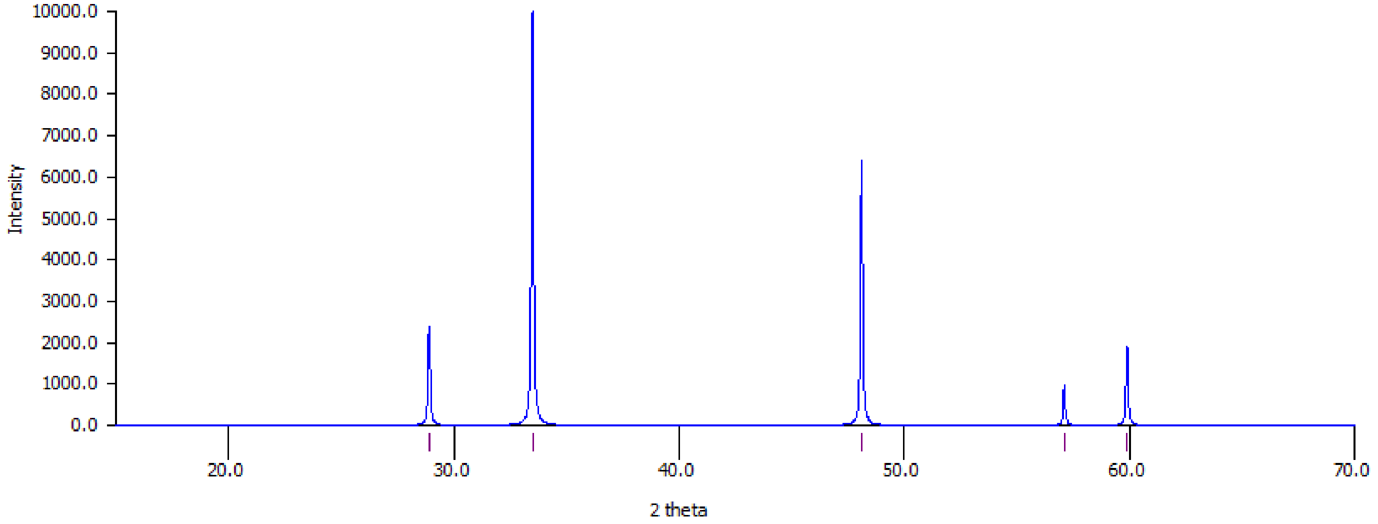

Figure S36. Simulated PXRD pattern of KF (PDF# 01-085-1314).

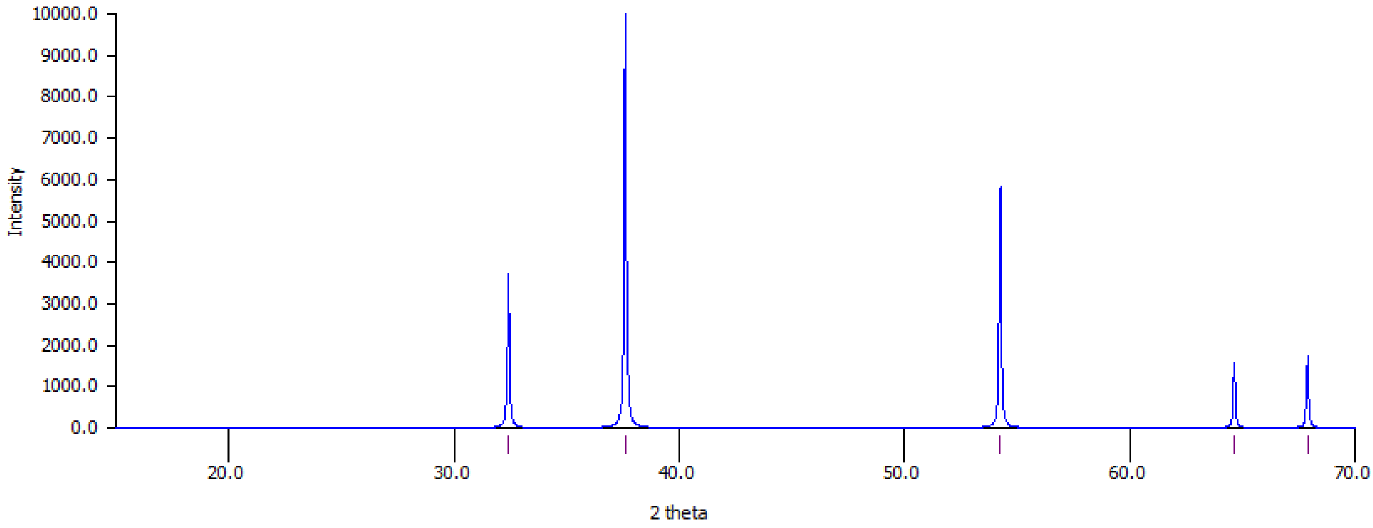

Figure S37. Simulated PXRD pattern of CaO (PDF# 01-074-1226).

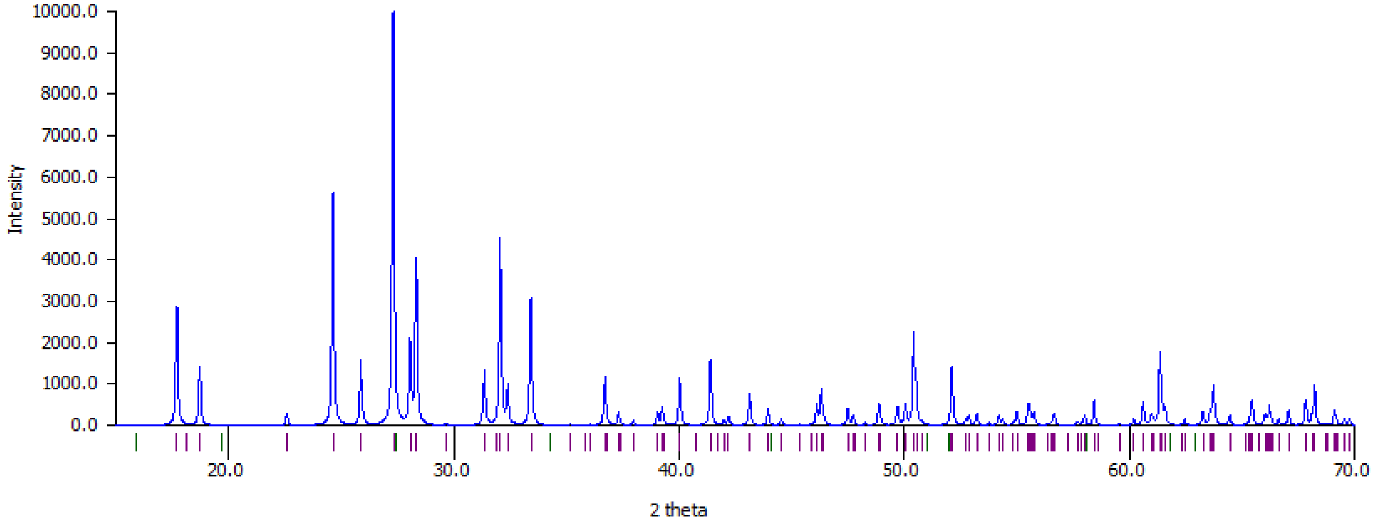

Figure S38. Simulated PXRD pattern of $NaVO_3$ (PDF# 01-070-1015).

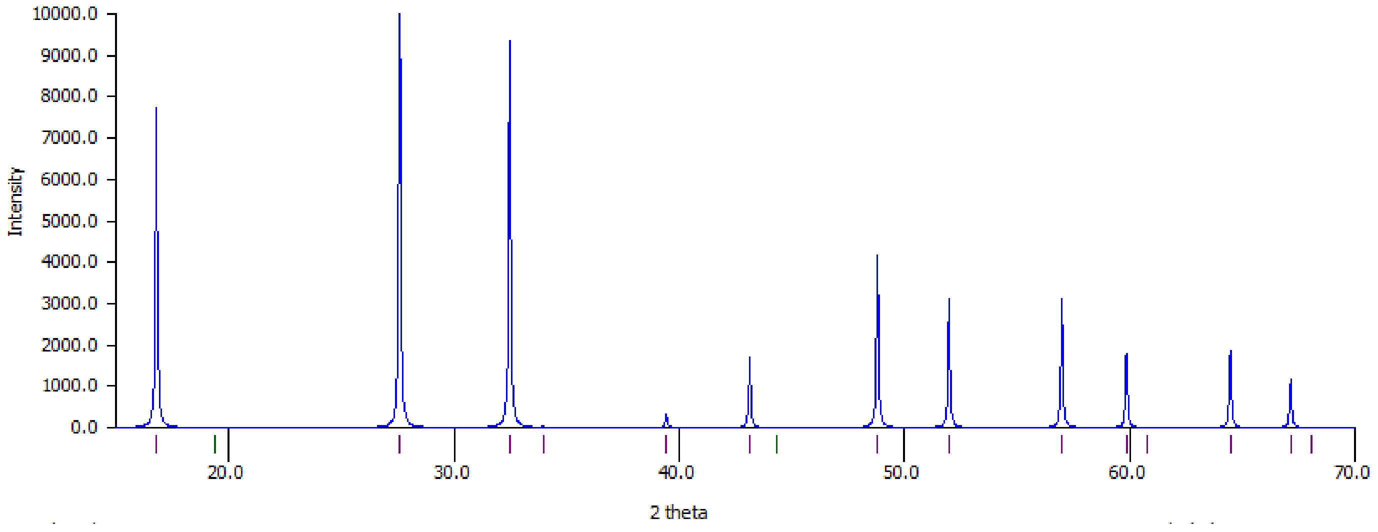

Figure S39. Simulated PXRD pattern of Na_2WO_4 (PDF# 01-070-1040).

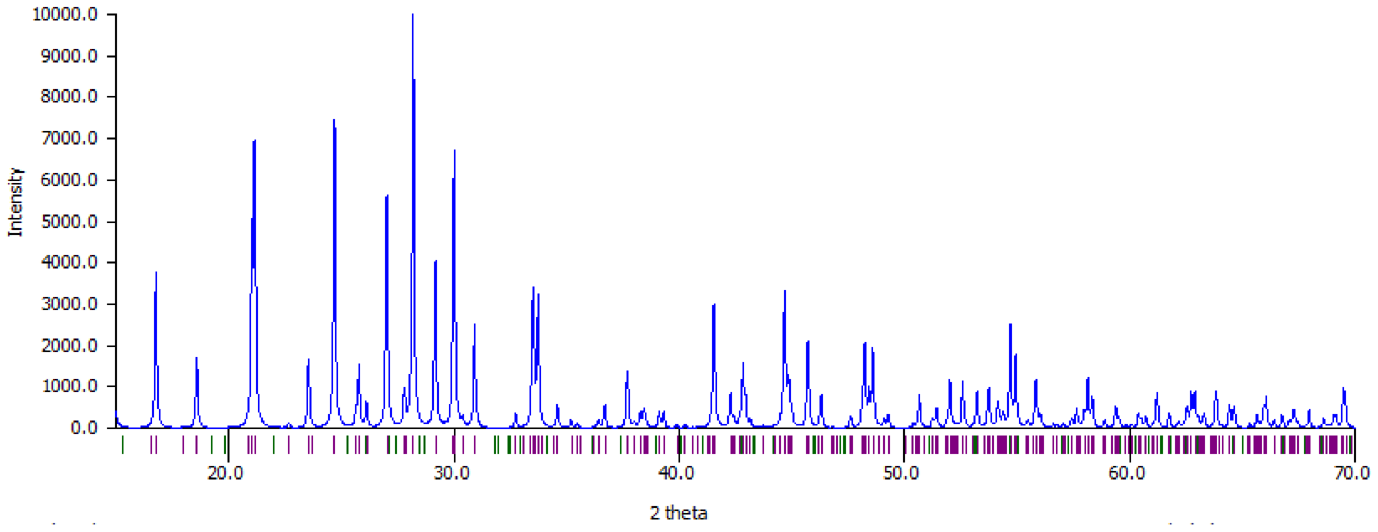

Figure S40. Simulated PXRD pattern of $Na_2WO_4 \cdot 2H_2O$ (PDF# 01-085-9605).

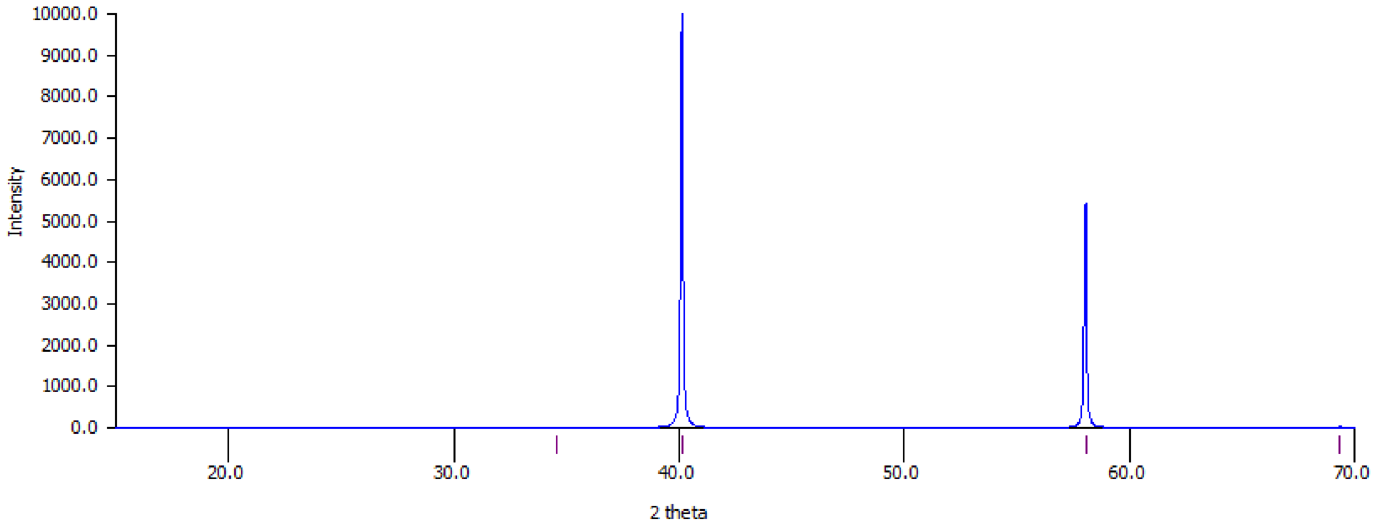

Figure S41. Simulated PXRD pattern of Na_2TiO_3 (PDF# 01-080-6123).

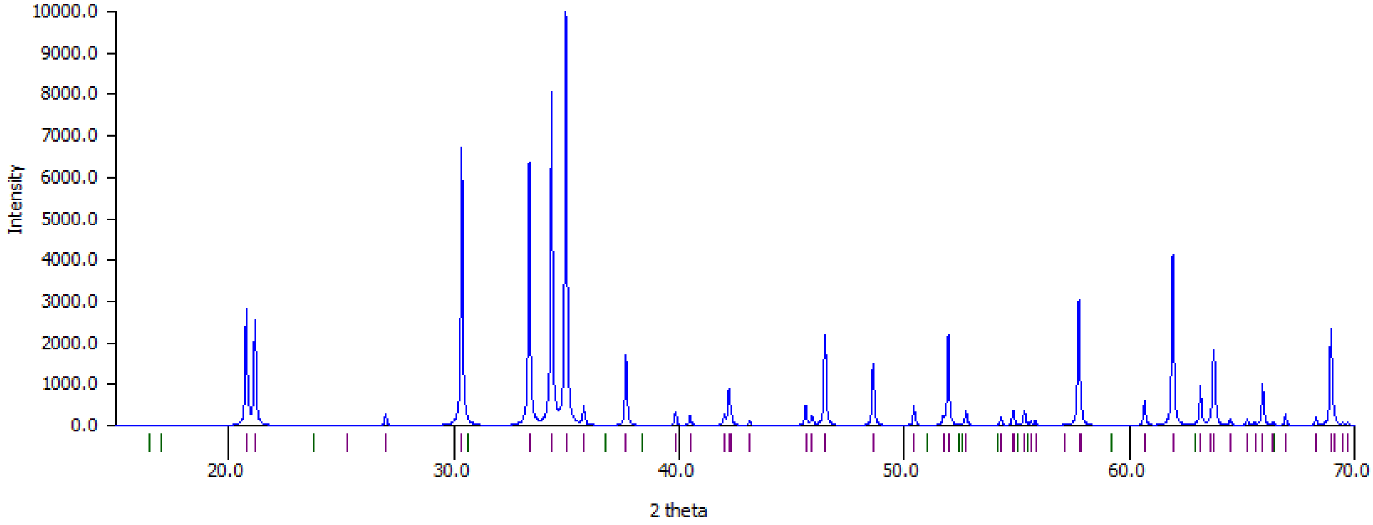

Figure S42. Simulated PXRD pattern of $NaAlO_2$ (PDF# 01-090-0702).

8. Isolation and Characterization of Alkali Metal Fluorides

8.1. Sodium Fluoride

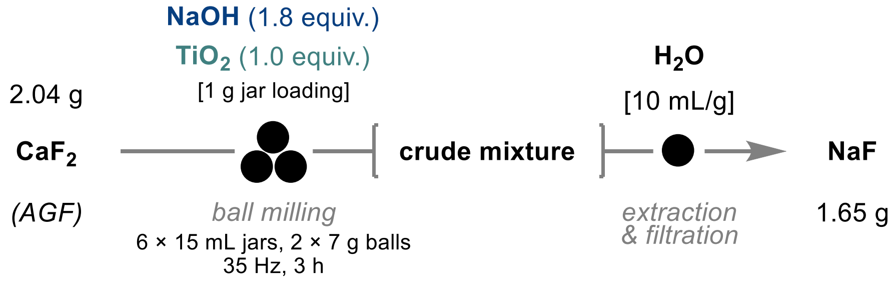

Six 15 mL stainless steel milling jars were charged with two hardened chrome steel bearings (2 × 7 g), AGF (340 mg, 1 equiv.), NaOH (313 mg, 1.8 equiv.), and TiO_2 (347 mg, 1.0 equiv.) each. The jars were then closed and securely fitted to the mill which was set for 3 h at a frequency of 35 Hz. Upon completion, the jars were opened and the powder was quantitatively transferred to a 100 mL beaker (the jars were rinsed with H_2O). The crude solid mixture was then extracted with H_2O (60 mL) for 30 min at room temperature, filtered through filter paper and washed with H_2O (3 × 30 mL). The filtrate was concentrated under reduced pressure, the obtained solid was washed with MeOH (10 mL), and dried in vacuo with a heat gun to give NaF as an off-white powder (1.65 g, 84% yield), which was analyzed by PXRD.

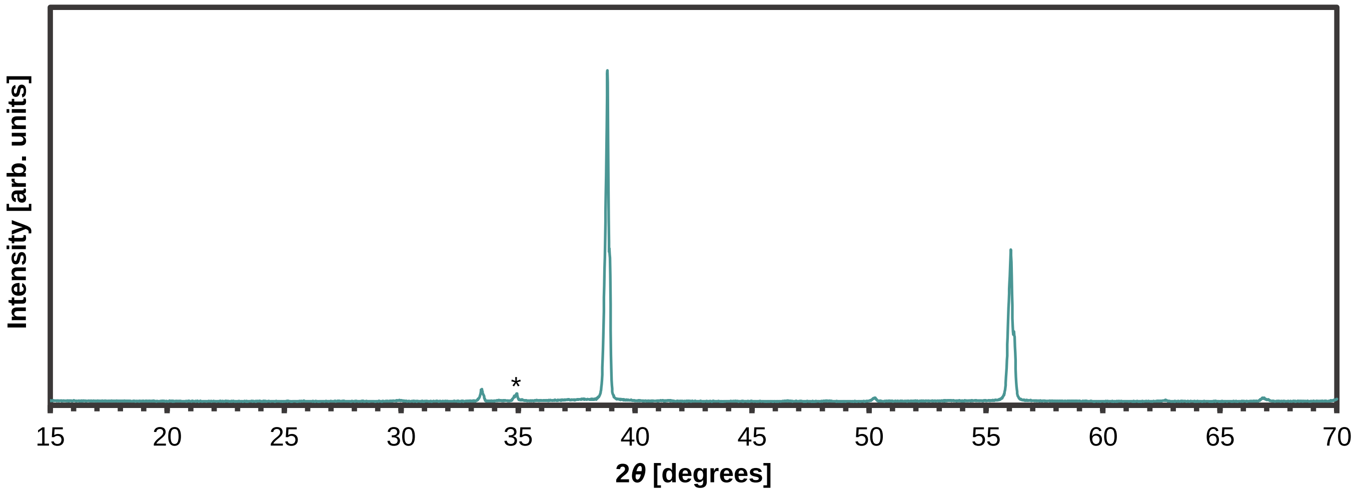

Figure S43. PXRD trace of isolated NaF (asterisks denote impurities). PDF# NaF: 01-075-0448.

An aliquot of the obtained NaF (14.8 mg) and sodium triflate (14.1 mg) as internal standard was dissolved in D_2O (10 atom% D, 1 mL), and analyzed by quantitative ^{19}F NMR spectroscopy to determine the purity to be >99%.

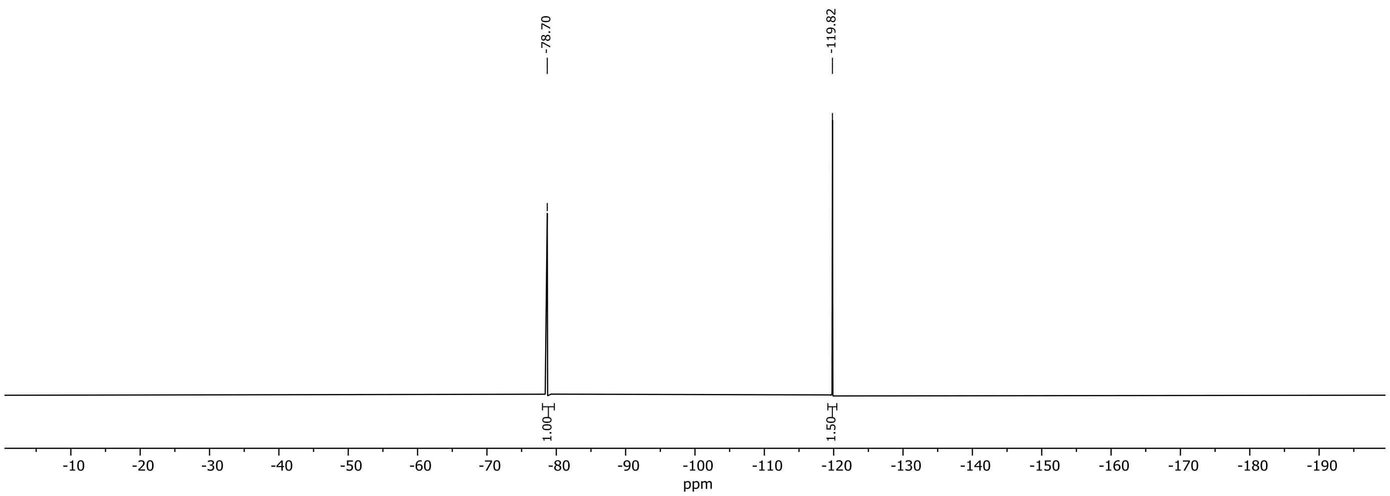

Figure S44. Quantitative ^{19}F NMR spectrum of 14.8 mg NaF (δ = −119.8 ppm) and 14.1 mg NaOTf (δ = −78.7 ppm, as an internal standard) in D_2O (10 atom% D).

Elemental analysis of a NaF sample (and a commercial reference sample) was performed by Mikroanalytisches Laboratorium Kolbe in duplicates and mean values are reported.

Table S18. Elemental analysis (n = 2) of isolated and commercial NaF.

NaF	purity [%]	F [wt%]	Na [wt%]	Ti [ppm]	Fe [ppm]
isolated (*this work*)	98.28	44.75	53.53	1135	597
Merck (≥99%)	99.87	45.20	54.67	n.d.	n.d.
theoretical	100	45.25	54.75	n.a.	n.a.

n.d. = not determined; n.a. = not applicable.

Thermogravimetric analysis (20 °C·min^{-1} ramp) of a NaF sample (approx. 50 mg) indicated <0.1% mass loss.

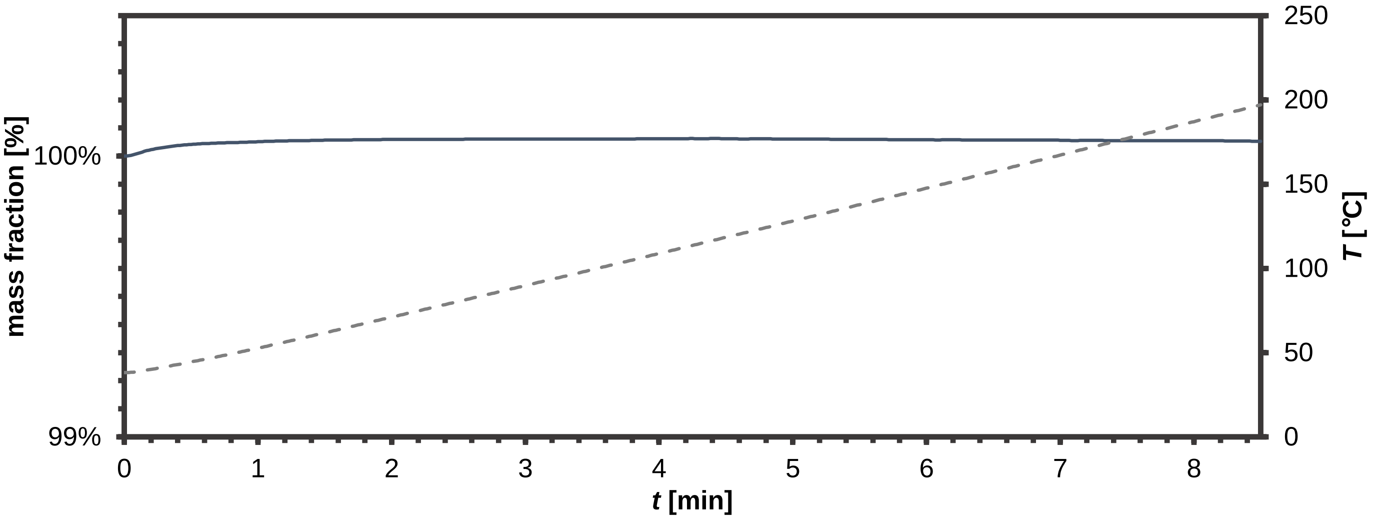

Figure S45. Thermogravimetric analysis (TGA) of isolated NaF (solid line refers to the mass fraction of the sample; dashed line indicates temperature gradient).

8.2. Sodium Fluoride (Decagram Scale)

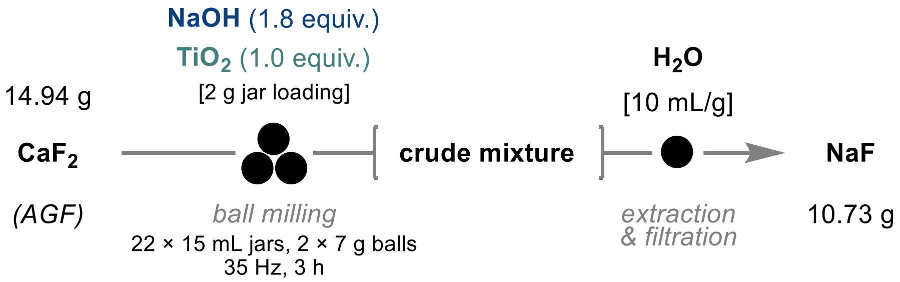

Twenty-two 15 mL stainless steel milling jars were charged with two hardened chrome steel bearings (2 × 7 g), AGF (679 mg, 1 equiv.), NaOH (626 mg, 1.8 equiv.), and TiO_2 (695 mg, 1.0 equiv.) each. The jars were then closed and securely fitted to the mill which was set for 3 h at a frequency of 35 Hz. Upon completion, the jars were opened and the powder was quantitatively transferred to a 1 L conical flask (the jars were rinsed with H_2O). The crude solid mixture was then extracted with H_2O (440 mL) for 30 min at room temperature, filtered through filter paper and washed with H_2O (3 × 110 mL). The filtrate was concentrated under reduced pressure, the obtained solid was washed with MeOH (3 × 20 mL), and dried in vacuo with a heat gun to give NaF as an off-white crystalline powder (10.73 g, 74% yield), which was analyzed by PXRD.

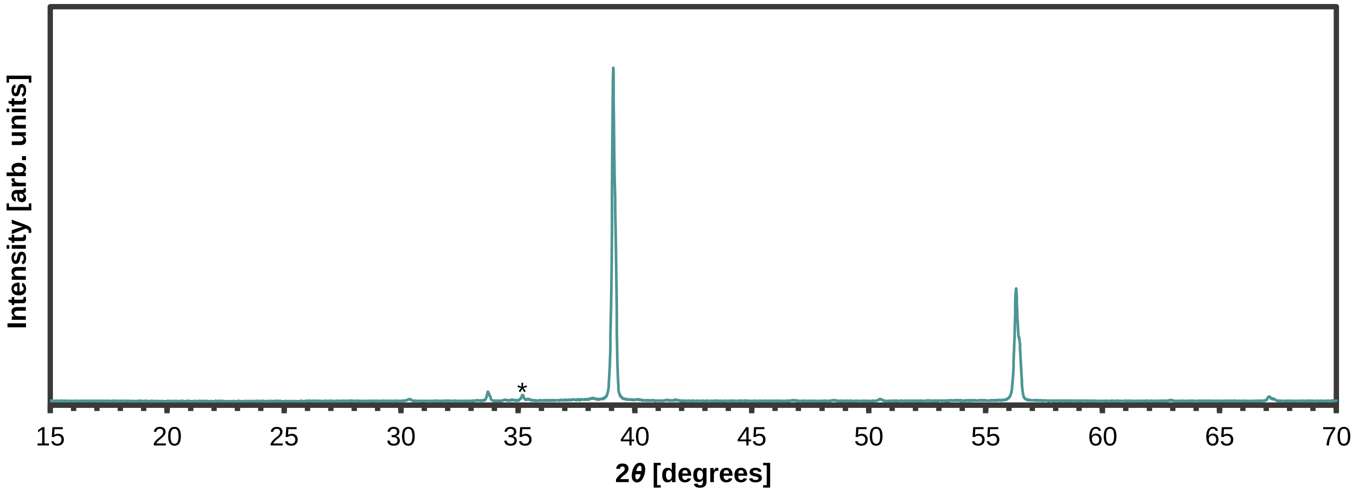

Figure S46. PXRD trace of isolated NaF (decagram scale) (asterisks denote impurities). PDF# NaF: 01-075-0448.

An aliquot of the obtained NaF (12.1 mg) and sodium triflate (14.4 mg) as internal standard was dissolved in D_2O (10 atom% D, 1 mL), and analyzed by quantitative ^{19}F NMR spectroscopy to determine the purity to be 99%.

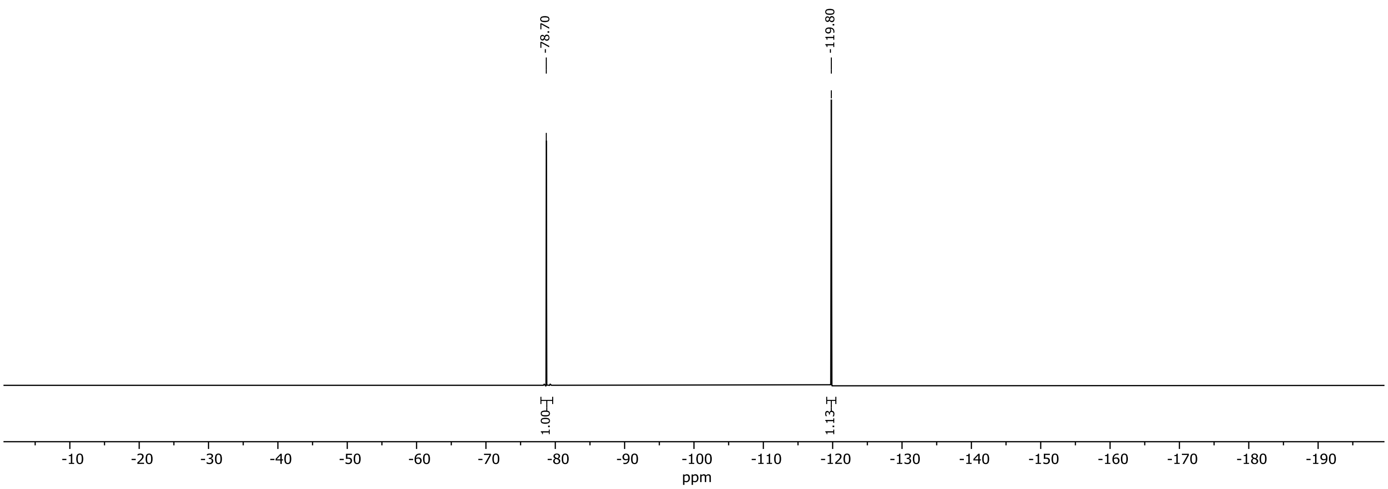

Figure S47. Quantitative ^{19}F NMR spectrum of 12.1 mg NaF (δ = −119.8 ppm) and 14.4 mg NaOTf (δ = −78.7 ppm, as an internal standard) in D_2O (10 atom% D).

Elemental analysis of a NaF sample (and a commercial reference sample) was performed by Mikroanalytisches Laboratorium Kolbe in duplicates and mean values are reported.

Table S19. Elemental analysis (n = 2) of isolated and commercial NaF (decagram scale).

NaF	purity [%]	F [wt%]	Na [wt%]	Ti [ppm]	Fe [ppm]
isolated (*this work*)	99.33	44.85	54.48	520	278
Merck (≥99%)	99.87	45.20	54.67	n.d.	n.d.
theoretical	100	45.25	54.75	n.a.	n.a.

n.d. = not determined; n.a. = not applicable.

Thermogravimetric analysis (20 °C·min^{-1} ramp) of a NaF sample (approx. 50 mg) indicated 0.6% mass loss.

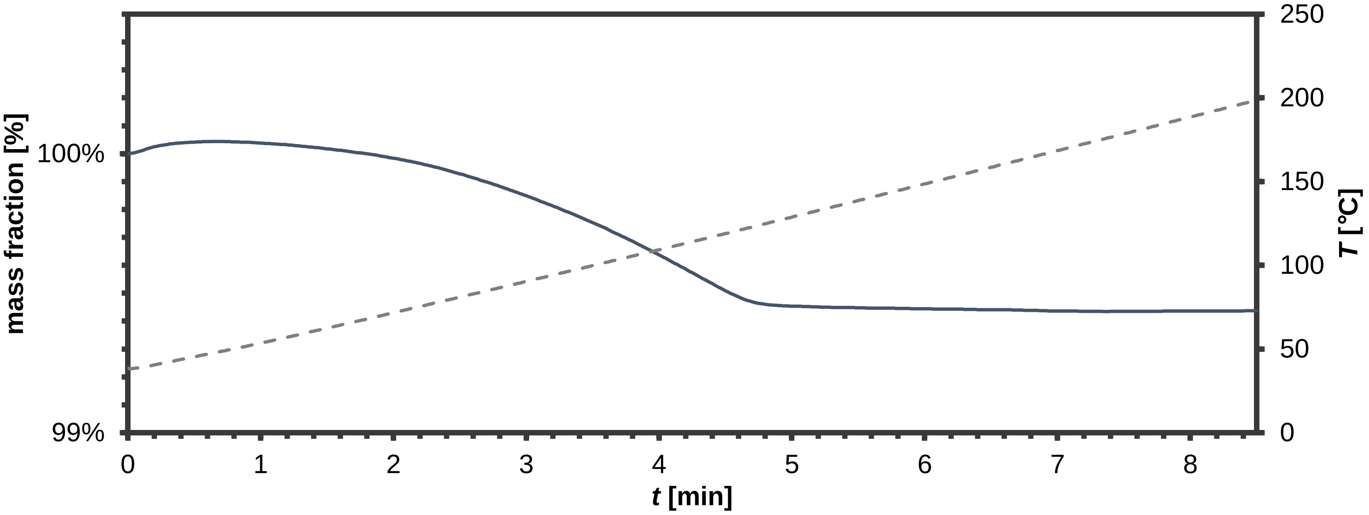

Figure S48. Thermogravimetric analysis (TGA) of isolated NaF (solid line refers to the mass fraction of the sample; dashed line indicates temperature gradient).

8.3. Lithium Fluoride

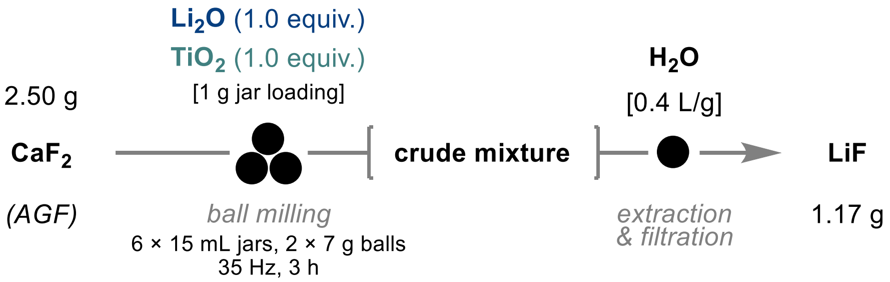

Six 15 mL stainless steel milling jars were charged with two hardened chrome steel bearings (2 × 7 g), AGF (416 mg, 1 equiv.), Li_2O (159 mg, 1.0 equiv.), and TiO_2 (425 mg, 1.0 equiv.) each. The jars were then closed and securely fitted to the mill which was set for 3 h at a frequency of 35 Hz. Upon completion, the jars were opened and the powder was quantitatively transferred to a 1 L conical flask (the jars were rinsed with H_2O). The crude solid mixture was extracted with H_2O (4 × 600 mL) at room temperature, and filtered through filter paper. The filtrate was concentrated under reduced pressure, the obtained solid was washed with H_2O (4 × 5 mL), and dried in vacuo with a heat gun to give LiF as an off-white solid (1.17 g, 71% yield), which was analyzed by PXRD.

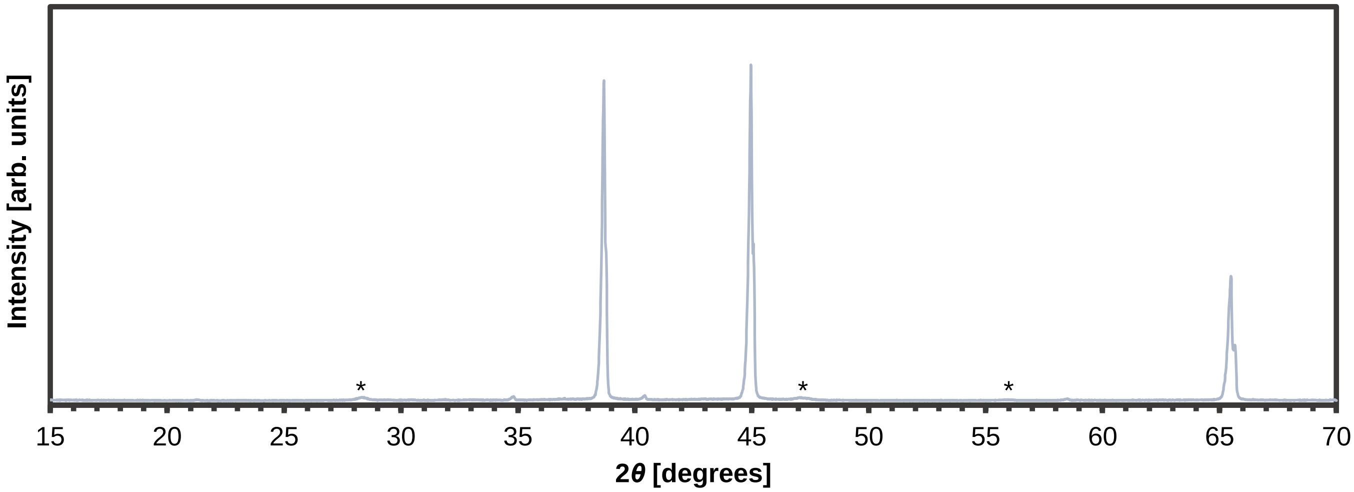

Figure S49. PXRD trace of isolated LiF (asterisks denote impurities). PDF# LiF: 01-085-8944.

Elemental analysis of a LiF sample (and a commercial reference sample) was performed by Mikroanalytisches Laboratorium Kolbe in duplicates and mean values are reported.

Table S20. Elemental analysis (n = 2) of isolated and commercial LiF.

LiF	purity [%]	F [wt%]	Li [wt%]	Ti [ppm]	Fe [ppm]
isolated (*this work*)	98.90	72.36	26.54	2177	407
Fluorochem (99.0%)	99.55	72.93	26.62	n.d.	n.d.
theoretical	100	73.24	26.76	n.a.	n.a.

n.d. = not determined; n.a. = not applicable.

Thermogravimetric analysis (20 °C·min^{-1} ramp) of a LiF sample (approx. 50 mg) indicated <0.1% mass loss.

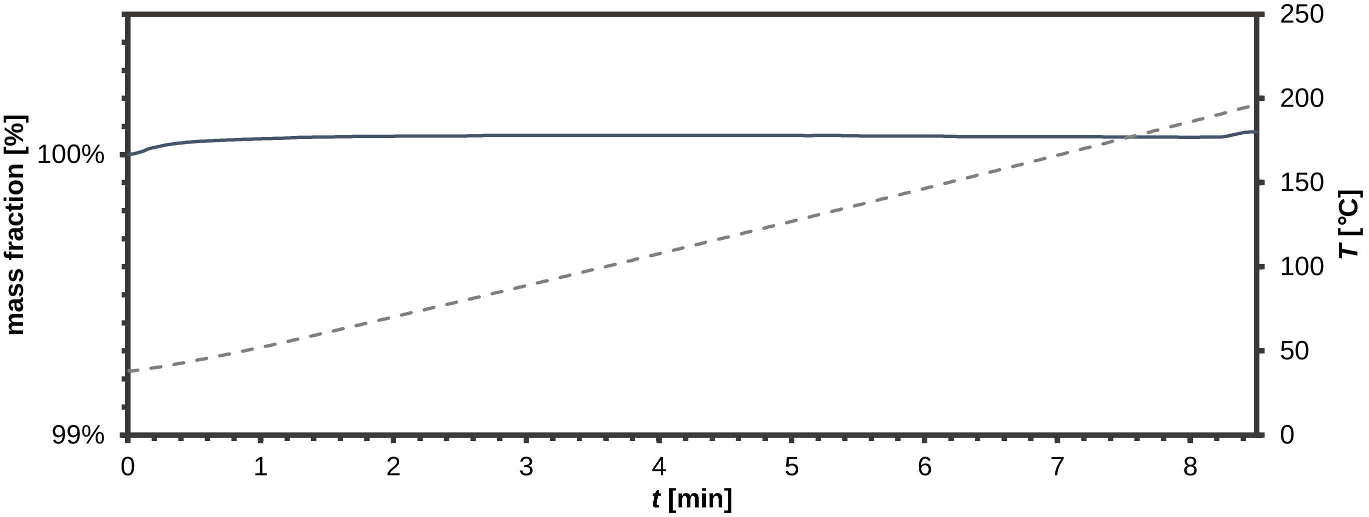

Figure S50. Thermogravimetric analysis (TGA) of isolated LiF (solid line refers to the mass fraction of the sample; dashed line indicates temperature gradient).

8.4. Potassium Fluoride

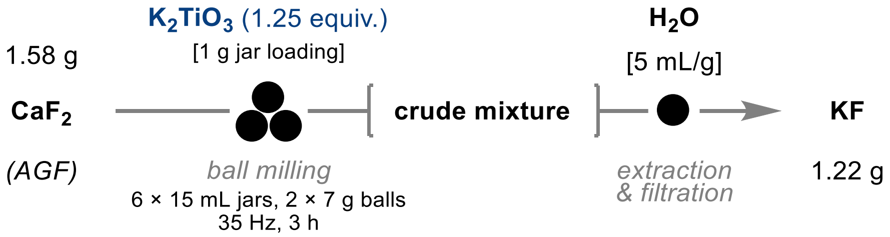

Six 15 mL stainless steel milling jars were charged with two hardened chrome steel bearings (2 × 7 g), AGF (264 mg, 1 equiv.), and K_2TiO_3 (736 mg, 1.25 equiv.) each. The jars were then closed and securely fitted to the mill which was set for 3 h at a frequency of 35 Hz. Upon completion, the jars were opened and the powder was quantitatively transferred to a 50 mL beaker (the jars were rinsed with H_2O). The crude solid mixture was then extracted with H_2O (30 mL) for 30 min at room temperature, filtered through filter paper and washed with H_2O (3 × 10 mL). The filtrate was concentrated under reduced pressure and dried in vacuo with a heat gun to give KF as an off-white powder (1.22 g, 52% yield), which was analyzed by PXRD.

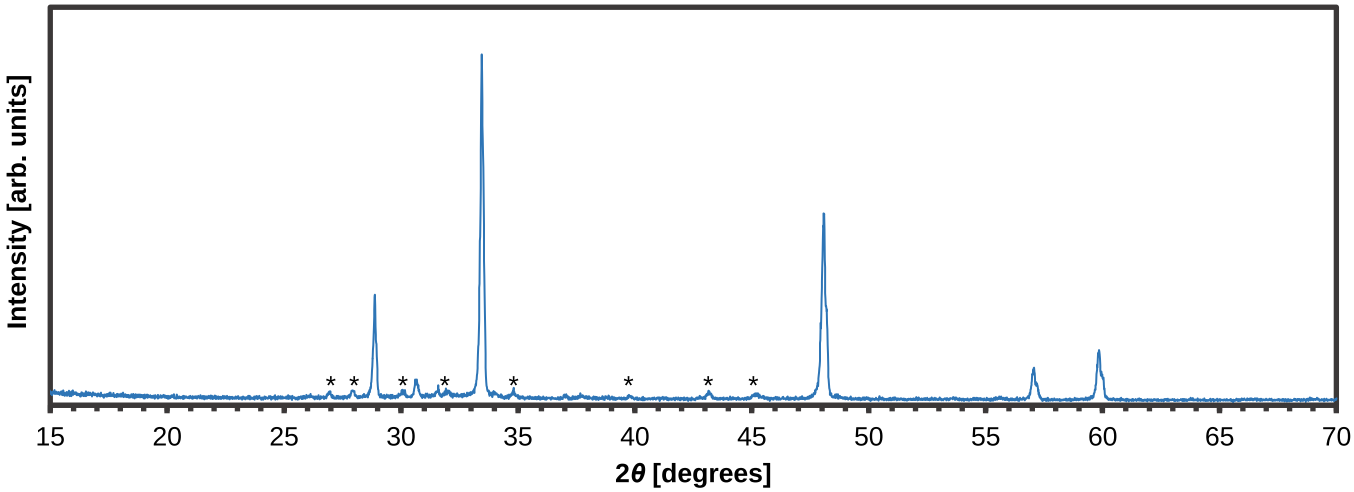

Figure S51. PXRD trace of isolated KF (asterisks denote impurities). PDF# KF 01-085-1314.

An aliquot of the obtained KF (14.3 mg) and sodium triflate (12.1 mg) as internal standard was dissolved in D_2O (10 atom% D, 1 mL), and analyzed by quantitative ^{19}F NMR spectroscopy to determine the purity to be 90%.

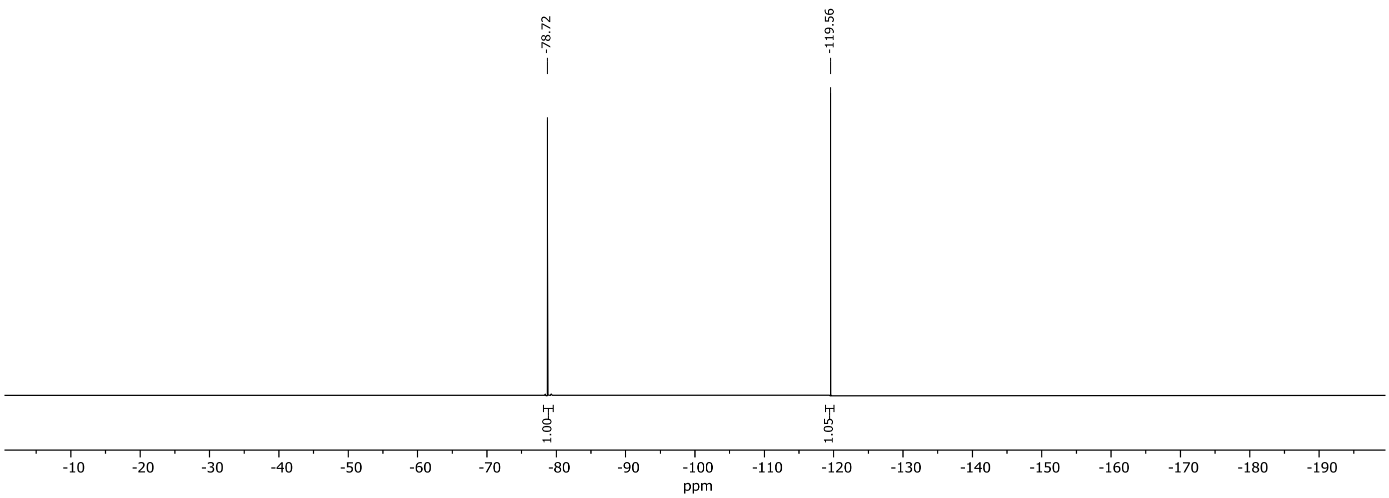

Figure S52. Quantitative ^{19}F NMR spectrum of 14.3 mg KF (δ = −119.6 ppm) and 12.1 mg NaOTf (δ = −78.7 ppm, as an internal standard) in D_2O (10 atom% D).

Elemental analysis of a KF sample (and a commercial reference sample) was performed by Mikroanalytisches Laboratorium Kolbe in duplicates and mean values are reported.

Table S21. Elemental analysis (n = 2) of isolated and commercial KF

KF	purity [%]	F [wt%]	K [wt%]	Ti [ppm]	Fe [ppm]
isolated (*this work*)	97.83	65.80	32.03	370	291
Thermo Fisher (99%)	99.44	66.94	32.50	n.d.	n.d.
theoretical	100	67.30	32.70	n.a.	n.a.

n.d. = not determined; n.a. = not applicable.

Thermogravimetric analysis (20 °C·min^{-1} ramp) of a KF sample (approx. 50 mg) indicated 2.5% mass loss.

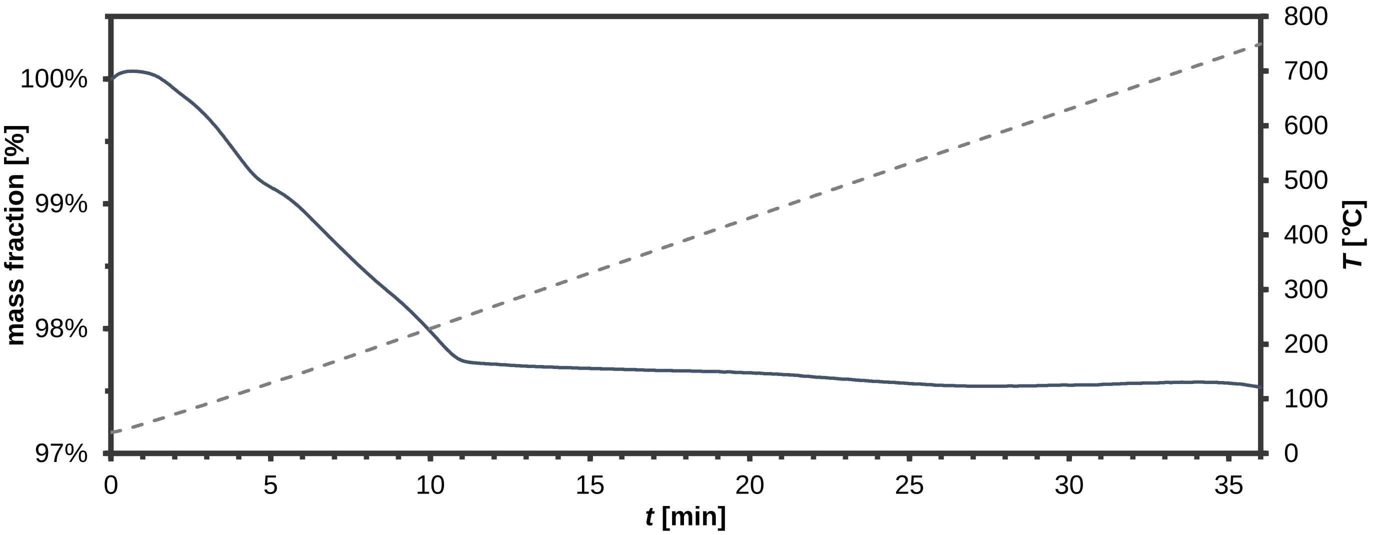

Figure S53. Thermogravimetric analysis (TGA) of isolated KF (solid line refers to the mass fraction of the sample; dashed line indicates temperature gradient).

9. References

[1] Griffin, J. M.; Yates, J. R.; Berry, A. J.; Wimperis, S.; Ashbrook, S. E. High-resolution ^{19}F MAS NMR spectroscopy: structural disorder and unusual *J* couplings in a fluorinated hydroxy-silicate. *J. Am. Chem. Soc.* **2010**, *132*, 15651-15660.

[2] Cromer, D. T.; Herrington, K. The structures of anatase and rutile. *J. Am. Chem. Soc.* **1955**, *77*, 4708-4709.

[3] Batchelder, D.; Simmons, R. Lattice constants and thermal expansivities of silicon and of calcium fluoride between 6 and 322 K. *J. Chem. Phys.* **1964**, *41*, 2324-2329.

[4] Sasaki, S.; Prewitt, C. T.; Bass, J. D.; Schulze, W. A. Orthorhombic perovskite $CaTiO_3$ and $CdTiO_3$: structure and space group. *Acta Crystallogr., Sect. C: Cryst. Struct. Commun.* **1987**, *43*, 1668-1674.

[5] Bragg, W. Crystal structure. *Nature* **1920**, *105*, 646-648.

[6] Straumanis, M. The precision determination of lattice constants by the powder and rotating crystal methods and applications. *J. Appl. Phys.* **1949**, *20*, 726-734.

[7] Stehr, H. Neubestimmung der Kristallstrukturen des dimorphen Natriumhydroxids, NaOH, bei verschiedenen Temperaturen mit Röntgenstrahl- und Neutronenbeugung. *Z. Kristallogr. Cryst. Mater.* **1967**, *125*, 332-359.

[8] Busing, W. R.; Levy, H. A. Neutron diffraction study of calcium hydroxide. *J. Chem. Phys.* **1957**, *26*, 563-568.

[9] Knight, K.; Darlington, C.; Wood, I. The crystal structure of $KCaF_3$ at 4.2 and 300 K: a re-evaluation using high-resolution powder neutron diffraction. *Powder Diffr.* **2005**, *20*, 7-13.

[10] Broch, E.; Oftedal, I.; Pabst, A. New assertion of the lattice parameter from KF, CsCl and BaF_2. *Z. Phys. B* **1929**, *3*, 209-214.

[11] Gerlach, W. Die Gitterstruktur der Erdalkalioxyde. *Z. Phys.* **1922**, *9*, 184-192.

[12] Marumo, F.; Isobe, M.; Iwai, S.; Kondō, Y. α-Form of sodium metavanadate. *Acta Crystallogr. B* **1974**, *30*, 1628-1630.

[13] Okada, K.; Morikawa, H.; Marumo, F.; Iwai, S. Sodium tungstate. *Acta Crystallogr. B* **1974**, *30*, 1872-1873.

[14] Krause, L.; Herbst-Irmer, R.; Sheldrick, G. M.; Stalke, D. Comparison of silver and molybdenum microfocus X-ray sources for single-crystal structure determination. *J. Appl. Crystallogr.* **2015**, *48*, 3-10.

[15] Liu, Y.; Zhao, W.; Wang, W.; Yang, X.; Chu, J.; Xue, T.; Qi, T.; Wu, J.; Wang, C. Study on the transformation from NaCl-type Na_2TiO_3 to layered titanate. *J. Phys. Chem. Solids* **2012**, *73*, 402-406.

[16] Proskurnina, N.; Voronin, V.; Shekhtman, G. S.; Kabanova, N. Crystal structure of $NaFeO_2$ and $NaAlO_2$ and their correlation with ionic conductivity. *Ionics* **2020**, *26*, 2917-2926.

[17] Haynes, W. M. *CRC Handbook of Chemistry and Physics*; CRC Press, 2014.

[18] Huheey, J. E. K., E. A.; Keiter R. L. *Inorganic Chemistry*; Harper Collins College Publishers, 1993.

[19] Yoder, C. H.; Flora, N. J. Geochemical applications of the simple salt approximation to the lattice energies of complex materials. *Am. Mineral.* **2005**, *90*, 488-496.

[20] Bartel, C. J.; Sutton, C.; Goldsmith, B. R.; Ouyang, R.; Musgrave, C. B.; Ghiringhelli, L. M.; Scheffler, M. New tolerance factor to predict the stability of perovskite oxides and halides. *Sci. Adv.* **2019**, *5*, eaav0693.

[21] Smith, K. A.; Burum, D. P. Application of fluorine-19 CRAMPS to the analysis of calcium fluoride/fluoroapatite mixtures. *J. Magn. Reson.* **1989**, *84*, 85-94.

[22] Miller, J. M. Fluorine-19 magic-angle spinning NMR. *Prog. Nucl. Mag. Res. Sp.* **1996**, *28*, 255-281.

[23] Hayashi, S.; Hayamizu, K. Accurate determination of NMR chemical shifts in alkali halides and their correlation with structural factors. *B. Chem. Soc. Jpn.* **1990**, *63*, 913-919.

[24] Schaller, T.; Dingwell, D. B.; Keppler, H.; Knöller, W.; Merwin, L.; Sebald, A. Fluorine in silicate glasses: a multinuclear nuclear magnetic resonance study. *Geochim. Cosmochim. Ac.* **1992**, *56*, 701-707.

[25] Kreinbrink, A. T.; Sazavsky, C. D.; Pyrz, J. W.; Nelson, D. G.; Honkonen, R. S. Fast-magic-angle-spinning ^{19}F NMR of inorganic fluorides and fluoridated apatitic surfaces. *J. Magn. Reson.* **1990**, *88*, 267-276.

[26] Bureau, B.; Silly, G.; Buzaré, J.; Emery, J. Superposition model for ^{19}F isotropic chemical shift in ionic fluorides: from basic metal fluorides to transition metal fluoride glasses. *Chem. Phys.* **1999**, *249*, 89-104.
